# Pragmatic cluster-randomized trial of home-based preventive treatment for TB in Ethiopia and South Africa (CHIP-TB)

**DOI:** 10.1186/s13063-023-07514-7

**Published:** 2023-07-25

**Authors:** Akash Malhotra, Bareng Aletta Sanny Nonyane, Evan Shirey, Christiaan Mulder, Piotr Hippner, Fiseha Mulatu, Andani Ratshinanga, Petros Mitiku, Silvia Cohn, Gideon Conradie, Violet Chihota, Richard E. Chaisson, Gavin J. Churchyard, Jonathan Golub, David Dowdy, Hojoon Sohn, Salome Charalambous, Ahmed Bedru, Nicole Salazar-Austin

**Affiliations:** 1 Department of Epidemiology, Johns Hopkins University Bloomberg School of Public Health, Baltimore, MD USA; 2 Department of International Health, Johns Hopkins University Bloomberg School of Public Health, Baltimore, MD USA; 3 Department of Pediatrics, Johns Hopkins University School of Medicine, Baltimore, Maryland USA; 4 Department of TB Elimination and Health System Innovations, KNCV Tuberculosis Foundation, The Hague, The Netherlands; 5 Amsterdam Institute for Global Health and Development, Amsterdam University Medical Centres, Amsterdam, The Netherlands; 6 The Aurum Institute, Parktown, Johannesburg, South Africa; 7 KNCV Tuberculosis Foundation, Addis Ababa, Ethiopia; 8 Department of Medicine, Johns Hopkins University School of Medicine, Baltimore, MD USA; 9 Department of Medicine, Vanderbilt University, Nashville, TN USA; 10 Department of Preventive Medicine, Seoul National University College of Medicine, Seoul, South Korea; 11 Yale School of Public Health, Division of Epidemiology of Microbial Diseases, New Haven, CT USA

**Keywords:** TB preventive treatment, TPT, Contact investigation, Household contact management, Pediatric TB, Pragmatic implementation trial, Ethiopia, South Africa, Tuberculosis

## Abstract

**Background:**

Each year, 1 million children develop TB resulting in over 200,000 child deaths. TB preventive treatment (TPT) is highly effective in preventing TB but remains poorly implemented for household child contacts. Home-based child contact management and TPT services may improve access to care. In this study, we aim to evaluate the effectiveness and cost-effectiveness of home-based contact management with TPT initiation in two TB high-burden African countries, Ethiopia and South Africa.

**Methods:**

This pragmatic cluster randomized trial compares home-based versus facility-based care delivery models for contact management. Thirty-six clinics with decentralized TB services (18 in Ethiopia and 18 in South Africa) were randomized in a 1:1 ratio to conduct either home-based or facility-based contact management. The study will attempt to enroll all eligible close child contacts of infectious drug-sensitive TB index patients diagnosed and treated for TB by one of the study clinics. Child TB contact management, including contact tracing, child evaluation, and TPT initiation and follow-up, will take place in the child’s home for the intervention arm and at the clinic for the control arm. The primary outcome is the cluster-level ratio of the number of household child contacts less than 15 years of age in Ethiopia and less than 5 years of age in South Africa initiated on TPT per index patient, comparing the intervention to the control arm. Secondary outcomes include child contact identification and the TB prevention continuum of care. Other implementation outcomes include acceptability, feasibility, fidelity, cost, and cost-effectiveness of the intervention.

**Discussion:**

This implementation research trial will determine whether home-based contact management identifies and initiates more household child contacts on TPT than facility-based contact management.

**Trial registration:**

NCT04369326. Registered on April 30, 2020.

**Supplementary Information:**

The online version contains supplementary material available at 10.1186/s13063-023-07514-7.

## Background

Tuberculosis (TB) and HIV remain among the top10 causes of child mortality in sub-Saharan Africa [1]. Each year, 1 million children develop TB resulting in over 200,000 child deaths [2]. TB preventive treatment (TPT) is highly effective, preventing >90% of TB disease among children adherent to treatment [3], but remains poorly implemented. In 2018, the UN High-Level Meeting on TB set ambitious targets to enhance TPT implementation [4]. Only 1.6 million household contacts less than 5 years of age (40% of target) and 600,000 household contacts 5 years and older (3% of target) were initiated on TPT from 2018 to 2021 [2]. The majority of household child contacts are either not identified or not linked to facility-based care, despite decentralized services [5, 6]. The World Health Organization (WHO) now endorses several short-course combination TPT regimens for household contacts, including 3HP (12 weekly doses of rifapentine and isoniazid) and 3RH (3 months of daily rifampicin and isoniazid) [7]. Both regimens hold promise for improved acceptability and treatment completion. Their effectiveness, however, will depend on improved implementation of contact management.

One third of TB-exposed children live in sub-Saharan Africa, where TB and HIV are syndemic. Children living with HIV are at higher risk of TB disease and suffer a disproportionate amount of TB-related mortality [8–11]. In 2021, more than 20,000 children living with HIV died of TB disease [12]. Children exposed to HIV may also be at higher risk of TB exposure, given adults in their household may also be living with HIV. Gaps throughout the HIV care continuum also limit the effectiveness of ART and have resulted in 160,000 new pediatric HIV infections and 97,000 AIDS-related child deaths in 2021 [13]. Through TB prevention services, we may be able to target and enhance access to pediatric HIV testing and prevention services among these high-risk children.

The World Health Organization (WHO) currently recommends household contact investigation for people newly diagnosed with TB in low- and middle-income countries, with an emphasis on pediatric contacts [14]. Although the aim of this policy has been to find previously undetected TB patients and reduce transmission, such investigations represent a missed opportunity to also start contacts without TB on TPT. WHO guidelines do not address the optimal implementation of TB contact investigation [14, 15]. The standard of care in most settings, or passive referral of pediatric contacts to the clinic by the index TB patient, has largely remained unsuccessful in practice. Implementation strategies to inform optimal implementation of contact management, which comprises contact investigation and TPT initiation and follow-up, among household child contacts less than 15 years are needed.

Community-based care delivery models for combined TB and HIV prevention may improve access to care for both services. Home-based contact tracing identifies more children under 5 years of age [6, 16]. The simplification of the pediatric screening process to a symptom-based approach allows for the possibility of home-based contact investigation and TPT initiation for asymptomatic children with referral of all symptomatic children. Integrated community-based pediatric TB and HIV prevention services, using existing community-based health teams in Ethiopia and South Africa, could improve access and reduce delays in the diagnosis, treatment, and prevention of TB and HIV in children. Furthermore, integrating these services into existing community-based services could make this approach cost-effective.

Few studies have evaluated the effect of home-based contact management on TPT uptake and completion in household child contacts [17, 18]. In this study, we aim to evaluate the effectiveness and cost-effectiveness of home-based contact management in two TB high-burden African countries.

### Objectives and endpoints

The primary objective of this study is to determine the effectiveness of home-based contact management, including TPT initiation and follow-up, using community-based healthcare workers to increase TPT initiation as compared to facility-based contact management. The primary endpoint is the cluster-level ratio of the number of household child contacts less than 15 years of age in Ethiopia and less than 5 years of age in South Africa initiated on TPT (3HP, 3RH, or 6H) per index patient, comparing the intervention to the control arm (Table 1). Because we anticipate heterogeneity in the epidemiology of TB and potential differences in the response to the intervention between countries, the primary outcome will be assessed across countries and within countries.

**Table 1:** Primary and secondary outcomes for the CHIP-TB trial

**Primary outcome**	**Outcome description**	
TPT initiation among child contacts	The cluster-level ratio of the number of household child contacts less than 15 years of age in Ethiopia and less than 5 years of age in South Africa initiated on TPT per index patient, comparing the intervention to the control arm	
**Secondary outcome**	**Outcome description**	
Child contact identification	The cluster-level ratio of the number of household child contacts less than 15 years of age in Ethiopia and less than 5 years of age in South Africa identified per index patient	
Continuum of care	The cluster-level proportions of estimated child contacts who progress through each stage of the pediatric TB prevention continuum of care (identification, screening, initiation, and completion) overall and by age, by TPT regimen, and by country. *estimated child contacts will be calculated using DHS data	
Treatment completion (as defined by WHO TPT Handbook)	• Proportion of children initiated on TPT who successfully completed TPT • Proportion of children initiated on TPT who were lost to follow-up • Proportion of children initiated on TPT with incident TB (treatment failure) • Proportion of children initiated on TPT who died • Proportion of children initiated on TPT who discontinued TPT due to: toxicity, drug-drug interaction, severe illness (e.g., malaria), pregnancy, or family preference *Treatment completion was defined as 11 doses in 16 weeks for 3HP and 68 doses in 4 months for 3RH [19].	
**Implementation outcome**	**Quantitative assessment**	**Qualitative assessment**
Acceptability of home-based contact management to all stakeholders (CG, HEW, TBF, PM)	• Proportion of identified households who agree to a home visit • Proportion declining HIV testing (South Africa only) • Proportion of children HIV tested by the community health team • Proportion of newly diagnosed HIV-positive children who are referred to the clinic for ART initiation	• Individual in-depth interviews (CG, HEW, TBF, PM)
**Fidelity** to the home-based contact management strategy	• Proportion of children initiated on the correct TPT regimen • Proportion of children initiated on the correct TPT dose	• Individual in-depth interviews (CG, HEW, TBF, PM)
**Feasibility** of home visits for CGs and community-based health teams	• Average number household visit attempts per household • Mean and median duration of each household visit • Proportion of index patients who refuse to provide an address or who provide an invalid/incorrect address	• Individual in-depth interviews (CG, HEW, TBF, PM)
**Cost and cost effectiveness**	• Mean difference in total patient and health system costs between the intervention and control arms • Incremental cost-effectiveness ratio (ICER) where effectiveness is modeled as the number of disability adjusted life years (DLYs) averted by the intervention in comparison with the standard of care.	• Structured interviews to assess implementation costs • Time and Motion observations of TB preventive care

Stakeholders: *CG* Caregiver, *HEW* Health extension worker, *TBF* TB focal persons, *PM* Program manager

The secondary objectives of the study are to evaluate: (1) the TPT continuum of care by country, TPT regimen (3RH and 3HP), and age (< 5 and 5–14 years); (2) acceptability, feasibility, and fidelity of home-based contact management and HIV preventive services in each setting; (3) TPT treatment outcomes, including tolerability and reported adherence; and (4) the cost-effectiveness of home-based versus facility-based child contact management (Table 1).

Data will be collected on staffing changes within the TB clinic and community health teams and stockouts of TPT medications, pyridoxine, and other supplies needed for pediatric contact management (specimen collection, file and registers, etc.), to understand trends in the data.

## Methods

### Study design

This is a pragmatic, parallel, cluster-randomized, controlled, superiority trial comparing a home-based versus a facility-based care delivery model for contact management. Clinics were randomized in a 1:1 ratio to conduct either home-based (intervention) or facility-based (standard of care) contact management. Randomization was stratified by country, local geography, and TB notifications. Given that the intervention is carried out by a clinic in its entirety, neither the patient, provider/outcome assessor nor the study team including data analysts will be masked to the study procedure.

Routine program data will be collected by healthcare workers and abstracted by the study team retrospectively. Prior to trial implementation, a standardized child contact management form will be implemented in both settings to standardize data collection by all facilities in both arms. The tool was adapted from a previous trial by each country team and in conjunction with the parent IMPAACT4TB project [6, 20, 21]. Children will be evaluated monthly until they complete TPT, as per the Ethiopian and South African National Guidelines. Concomitant interviews will not be prohibited.

### Study setting

**Fig. 1:**
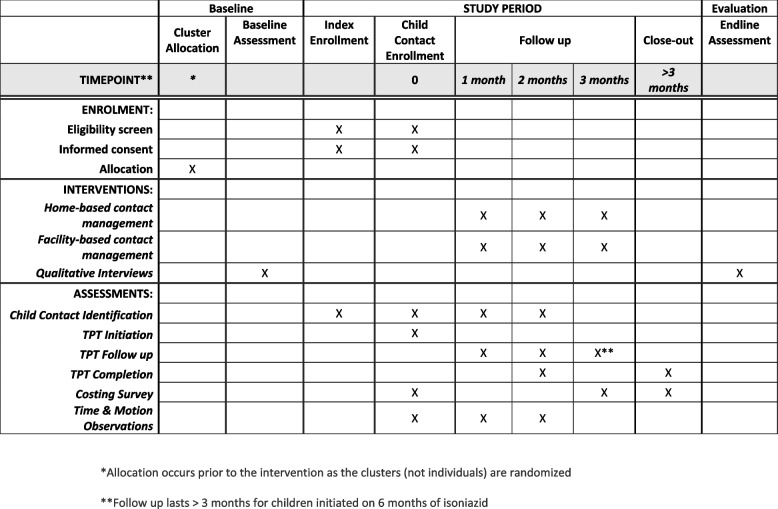
SPIRIT study schedule

This study will be conducted over 12 months in two sub-Saharan African settings with high-TB burdens: Ethiopia and South Africa. Both countries have decentralized TB services available at the local clinic. Neither country’s community-health teams currently conduct child contact management at the community-level. The schedule of the study is presented in Fig. 1.

In Ethiopia, each health facility is associated with 3–5 health posts in the community that serve 3–5000 persons in the community and are staffed by health extension workers. Health extension workers are community health workers who receive at least 18 months of comprehensive training and weekly supportive supervision from facility-based healthcare workers. The health extension workers diagnose and treat children with malaria, pneumonia, neonatal sepsis, and diarrhea with dehydration, provide childhood immunizations, perform mass TB screening, and provide directly observed treatment for TB index patients. In Ethiopia, HIV only partially drives the TB epidemic where only 5% of TB patients are living with HIV [2].

In South Africa, the Ward-Based Outreach Team (WBOT) system is used to provide home-based services [22]. The team includes a professional nurse or outreach team leader (OTL) and several community health workers assigned to different sections of the township. Community health workers provide TB screening and directly observed therapy. OTLs evaluate patients and initiate treatment. Contact management and referral is variably implemented at the home level. For the purposes of the study, implementation and research needs were balanced by hiring a research nurse who functioned as an OTL because health system nurses are not allowed to participate in research activities. Unlike Ethiopia, South Africa has high rates of co-infection where 58% of TB index patients are also living with HIV in 2019 [23].

### Study population

The study will attempt to enroll all close child contacts of individuals diagnosed and treated for infectious drug-sensitive TB in the study clinics. Good Clinical Practice (GCP)-trained study and clinic staff will enroll participants. Eligibility criteria for index patients and child contacts are pragmatically designed to align with the Ethiopian and South African National Guidelines regarding TPT eligibility. We will enroll index participants with pulmonary TB disease who (1) are 18 years and older, (2) attend a study clinic for TB care and live in the catchment area of that clinic, (3) are willing to have a home visit and disclose their TB diagnosis to household members, and (4) who provide informed consent. In South Africa, all index patients will need bacteriologic confirmation of pulmonary TB disease by smear, Xpert, and/or liquid mycobacterial culture. In Ethiopia, index patients may have bacteriologic confirmation or clinical diagnosis. Index patients in both countries will be excluded if there is evidence of rifampin and/or isoniazid resistance on Xpert or drug sensitivity testing from isolates identified on culture (late exclusion) or if the household has more than one index patient and whose child contacts have already participated in the study. Child contacts will be enrolled if (1) they are less than 15 years of age in Ethiopia or less than 5 years of age in South Africa, (2) they are a close contact of the index patient, and (3) their parent or legal guardian is willing to provide informed consent. Additionally, in Ethiopia, children 12 years and older must be willing to provide assent. Child contacts in both countries will be excluded if any index patient in their household has drug-resistant TB by either Xpert or drug sensitivity testing. Eligibility criteria will be adapted as country guidelines are updated.

### Cluster selection and randomization

The clinics chosen in both Ethiopia and South Africa are representative of typical clinics in their respective settings in terms of clinic size, catchment population, HIV co-infection, and provision of TB preventive treatment (Supplementary File 1). TPT initiation is poor in both countries, reaching an estimated 33% and 56% of contacts less than 5 years of age in Ethiopia and South Africa respectively in 2019 [23]. Both countries are part of the IMPAACT4TB project and were rolling out 3HP and/or 3RH for household child contacts [20].

The unblinded study statistician conducted constrained randomization balancing on baseline characteristics of the clinics that we believed would be associated with the primary outcome, including clinic geography and typical TB notifications [24]. We used STATA’s *cvcrand*function with the default balance metric which is the absolute value of the sum of the weighted differences in mean levels of covariates between the intervention and control arms [25, 26]. We assumed equal weights for all the covariates. In South Africa, the available characteristics were urban/rural stratification, sub-district and the number of TB diagnoses in the clinic averaged between 2019 and 2020 (an average was taken to account for anticipated recovery in TB notifications in 2021 after COVID-related lockdowns). Four of the clinics had much higher rates of TB diagnosis relative to the other 14 and we therefore created a size stratification so that these four clinics were placed in their own stratum. We ran *cvcrand* to generate up to 50,000 possible randomization sequences. In constrained randomization of clusters, one ought to not only consider balance on baseline covariates, but also *validity*of the set of acceptable randomization sequences from which a final one is selected [24]. The concept of validity implies that all possible pairs of clusters should be assigned to the same arm roughly 50% of the time among the sequences in the acceptable subset [24]. Too much deviation from this has a potential to inflate the Type 1 error rate. The subset of generated sequences with the lowest 25% of the balance metric values was considered a good tradeoff between reasonable covariate balance and validity. Our validity check within *cvcrand* showed that in this subset, the median proportion of times any two clinics were assigned the same study arm was 49% (min 27%, max 52%), which we considered acceptable. This subset of allocation sequences was presented at a public ceremony with representatives from participating clinics and other stakeholders. The ceremony was held within days of staff training and study initiation. Volunteers among attendees participated in drawing the final allocation sequence, and then participating clinics were informed of their study arm allocations. After clinic randomization, but before study initiation, it was found that one of the clinics in the control arm in South Africa did not directly provide TPT initiation services to children. A replacement with another facility with similar baseline characteristics and in the same geographical area was made.

In Ethiopia, data on 2020 TB notifications per clinic and region (Central, East, and South) were available to balance study arm allocations. The COVID pandemic did not significantly affect TB notifications in Ethiopia and TB notification data were therefore not averaged over time [2, 27]. We followed the same default *cvcrand* options as for South Africa. The randomization resulted in equal numbers of each region represented in each arm, and similar mean numbers of TB notifications (31 vs 33). In the subset of generated randomization sequences having the lowest 25% balance metric values, the median proportion of times any two clinics were assigned the same study arm was 43% (min 31%, max 60%). We considered this satisfactory validity, and the final sequence was randomly selected among these, and this selection was made by default within *cvcrand.* This sequence was sent to the coordinating center and site data managers. The study staff and other stakeholders in participating clinics were informed of the arm allocations in time to finalize trainings and initiate the study.

### Study description and procedures

#### Intervention arm

**Fig. 2:**
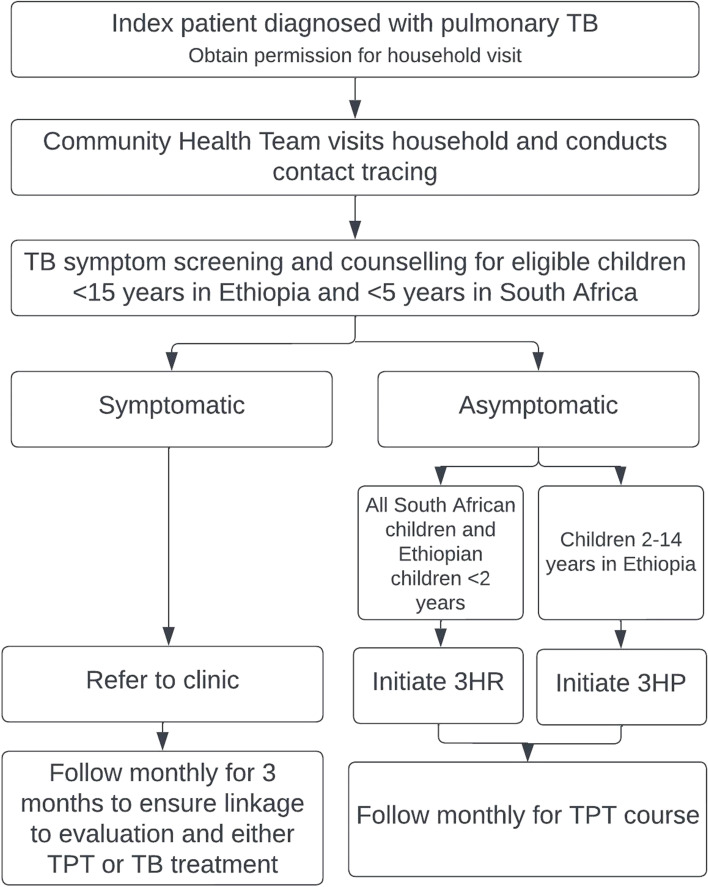
Integrated community-based TB prevention for household eligible child TB contacts. 3HP is 12 weekly doses of rifapentine and isoniazid; 3RH is 3 months of daily rifampicin and isoniazid

In the intervention arm, child TB contact management, including contact tracing, child evaluation, and TPT initiation and follow-up, will take place in the community at the child’s home (Fig. 2). Newly diagnosed eligible TB index patients will be asked to give consent to a home visit. The initial home visit will include TB symptom screening for all TPT-eligible children (< 15 years in Ethiopia and < 5 years in South Africa), and either TPT initiation for asymptomatic child contacts or clinic referral for all symptomatic child contacts. TB screening and TPT initiation will be conducted by either a research nurse (South Africa) or health extension worker (Ethiopia) in the home. Children who screen negative (0 of 8 symptoms present, see the “ TB screening and evaluation” section) will be initiated on TPT. Children who screen positive (any one symptom is present) will be counseled and referred to the local clinic for evaluation as per the local standard of care. The community health team (in Ethiopia) or the research nurse (in South Africa) will revisit the household monthly for three months to ensure linkage to care.

In Ethiopia, drug dispensing will occur in a two-step process where the health extension worker will first screen the child in the home and then return with a one-month supply of the appropriate TPT regimen and dose after discussion with the TB focal person at the clinic (task-shared). In South Africa, the anticipated TPT regimen based on reported contacts will be brought to the household by the nurse during the initial evaluation visit. If the child is symptomatic and not initiated on TPT, the stock will be returned to the clinic pharmacy. In both countries, the child will be weighed at their initial home visit. This weight will determine the dose for the 3-month duration of TPT. In Ethiopia, the full 3-month kit of TPT will be stored at the health post for each child to prevent stockouts from affecting individuals in the middle of their TPT course.

In South Africa, HIV testing will be offered and conducted in the household for all children 18 months and older with a rapid HIV test. Children under 18 months old will be assessed for HIV exposure by both self-report and evaluation of their health card. The care relating to prevention of mother-to-child transmission (PMTCT) will be reviewed including maternal antiretroviral status, infant prophylaxis, and infant testing. If they are not up to date with testing or have been lost to follow-up, the community health team will refer that infant/mother pair to the clinic. In Ethiopia, HIV testing will not be offered in the home as HIV testing all child TB contacts is not the standard of care given the fairly low proportion of HIV co-infected TB patients (5.2%) [2]. Table 2 summarizes context-specific differences in the intervention between Ethiopia and South Africa.

**Table 2:** Key design feature comparison between countries

	**Ethiopia**	**South Africa**
Study population – index patients	Clinically diagnosed and bacteriologically confirmed pulmonary TB patients	Bacteriologically confirmed pulmonary TB patients
Study population – child contacts	Children < 15 years of age	Children < 5 years of Age
TPT regimen^a^	3HP for those ^3^ 2 years 3RH for those < 2 years 6H for those on ART	3RH for those 0-5 years 6H for those on ART
Community-based care model	Health extension worker based at health post in the community	Outreach team leader (professional nurse) teamed with community health worker
TPT initiation and follow-up	Performed by health extension worker; **Task-shared** with clinic TB focal person	Performed by professional nurse; **Task-shifted** from clinic TB focal nurse to a community-based health nurse
Persons responsible for TPT initiation and follow-up during trial	Utilized health system’s TB focal persons and health extension workers	Research nurse and clinic’s TB focal person
HIV testing	Not performed at household visits	Performed at household visits

^a^3HP is 12 weekly doses of rifapentine and isoniazid; 3RH is 3 months of daily rifampicin and isoniazid; 6H is 6 months of daily isoniazid

#### Control arm

**Fig. 3:**
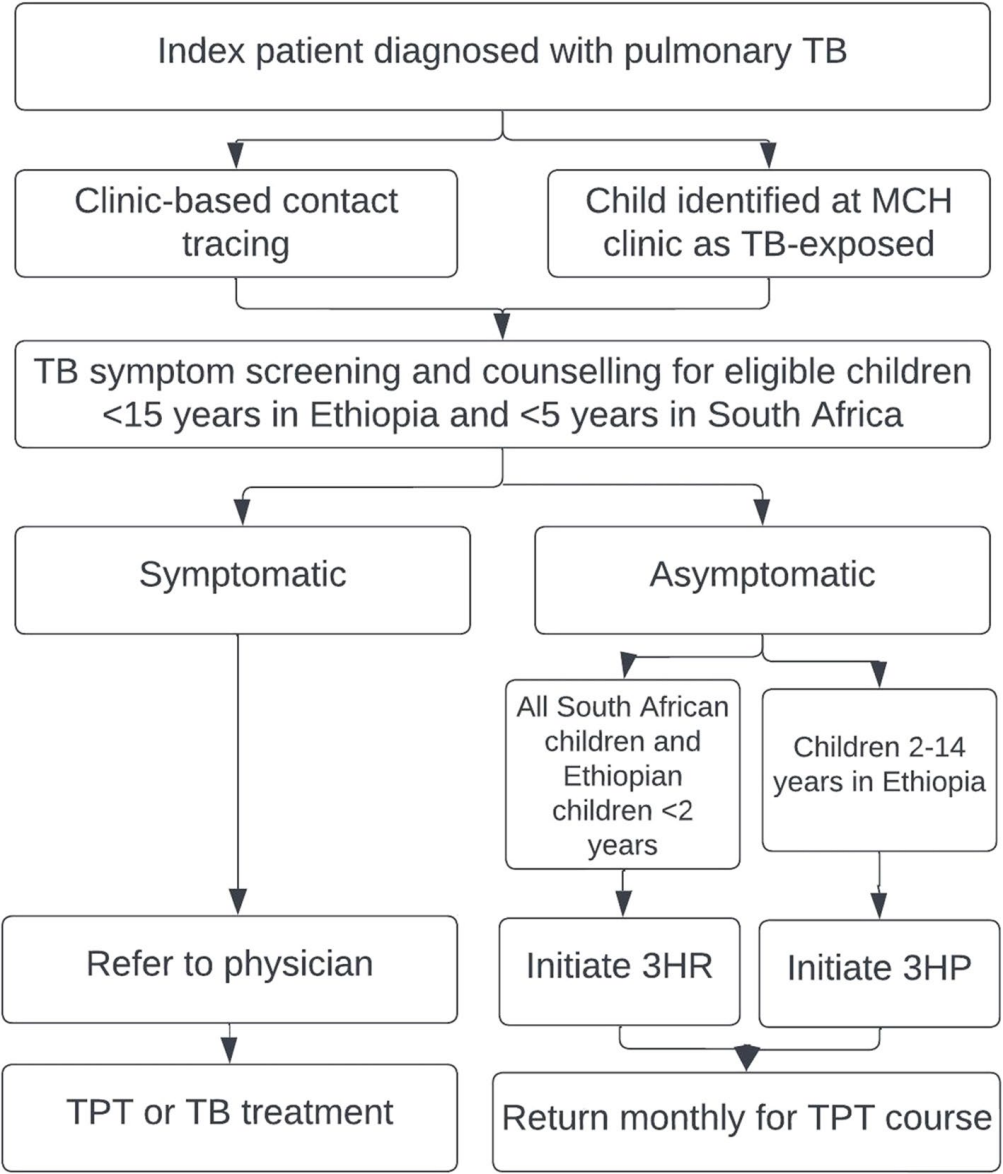
Facility-based TB prevention for household eligible child TB contacts. 3HP is 12 weekly doses of rifapentine and isoniazid; 3RH is 3 months of daily rifampicin and isoniazid

In the control clinics, child TB contact investigations and initiation of TPT will all take place at the facility (Fig. 3). Newly diagnosed infectious TB index patients will be encouraged to bring their household child contacts potentially eligible for TPT by age (< 15 years in Ethiopia and < 5 years in South Africa) to the clinic for TB screening. Community health workers may be used to identify child contacts, encourage clinic-based evaluation, and assess children who may be lost to follow-up, as is currently the standard of care. Children identified as TB contacts during routine screening in the MCH clinic may also be referred to the TB clinic for contact investigation and management. Medications will be dispensed by the TB focal person or the pharmacy, as per the standard of care within each control clinic.

#### TB screening and evaluation

In both the intervention and control arms, eligible children will be clinically evaluated using the national TB screening tool. TB screening will focus on major pediatric symptoms including fever, cough of 2 weeks or more, wheezing, reduced playfulness or unusual fatigue, lethargy, visible mass in the neck, weight loss, or failure to thrive based on clinical report and/or the growth chart in the child’s medical record and immunization record (i.e., Road to Health Booklet). Ultimately the child will either be started on TPT (if TB disease is ruled out) or TB treatment.

#### Treatment follow-up and adherence

Children initiated on TPT will be followed at home (intervention) or in clinic (control) monthly for the duration of TPT. Adherence will be measured by return visits and pill counts in both groups. Children will also be re-screened for TB symptoms during each follow-up visit.

#### Household costing and time-and-motion

Within 2 weeks from the first TB screening and evaluation, research assistants will conduct a standardized survey with the child’s caregiver about costs incurred to either host the community health team (intervention arm) or to visit the health facility (control arm). In this first survey, we will also inquire about their household’s asset ownership, living conditions, and educational attainment. Upon TPT completion, a follow-up costing survey will capture estimated costs on follow-up care and management of TPT-related adverse events, including an assessment of catastrophic costs (costs totaling ≥20% of annual household income) [28].

For a subset of participants, a time-and-motion observation will be conducted by a research assistant at either the household or the health facility to capture the time the participant spends on each activity (screening, enrolment, counseling, etc.) at initiation and/or follow-up.

#### TPT regimen

In Ethiopia, all clinics are participating in IMPAACT4TB where 3HP is being rolled out [20]. All contacts 2 years and older will be initiated on 3HP while children < 2 years will be initiated on 3RH. In South Africa, the clinics are not participating in IMPAACT4TB and all children < 5 years will be started on 3RH. In both countries, if there is a contraindication to initiating rifamycins (e.g., drug interaction including antiretroviral therapy), 6H will be used.

### Sample size considerations

We considered sample size estimation for the South Africa and Ethiopia sites separately because of the differences in TB epidemiology in the two countries. Sample size calculations were based on the primary outcome which is the clinic-level number of child contacts under age 15 years (Ethiopia) or under 5 years (South Africa) initiating TPT per index patient, compared between the two study arms. We expect there to be a harmonic mean of 51 and 32 diagnosed TB patients per clinic during the study period, in South Africa and Ethiopia respectively. We also expect 20% refusals, withdrawals, or loss to follow-up leading to an average of 40 and 26 TB patients, as reported by the respective TB programs. An estimate of the coefficient of variation (CV) was obtained from a previous contact tracing study in South Africa which was 0.35 [6]. We assumed a similar amount of between-cluster variability in Ethiopia, and we also evaluated how the required sample size changed if we considered a slightly higher CV of 0.4. Prior household-based contact tracing studies and 2016 DHS data showed that households of index TB patients in South Africa and Ethiopia, respectively, have 1.8 and 2.1 children under the age of 15, and we expect that 30% would initiate TPT under the standard of care [6, 29–32]. We hypothesize that the intervention would double the number of children per TB patient initiated on TPT — from 0.5 (=1.8×0.3) to 1 in South Africa, and 0.6 (=2.1×0.3) to 1.2 in Ethiopia. A total of 18 (9 per arm) clinics in each country would be required to detect this effect with 80% power at a 5% level of significance assuming a CV of 0.35. A larger CV of 0.4 would require 20 and 22 clinics in South Africa and Ethiopia, respectively.

### Data collection

#### Outcome data

Clinical outcome data will be extracted from patient records maintained by the community health teams and the clinic. Outcome variables that will be retrospectively extracted from the child contact file include presence of symptoms (binary, yes/no will be asked for each symptom), gastric aspirate/Xpert/culture results (if applicable), chest X-ray results (if applicable), the TPT or active TB treatment initiation date, the TPT regimen, planned and actual dates of all follow-up visits, TPT completion date, and reasons for early discontinuation including patient preference, pregnancy (adolescents), and severe disease including malaria or side effects including flu-like illness, hypersensitivity reactions, hepatotoxicity, and peripheral neuropathy (if applicable). We will collect information regarding the location of child contact screening, TPT initiation, and all subsequent follow-up visits. We recognize these outcomes are dependent on clinic staff compliance with study tools. Periodic monitoring of study tools will be performed, and additional training will be provided if study tools are not being used optimally.

#### Missing data

Our sample size calculations have adjusted for potential refusals or losses to follow-up, and thus we expect to achieve the same level of power if up to 20% of diagnosed TB patients either refuse to participate or are enrolled and do not complete the study. We anticipate that these losses will occur completely at random and thus inference based on remaining participants will be valid. Data will be abstracted from clinic records, and we expect some missing data. We will work with study staff to investigate any missing data related to study endpoints to determine if there is systematic missingness. If appropriate, suitable imputation methods will be applied for missing covariate data.

#### Costing and time-and-motion data

Costs will be measured prospectively from study data from the societal perspective and will include costs related to both health systems and patients. Health systems costs will be captured using an “ingredients” approach through a combination of budgetary review, key informant interviews, direct observation of healthcare workers (time-and-motion observations), and time-stamped case report forms [33]. Health systems costs will include labor, training, transport, infrastructure, supplies/consumables, and other administrative/support costs. Patient costs will include transport, lost wages, clinic fees (if applicable), food, and child/family care. Costs will be measured on up to 400 index households (up to 100 interviews per arm per country). Patient-level costs will be measured through a standardized questionnaire completed by the caregiver responsible for enrolled children. The questionnaire will use the “Tuberculosis patient cost surveys: a handbook” as a guide and will also include the global multidimensional poverty index [34, 35].

#### Qualitative data

Data were collected by trained study staff before and will be collected after the trial through in-depth interviews in English, Amharic, Afan Oromo, isiZulu, and Sepedi. Participants included and will include program managers, providers, community health team members, and child caregivers. Interviews will be transcribed, translated, and coded by experienced qualitative researchers. The baseline interview focused on perceptions of the planned intervention including barriers and facilitators to its implementation. Post-trial interviews will focus on the intervention arm and will assess each stakeholder groups’ perception of the intervention’s acceptability and feasibility along with lessons learned. All participants provided or will be asked to provide informed consent for participation in each interview. Confidentiality will be maintained by not using names of people, clinics, or locations within the interview and eliminating specified people or places during transcription. We plan to use thematic analysis guided by the Consolidated Framework for Implementation Research (CFIR) [36].

#### Process data

To assist in our understanding of how program characteristics may influence outcomes for a given clinic and overall, a research assistant will use a standardized tool to assess each clinic monthly for relevant stockouts and staff shortages relating to child contact management.

### Data management

A child contact management record will be implemented and maintained by healthcare workers. The child contact management record will be maintained at the health facility in both the intervention and control clinics. Additionally, in Ethiopia, health extension workers will maintain a record in the community at the health post (intervention arm only). These data will be synced with the clinic file monthly. Data will be extracted retrospectively from these routine clinic charts either onto clinical research forms (Ethiopia) or directly into a REDCap database (South Africa), no less than one month after the clinic visit to allow sufficient time for charting.

Data will be entered and maintained on a secure, password-protected, web-based REDCap database with multisite access with secure web authentication and secure sockets layer encryption. The data constitutes a limited data set and does not include identifiers like name, date of birth, address or phone number, medical record number, and identity number.

This small, unmasked study presents minimal risk to study participants and therefore does not meet criteria for a data safety and monitoring board.

### Quality management

Quality control procedures will include a review of each clinical research form for accuracy and missing values, followed by queries to assess for missing values and consistency of responses across forms. A Johns Hopkins study coordinator will monitor the performance of all procedures to ensure they are in line with the study protocol including auditing both the abstraction and data entry processes with the purpose of identifying erroneous practices and to correct any drift in performance. Five percent of all abstracted files will be compared to their source documents for accuracy.

### Responsibilities of coordination center

Johns Hopkins University will be the coordinating center responsible for ensuring each site’s IRB has approved the protocol, consent/assent forms, and any amendments and will ensure teams have the most current versions of all documents (protocol, ICFs, SOPs, etc.). They will also manage the REDCap database and quality check the data. Finally, they will be responsible for evaluating and coordinating reporting on any protocol events.

### Statistical analysis

#### Primary and secondary outcome analyses

Summary statistics of the enrolled index patients will be presented stratified by arm and study site. Analysis of the primary outcome of the number of child contacts less than of 15 years of age in Ethiopia and less than 5 years of age in South Africa who initiate TPT per index patient diagnosed will be stratified by country. Cluster-level ratios will be compared between arms using an unpaired t-test which has been shown to be robust for a relatively small number of clusters [24]. The ratio of the study arm mean ratios and the corresponding 95% confidence interval will be calculated. A log-transformation of the cluster-level ratios will be applied before applying the *t*-test if the outcomes are highly skewed. If the number of index patients varies substantially between clinics, weighted (by clinic size) mean ratios will be used. If the sample size allows, we will compare the numbers of children started on TPT between arms stratified by TPT regimen (3HP vs 3RH) and children’s age groups (^3^5 vs <5 for Ethiopia).

Secondary outcomes to be compared between arms will be the number of child contacts identified (using index patient’s clinic-based contact tracing report, the child contact management file, and the community-level file maintained by the community health team) per index patient, and the proportions of these who are screened, initiated on TPT and who complete TPT. As done for the primary outcome, cluster-level summaries for secondary outcomes will also be compared between arms. Count outcomes will be compared using Poisson regression, and cluster-level proportions (after a log-transformation, if necessary) will be compared using a *t*-test.

We will provide summary statistics of TPT completion rates and losses to follow-up discontinuation (due to incident TB, side effects, transfer out, other reasons, or death) stratified by study arm and site. TPT completion will be defined as three monthly follow-up visits over a 4-month period (3HP or 3HR) or six monthly visits over a 9-month period (6H). Loss to follow-up will be defined as no return visit within three months of the child’s last recorded visit. Other reported outcomes will include new HIV diagnoses among children who are referred for ART initiation. All outcomes will be summarized by the study arm, and formal statistical tests will be conducted if the number of events observed allows. All data will be abstracted by study staff from index patient and child contact management files.

Acceptability of the intervention will be assessed as the proportion of child contacts who are identified and whose families agrees for them to be screened by community-based clinic staff (community health worker, professional nurse, type of provider dependent on the setting) in their home. We will also summarize reasons for the refusal of a household visit in the intervention arm using proportions among those approached. Fidelity to guidelines will be summarized as the proportion of TB contacts initiated on the correct TPT regimen by age and HIV status, and the proportion of TPT initiations with the correct dose for weight to measure guideline fidelity of community-based TB preventive care.

Process outcomes to be reported will include drug shortages and stockouts, stockouts of supplies needed for pediatric specimen collection, stockouts of TB and contact file and registers, other materials needed for the intervention (scale, etc.), staffing within the TB clinic and community health teams, use of community health teams to do contact tracing in control clinics, changes in key personnel including community health workers and TB nurses, and time the TB nurse and/or community health worker is pulled away from TB activities.

All analyses will be conducted using STATA software (StataCorp. 2021) [25].

#### Statistical plan for protocol non-adherence

The primary analysis will be intention to treat. We will explore who did and did not receive TPT initiation in the home vs clinic, as per their assigned arm. We will summarize such deviations. There are no planned per protocol or as treated analyses.

#### Costing and cost-effectiveness analysis

The endpoint of interest for the costing analysis will be a continuous outcome of household costs from enrolment until treatment completion/discontinuation, and health system costs through the course of the study period. We will calculate the mean difference in costs, comparing the intervention to the SOC arms.

The unit cost of diagnosis and treatment will be calculated from measured costs from the patient and health system perspectives. The outcome of interest will be the sum of patient plus health system costs, expressed as unit costs (e.g., cost per test, cost per TB treatment), under the intervention and under the standard of care. This outcome will not be subject to a formal statistical hypothesis test.

The modeled incremental cost-effectiveness ratio will be calculated from cost and effectiveness outcomes, including both patient and health system perspectives. The outcome of interest here will be the incremental cost per DALY (disability-adjusted life year) averted. This outcome will not be subject to a formal statistical hypothesis test.

All analysis will be conducted using R version 4.1.1 (R Foundation for Statistical Computing, Vienna, Austria) [37].

#### Interim analysis

There are no plans for any interim analyses. All analyses will be done after trial completion and the trial will terminate on a pre-determined date. While there are no prespecified stopping guidelines, all treatment failures will be monitored by the study team to ensure the safety of children exposed to TB receiving care from health extension workers (HEWs) in Ethiopia’s intervention arm.

## Discussion

CHIP-TB will evaluate a home-based healthcare delivery model for child contact management that would support the implementation and scale-up of TPT for household child contacts.

### Strengths

The cluster-randomized trial design provides population-level evidence of the effectiveness of home-based contact management, which is best delivered at a group level. Availability of estimates of the baseline characteristics from prior work in this setting is a strength of our study design. These were used to provide reasonable sample size estimates and to conduct a covariate-constrained randomization that ensures balance between the study arms [24]. The generated potential randomization sequences showed acceptable validity which is another design strength.

This study utilizes a comprehensive mixed-methods approach to not only determine the effectiveness and cost-effectiveness of community-based contact management, but also to understand how and why the intervention is successful or not.

The pragmatic intervention was developed in partnership with the local TB and community health programs, health facilities, providers, and caregivers to ensure this healthcare delivery model could continue after the study is completed. In Ethiopia, this package of care has been integrated into existing community-based services for malaria, malnutrition, pneumonia, and other important cause of under-5 mortality. The expertise, training, and experience of the staff working on this study from within their national health systems is a significant strength of this study.

The two high-burden countries have differing health system organization requiring tailored interventions for each setting. The two settings are separately powered to allow us to understand the effectiveness and cost-effectiveness for these two countries and health systems. Ultimately the differences between the two countries may enhance the generalizability of our findings given the differing implementation of home-based services in the two settings.

Enrolling child contacts 5 to 14 years of age in Ethiopia will allow us to understand the effectiveness of this intervention for school-aged children to whom the TPT recommendation has only recently been extended. Because this study was funded by IMPAACT4TB, we will also be able to enhance our knowledge of the safety and treatment outcomes for 3HP in children 2 years and older.

### Limitations

Prior to study initiation, routine medical records in both South Africa and Ethiopia lacked sufficient detail to be able to collect the necessary data for all primary and secondary outcomes. Study-wide implementation of a child contact management form was needed across intervention and control clinics. Data points were pragmatic and consistent with best practices from the WHO TPT handbook [19]. Healthcare workers will be relied upon to collect these data. We will minimize missing and erroneous data by conducing pre-trial data collection training and by regularly performing data quality checks at each facility and reinforcing training for healthcare workers as needed.

Training of healthcare workers, reinforcement of data collection processes, and the presence of research staff may increase the quality of child contact management in standard of care facilities, which may therefore limit the effect size of the intervention.

In South Africa, research nurses will be hired to conduct the household visits due to overall staff and OTL shortages. This may impact the generalizability of the study results in South Africa. However, this will not be the case in Ethiopia where the health extension workers, employed by the health system, will be implementing the study.

We expect that between-cluster contamination will be limited by country guidelines that require TB patients to seek care at the clinic to which their home is assigned to ensure the clinic’s ability to provide directly-observed therapy for the TB index patient. This, however, is variably enforced by clinics in South Africa where anyone who lives within the catchment area of the study will be treated based on where the index patient sought care. This information will be collected and, if relevant, may be used in sensitivity analyses.

### Challenges

Implementation research is pragmatic by definition and heavily influenced by changes in policy and environmental factors out of the study’s control. The protocol was written such that all medical decision-making, regimen use, and entry criteria were adaptable based on changes in country guidance to limit protocol amendments and satisfy concerns by ethics committees. Environmental factors were collected at the clinic level monthly so as to follow any changes in other projects or programming that could bias the outcome, including stockouts or TPT, culture bottles, or Xpert cartridges that could reduce TPT initiation and bacteriologic confirmation among index patients. In Ethiopia, all clinics were part of the IMPAACT4TB project, and drug delivery will be closely monitored by the parent study. In South Africa, drug stock will be monitored by the study itself and relies on the TB program for drug procurement. Finally, staff shortages including TB focal persons at the clinic and community health teams will be closely monitored. The study will not be able to control the replacement of clinic-based staff. This can influence outcomes and could explain poor performance among clinics/clusters.

The COVID-19 pandemic presented numerous challenges to the coordination of this international study as a result of travel restrictions and work delays; the additional strain on the healthcare workers and healthcare systems in South Africa and Ethiopia also impacted the study implementation. The civil conflict in Ethiopia presents additional safety and logistical concerns.

### Trial status

The study initiated enrollment in Ethiopia in September 2021 and in South Africa in March 2022. Enrollment is expected to be completed in March 2023. The current study protocol is version 4.0 dated 27 April 2021.

## Supplementary Information

**Additional file 1: Supplementary File 1.** De-identified list of participating clusters in Ethiopia and South Africa.

**Additional file 2: Supplementary File 2a.** Informed consent forms for study participants in Ethiopia.

**Additional file 3: Supplementary File 2b.** Informed consent forms for study participants in South Africa.

**Additional file 4: Supplementary File 3.** SPIRIT checklist.

## Data Availability

Data will be available upon request from the research team.
