# Supplementary material for: Pragmatic cluster-randomized trial of home-based preventive treatment for TB in Ethiopia and South Africa (CHIP-TB)
Source: Trials. 2023 Jul 25;24:475. doi: 10.1186/s13063-023-07514-7 (PMC10367260; doi:10.1186/s13063-023-07514-7)
Supplement: Supplementary file 2 — Additional file 2: Supplementary File 2a. Informed consent forms for study participants in Ethiopia. [file 13063_2023_7514_MOESM2_ESM.pdf]

INFORMATION LEAFLET AND ASSENT FORM FOR CHILD CONTACT: 12 - 14 YEARS

CHILD CONTACT: CONTROL CLINIC

PROTOCOL: A pragmatic cluster-randomized trial of community-based contact investigation and initiation of TB preventive therapy in South Africa and Ethiopia

SHORT TITLE: Home Based TPT

VERSION: Protocol Version 4.0, dated 27 April 2021
Assent Form Version 4.0, dated 27 April 2021

PRINCIPAL INVESTIGATOR: Dr Ahmed Bedru

TELEPHONE: +251 911 405 405

Good day, I am_____________, a nurse/community health worker from the _____ clinic. I would like to invite you to consider participating in a research study. We want to see if you would agree to be in a research study.

This research study will determine if the clinic or the home is the best place for children exposed to TB to get checked for and given medicine to prevent TB. All of the care your child will receive is by national guideline. You and your child do not need to do anything extra to be a part of this study. If you do not want to be a part of this study, you can still get all the care your child needs at the clinic.

The main risk of being in this study is loss in confidentiality. We have many preventive measures in place to make sure this does not happen.

Someone in your house has TB. If someone has TB, the clinic should send health workers to your home to see if anybody else in your home also has TB, especially the other children.

This study is looking to see if these home visits work well. Children can get tuberculosis easily. TB is spread by people coughing and others close by breathing in that air and children are very likely to be infected with TB. People who are living with HIV are also very likely to be infected with TB. Sometimes, children may need HIV testing.

Every child living with someone diagnosed with tuberculosis in this clinic will be asked if they want to join the study. If you agree to be in the study, we will:

1. Ask your caretaker for their permission for you to join the study.
2. Ask to look at your clinic chart until you complete your treatment.

CHILD CONTACT: CONTROL CLINIC

It is possible that someone from the study will be present while you are checked for TB. They want to know how much time the nurse spends with you. This will help us understand how much the home visits cost. The nurse will ask you if you want the person from the study to leave. You can say whether you want them to stay or leave.

By taking part you may help other kids with TB as the researchers will learn something from you.

You do not have to be in the study. If you want, you can tell us any time that you want to leave.

People in the study will be able to see your information. We will try to make sure that your name is not on any documents or emails that leave the clinic.

If you feel the study staff has not treated you properly, you can get hold of Dr Ahmed Bedru at the KNCV Tuberculosis Foundation, Ethiopia – Tel: +251 911 405 405.

This study has been approved by the Oromia Regional Health Bureau IRB and written approval has been granted by that committee. This study has also been approved by the Johns Hopkins IRB. Johns Hopkins will help KNCV monitor the study and analyze the data.

The study has been structured in accordance with the Declaration of Helsinki (last updated: October 2013) and National Ethical Guidelines which deals with the recommendations guiding doctors in biomedical research involving human participants. I can obtain a copy for you if you wish to review it.

If you want any information regarding your rights as a participant, or have complaints regarding this study, you may contact:

Oromia:
Gemechu Shumi: Chairperson
Oromia Regional Health Bureau Public Emergency and Health Research Directorate IRB
Telephone number: +251922279214

United States of America:
Johns Hopkins Medical – IRB X
The Johns Hopkins University
Institutional Review Board
Telephone number: (001) 410 955 3008

Do you have any questions before you decide to be in this study? If so, please ask me.

INFORMATION LEAFLET AND ASSENT FORM FOR CHILD CONTACT: 12 - 14 YEARS

CHILD CONTACT: CONTROL CLINIC

SIGNATURE PAGE

STATEMENT OF ASSENT

Before you sign this assent form, make sure of the following:

- You have read this assent form, or someone has read it to you.
- This study has been explained to you and had your questions answered.
- You understand you can ask more questions at any time.
- You have understood everything that has been explained to you and you assent to participate in this research study.
- You understand that you can without prejudice withdraw your assent at any time. If that happened, any data collected about me for the purposes of the study would be destroyed, unless I give assent for it to be retained.

Name of Parent / Legal Guardian ______________________________

Do you agree for your child to be in this study? YES_______ NO_______ (Please initial)

Name of child (if ≥12 years): ______________________________

Do you agree to be in this study? YES_______ NO_______

______________________	______________________	___________	☐☐☐☐
**Participant Name and Second/middle name (Print)**	**Participant Signature/ Thumbprint**	**Date (dd/mmm/yyyy)**	**TIME**

______________________	______________________	___________	☐☐☐☐
**Clinic Staff conducting assent Name and Second/middle name (Print)**	**Clinic Staff conducting assent Signature/Thumbprint**	**Date (dd/mmm/yyyy)**	**TIME**

**For participants who are unable to read, also complete the signature block below:*

______________________	______________________	___________	☐☐☐☐
**Witness' Name and Second/ middle name (Print)**	**Witness' Signature**	**Date (dd/mmm/yyyy)**	**TIME**

**Witness is impartial and was present for the entire assent process.*

Retain one original Assent Form on file. Offer participant the other original signed assent. Place a copy in medical records if applicable.

INFORMATION LEAFLET AND ASSENT FORM FOR CHILD CONTACT: 12 - 14 YEARS

CHILD CONTACT: INTERVENTION CLINIC

PROTOCOL: A pragmatic cluster-randomized trial of community-based contact investigation and initiation of TB preventive therapy in South Africa and Ethiopia

SHORT TITLE: Home Based TPT

VERSION: Protocol Version 4.0, dated 27 April 2021
Assent Form Version 4.0, dated 27 April 2021

PRINCIPAL INVESTIGATOR: Dr Ahmed Bedru

TELEPHONE: +251 911 405 405

Good day, I am____________, a nurse/community health worker from the _____ clinic. I would like to invite you to consider participating in a research study. We want to see if you would agree to be in a research study.

This research study will determine if the clinic or the home is the best place for children exposed to TB to get checked for and given medicine to prevent TB. All of the care your child will receive is by national guideline. You and your child do not need to do anything extra to be a part of this study. If you do not want to be a part of this study, you can still get all the care your child needs at the clinic.

The main risk of being in this study is loss in confidentiality. We have many preventive measures in place to make sure this does not happen.

Someone in your house has TB. If someone has TB, the clinic should send health workers to your home to see if anybody else in your home also has TB, especially the other children.

This study is looking to see if these home visits work well. Children can get TB easily. TB is spread by people coughing and others close by breathing in that air and children are very likely to be infected with TB. People who are living with HIV are also very likely to be infected with TB. Sometimes, children may need HIV testing.

Every child living with someone diagnosed with tuberculosis in this clinic will be asked if they want to join the study. If you agree to be in the study, we will:

1. Ask your caretaker for their permission for you to join the study.

2. Check you to see if you have TB or HIV. If you have TB, we will ask your caretaker to bring you to the clinic for treatment. If you do not have TB, we will offer medicine to keep you from getting TB that you can take here in your home.
3. Ask to look at your clinic chart until you complete your treatment.

It is possible that someone from the study will be present while you are checked for TB. They want to know how much time the nurse spends with you. This will help us understand how much the home visits cost. The nurse will ask you if you want the person from the study to leave. You can say whether you want them to stay or leave.

By taking part you may help other kids with TB as the researchers will learn something from you.

You do not have to be in the study. If you want, you can tell us any time that you want to leave.

People in the study will be able to see your information. We will try to make sure that your name is not on any documents or emails that leave the clinic.

If you feel the study staff has not treated you properly, you can get hold of Dr Ahmed Bedru at the KNCV Tuberculosis Foundation, Ethiopia – Tel: +251 911 405 405.

This study has been approved by the Oromia Regional Health Bureau IRB and written approval has been granted by that committee. This study has also been approved by the Johns Hopkins IRB. Johns Hopkins will help KNCV monitor the study and analyze the data.

The study has been structured in accordance with the Declaration of Helsinki (last updated: October 2013) and National Ethical Guidelines which deals with the recommendations guiding doctors in biomedical research involving human participants. I can obtain a copy for you if you wish to review it.

If you want any information regarding your rights as a participant, or have complaints regarding this study, you may contact:

<u>Oromia:</u>
Gemechu Shumi: Chairperson
Oromia Regional Health Bureau Public Emergency and Health Research Directorate IRB
Telephone number: +251922279214

<u>United States of America:</u>
Johns Hopkins Medical – IRB X
The Johns Hopkins University
Institutional Review Board
Telephone number: (001) 410 955 3008

Do you have any questions before you decide to be in this study? If so, please ask me.

INFORMATION LEAFLET AND ASSENT FORM FOR CHILD CONTACT: 12 - 14 YEARS

CHILD CONTACT: INTERVENTION CLINIC

SIGNATURE PAGE

STATEMENT OF ASSENT

Before you sign this assent form, make sure of the following:

- You have read this assent form, or someone has read it to you.
- This study has been explained to you and had your questions answered.
- You understand you can ask more questions at any time.
- You have understood everything that has been explained to you and you assent to participate in this research study.
- You understand that you can without prejudice withdraw your assent at any time. If that happened, any data collected about me for the purposes of the study would be destroyed, unless I give assent for it to be retained.

Name of Parent / Legal Guardian ______________________________

Do you agree for your child to be in this study? YES_______ NO_______ (Please initial)

Name of child (if ≥12 years): ____________________________

Do you agree to be in this study? YES_______ NO_______

Participant Name and Second/middle name (Print)	Participant Signature/ Thumbprint	Date (dd/mmm/yyyy)	TIME

Clinic Staff conducting assent Name and Second/middle name (Print)	Clinic Staff conducting assent Signature/Thumbprint	Date (dd/mmm/yyyy)	TIME

**For participants who are unable to read, also complete the signature block below:*

Witness' Name and Second /middle name (Print)	Witness' Signature	Date (dd/mmm/yyyy)	TIME

**Witness is impartial and was present for the entire assent process.*

Retain one original Assent Form on file. Offer participant the other original signed assent. Place a copy in medical records if applicable.

UNKAA ODEEFFANNOO FI HEEYYAMAMUMMAA
DEEBII KENNAA

<u>HIRMAATOTTA GAHEESSA</u>: TO'ANA KILINIKA

SANSAKAA: Garee praagraamaatikii yaali raandamaayizidii qorrannoo ittissa dhukkuba sombaa haawaassa gidduu gala goodhatee afrikaa kibbaatti fi Itoophiyaa.

Mata duree gabaabaa: Tajaajiilawwan TPT mana keessatti kennamu

---

KAN FOOYYA'E: Sansakaa fooyya'e 4.0, gaafa gguyyaa Ebla 19 bara 2013 A.L.I
Unkaa heeyyamamummaa hirmaannaa 4.0, Gaafa guyyaa Ebla 19 bara 2013 A.L.I

QORATAA MUUMMEE: Dr. Ahimad Badruu

Bilbiila: +251 911 405 405

Ani maqaan kiyya ________ ogummaan kiyya Narsii/gorsa kennaa yemmuu ta'uu sababa qoroannoo kanaatiif kiliinika ______, jedhamu irraa dhufeen jira. Haaluma kanaan, qorannoo kana ogeessoota qorannoo dhukkuba sombaa /TB kiliinika kanaa wajjin hojjechaa jirra. Kanaafuu qorannoo kana irratti hirmaachuuf heeyyamamummaa keessan akka nuuf ibsitan isiin gaafanna.

Qorannoon kun daa'ima dhukkuba sombaatif/TB saaxilmanif qoricha ittisa TB lachuf iddon mijaa'aan sadarka manarattimoo kilinikarrati gaaridha waan jedhu murtesa. Tajaajili daa'imni argatuu qajelcha biyolessarratti hunda'a yemuu ta'u, qoranno kanarratti hirmachuf wanti dabalataan daamni fi isinnis gootan hinjiru. Qoranno kanarratti hirmaachu yoo hin barbaaneelee tajaajilli kilinika kana keessattii daa'imaaf barbaachisuu ni keenamaaf.

Qoranoo kanaratti hirmaachu kessaniff balaan ijoon issinira gahu danda'uu iccitii qabuu dhissudha kannaaf imoo safari heduu bakka kenye jirra.

UNKAA ODEEFFANNOO FI HEEYYAMAMUMMAA
DEEBII KENNAA

HIRMAATOTTA GAHEESSA: TO'ANA KILINIKA

Dhukkuba sombaa /TB tiin qabamuun keessan qorannoodhaan waan hubatameef, qorannoo kana irratti akka hirmaattan isiin afera. Dhukkubni sombaa/TB qileensa/afuura waliitti baafachudhaan nama tokko irraa gara nama adda birootti ni dadarba. Haaluma kanaan, daa'imman maatiin /miseensi maatii isaanii dhukkuba sombaa/TB tiin qabame carraan dhukkuba kanaan qabamu isaanii ol'aanaadha. Kanuma irra ka'udhaan, ogeessoonni fayyaa maatii tokkoo keessaa namni tokko dhukkuba sombaa/TB tiin qabamu isaa yemmuu qorannoodhaan irra gahan gara daa'imman ykn miseensota maatii adda birootti kan darban.

Kaayyoon qorannoo kanaa haala kamiin daa'imman dhukkuba sombaa /TB tiin qabamaniif qoriicha isaan barbaachisu rabsuun akka danda'amu irratti kan xiyyeeffatedha. Qorannoon kun daa'immaniif mana keessatti ykn kiliinika keessatti godhamu ni danda'a. Qorannoo kana keessatti, daa'imman muraasaaf kiliinika keessatti qorannoon godhame qoriichi kan kennamuuf yemmuu ta'uu daa'imman hafaniif immoo mana keessatti qorannoon godhame qoriichi kan kennamuuf ni ta'a.

Qorannoo kana keessatti hirmaachuun fedhii fi heeyyamamummaa keessan irratti kan hundaa'u ni ta'a. Kanaafuu, qorannoo kana keessatti hirmaachuuf dirqamni hin jiru. Namni qorannoo kana keessatti hirmaachuu fedhii /heeyyamamummaa hinqabneef tajaajiilli idilee kiliinika keessatti kennamu hundinu ni kennama. Itti dabalataanis, qorannoo kana keessatti hirmaachuu ergi jalqabdanii booda yeroo kamiiyyuutti addaan kutuu ykn qorannicha keessa bahuuf mirgii keessan kan eegamedha.

Sansakaa: Tajaajiilawwan TPT mana keessatti kennamu 4.0, gaafa guyyaa Ebla 19 bara 2013 A.L.I

INGILIIFFAA: Unkaa odeeffannoo fi heeyyamamummaa deebii kennaa -dhukkubsattoota gaheessa: To'ana kilinika 4.0, Gaafa guyyaa Ebla 19 bara 2013 A.L.I
qorataa Muummee: Dr. Ahimad Badruu
Kan mirkaneesse: Biiroo Eegumsa Fayyaa Mootummaa Naannoo Oromiyaa, Qorannoo Dhukkuba Tasaa Hawaasaa – IRB
Guyyaa itti mirkanaa'e: 13/09/2021

UNKAA ODEEFFANNOO FI HEEYYAMAMUMMAA
DEEBII KENNAA

HIRMAATOTTA GAHEESSA: TO'ANA KILINIKA

Qorannoo kana keessatti hirmaachuuf heeyyamamaa yoo taatan, odeeffannoowwan kessan galmee kiliinika kessatti argamu xinxaludhaan gata fulduratti dhukkubsatoota dhukkuba gosa walfakkaatuun qabamaniitiif yaalii ykn wal'ansa gochuuf ni fayyadamna. Hanga wal'ansa dhukkuba sombaa/TB xumutaniitti galmee kessan kiliinika keessatti argamu ilaaluu ni dandeessu.

Odeeffannoo daa'imman keessani galmee kiliinika keessatti galmeeffaman hundaa iccitaan ni eegna. Odeeffannoo kana ilaaluuf ykn argu kan danda'u ogeessoota kiliinika qofa ni ta'a

Daa'immni keessan qorannoo kana irratti hirmaachuuf wanti dabalataa isiin irraa barbaadamu hin jiru. Qorannoo kana keessatti hirmaachuun keessan haala eegumsa fayyaa daa'immanii fooyyeessuu fi qorannoo dhukkuba sombaa /TB irratti godhamaa jiru jajabeessuuf gumaacha ol'aanaa ni qaba.

Ogeessi qorannoo kana gaggeessu sirritti isiin hin keesumsisiin yoo hafe qorataa muummee dhaabbata qorannoo dhukkuba sombaa /TB/ KNCV Tuberculosis Foundation, Itiyoophiyaa – Lakk.: +251 911 405 405.kan ta'an Dr. Ahimad Badruutti bilbiilu ni dandeessu.

Qorannoon kun Biiroo Eegumsa Fayyaa Mootummaa Naannoo Oromiyaa, Qorannoo Dhukkuba Tasaa Hawaasaa tiin kan mirkanaa'ee fi koree kana irraa xalayaa deeggarsaa /mirkana argatee jira. Qorannoon kun dhaabata Joonhopkinssittiin kan mirkanaa'eedha. Akkasumas KNCViin hordoffi fi xintala daataarrati gargaarsa jonhopkinsirra ni argata .
Caassaan qoorannoo kanaa walfakkenya labssii heeliisinkii fi qajeelcha naamusa biyaaleessaa dooctaroota baayoomeedikaala marii waa'ee hirmaatotta ilmaan namarrattii godhanniif yaada kennuu. Haftee issaa laaluu ykn duubiisuu yoo

barbaadan issinitti laachuun ni danda'aama.

Akka hirmaataa tokkootti gaaffii mirgaa yoo qabaattan ykn qorannoo kana ilaalchisee komii yoo qabatan teessoo armaan gaditiin nu argachuu dandeessu.

<u>Oromiyaa:</u>
Gamachuu Shumii: Dura taa'aa
Biiroo Eegumsa Fayyaa Mootummaa Naannoo Oromiyaa, Qorannoo Dhukkuba Tasaa Hawaasaa – IRB
Lakk. Bilbiila: +251922279214

<u>Yunaayitid Steetis Of Ameerikaa:</u>
Johns Hopkins Medical – IRB X
The Johns Hopkins University
Institutional Review Board
Lakk. Bilbiila: (001) 410 955 3008

Qorannoo kana irratti hirmaachuuf osoo hin murteessiin dura gaaffii dabalataa qabduu? Gaaffii yoo qabaattan na gaafachuu dandeessu .

UNKAA ODEEFFANNOO FI HEEYYAMAMUMMAA
DEEBII KENNAA

HIRMAATOTTA GAHEESSA: TO'ANA KILINIKA

FUULA MALLATTOO

JECHA HEEYYAMAMUMMAA

Qorannoo kana irratti hirmaachuuf heeyyamammaa ta'uu keessanmirkaneessuuf unkaa kana irratti osoo hin mallatteessin dura, dhimmoota armaan gadi sirritti hubadhaa

- Unkaa heeyyamamummaa kana sirritti dubbiseen jira ykn namni naaf dubbisee jira.
- Kaayyoon qorannoo kana isiniif ibsame gaafii keessan hundaaf deebii gahaan isiniif kenname jira.
- Ammas irra deebiidhaan gaaffii dabalataa yoo qabaattan, gaaffii keessan dhiheessuuf mirgi keessan kan eegame ta'uu hubatanii jirtu.
- Kaayyoo fi barbaachisummaan qorannoo kana sirritti isiniif ergi ibsamee booda qorannoo kana irratti hirmaachuuf heeyyamamaa taatanii jirtu.
- Yeroo turtii gaaffii fi deebii kanaa keessatti yeroo barbaadan kamiiyyuutti deebii kennu dhiisuu ykn addaan kutu akka dandeessan hubatanii jirtu.

Maqaa hirmata ______________________

______________________	______________________	____________				
**Maqaa guutuu hirmaataa/ Deebii kennaa(barreessi)**	**Mallattoo hirmaataa/ Deebii kennaa**	**Guyyaa**	**Sa'aatii**			

______________________	______________________	____________				
**Maqaa guutuu qorataa/hojjetaa Kiliinikaa (Barreessi)**	**Mallattoo qorataa /hojjetaa kiliinikaa**	**Guyyaa (GG/ jjj/bbbb)**	**Sa'aatii**			

**Hirmaataan/ deebii kennaan dubbisuu hin danda'u yoo ta'e, gabateen armaan gadi haa guutamu :*

Sansakaa: Tajaajiilawwan TPT mana keessatti kennamu 4.0, gaafa guyyaa Ebla 19 bara 2013 A.L.I

INGILIIFFAA: Unkaa odeeffannoo fi heeyyamamummaa deebii kennaa -dhukkubsattoota gaheessa: To'ana kilinika 4.0, Gaafa guyyaa Ebla 19 bara 2013 A.L.I
qorataa Muummee: Dr. Ahimad Badruu
Kan mirkaneesse: Biiroo Eegumsa Fayyaa Mootummaa Naannoo Oromiyaa, Qorannoo Dhukkuba Tasaa Hawaasaa – IRB
Guyyaa itti mirkanaa'e: 13/09/2021

UNKAA ODEEFFANNOO FI HEEYYAMAMUMMAA
DEEBII KENNAA

HIRMAATOTTA GAHEESSA: TO'ANA KILINIKA

Maqaa guutuu ragaa (barreessi)	Mallattoo ragaa	Guyyaa (gg/jjj/bbbb)	Sa'aatii	

**Ragaan heeyyamamummaa deebii kennaa irratti dhibbaa uumuu hin danda'u.*

Orjinaala/muummee unkaa kanaa galmee/faayilii wajjin walqabsisudhaan Orjinaala/muummee tokko deebii kennaa/hirmaataadhaaf kennaa. Yoo barbaachisaa ta'e waraabbii/koppii dabalataa galmee meedikaalaa dhukkubsataa wajjin waliin qabsisi

Sansakaa: Tajaajiilawwan TPT mana keessatti kennamu 4.0, gaafa guyyaa Ebla 19 bara 2013 A.L.I

INGILIIFFAA: Unkaa odeeffannoo fi heeyyamamummaa deebii kennaa -dhukkubsattoota gaheessa: To'ana kilinika 4.0, Gaafa guyyaa Ebla 19 bara 2013 A.L.I
qorataa Muummee: Dr. Ahimad Badruu
Kan mirkaneesse: Biiroo Eegumsa Fayyaa Mootummaa Naannoo Oromiyaa, Qorannoo Dhukkuba Tasaa Hawaasaa – IRB
Guyyaa itti mirkanaa'e: 13/09/2021

PARTICIPANT INFORMATION LEAFLET AND INFORMED CONSENT FORM

ADULT INDEX PARTICIPANT: CONTROL CLINIC

PROTOCOL: A pragmatic cluster-randomized trial of community-based contact investigation and initiation of TB preventive therapy in South Africa and Ethiopia

SHORT TITLE: Home Based TPT

VERSION: Protocol Version 4.0, dated 27 April 2021
Informed Consent Form Version 4.0, dated 27 April 2021

PRINCIPAL INVESTIGATOR: Dr Ahmed Bedru

TELEPHONE: +251 911 405 405

Good day, I am_____________, a counselor /nurse from the ______ clinic. I would like to invite you to consider participating in a research study. We are working together with the clinic staff here doing research about TB.

This research study will determine if the clinic or the home is the best place for children exposed to TB to get checked for and given medicine to prevent TB. All of the care your child will receive is by national guideline. You and your child do not need to do anything extra to be a part of this study If you do not want to be a part of this study, you can still get all the care your child needs at the clinic.

The main risk of being in this study is loss in confidentiality. We have many preventive measures in place to make sure this does not happen.

I am inviting you to be part of this study because you have been diagnosed with TB. TB is spread by people coughing and others close by breathing in the air. Children who live in a house with someone who has TB are very likely to be infected with TB. That is why when someone has TB, the clinic sends someone to the house to check the other family members and see if they might have TB.

Our study is looking at how to get children a medicine that they need to keep from getting TB. Children can either be checked at the clinic or in the home. In this study, some children will be checked and given medicine at the clinic and some will be checked and given medicine in the home. People who are living with HIV are also very likely to be infected with TB. Sometimes, children may need HIV testing. Children at this clinic will be checked and given medicine at the clinic.

Being part of this study is your choice. You do not have to agree. You will still be able to get proper treatment at the clinic even if you do not want to be in this study. Also, if you agree and then change your mind, you can stop being in the study at any time.

If you agree to be part of this study, we will use the information in your clinic records to see how well the care given in the clinic is works for patients like you. We will ask to look at your clinic records until you complete your treatment for TB.

We will keep the information from your clinic chart confidential. Only the study and clinic staff will know this information. We will not disclose your TB status to your household members without your permission.

You do not need to do anything extra to be part of this research study. Through this research, we hope to improve the TB services the community receives.

If you feel the study staff has not treated you properly, you can get hold of Dr Ahmed Bedru at the KNCV Tuberculosis Foundation, Ethiopia – Tel: +251 911 405 405.

This study has been approved by the Oromia Regional Health Bureau IRB and written approval has been granted by that committee. This study has also been approved by the Johns Hopkins IRB. Johns Hopkins will help KNCV monitor the study and analyze the data.

The study has been structured in accordance with the Declaration of Helsinki (last updated: October 2013) and National Ethical Guidelines which deals with the recommendations guiding doctors in biomedical research involving human participants. I can obtain a copy for you if you wish to review it.

If you want any information regarding your rights as a participant, or have complaints regarding this study, you may contact:

Oromia:
Gemechu Shumi: Chairperson
Oromia Regional Health Bureau Public Emergency and Health Research Directorate IRB
Telephone number: +251922279214

United States of America:
Johns Hopkins Medical – IRB X
The Johns Hopkins University
Institutional Review Board
Telephone number: (001) 410 955 3008

Do you have any questions or concerns before you make a decision about being in this study? If so, please ask me.

PARTICIPANT INFORMATION LEAFLET AND INFORMED CONSENT FORM

ADULT INDEX PARTICIPANT: CONTROL CLINIC

SIGNATURE PAGE

STATEMENT OF CONSENT

Before you sign this consent form, make sure of the following:

- You have read this informed consent form, or someone has read it to you.
- This study has been explained to you and had your questions answered.
- You understand you can ask more questions at any time.
- You have understood everything that has been explained to you and you consent to participate in this research study.
- You understand that you can without prejudice withdraw your consent at any time. If that happened, any data collected about me for the purposes of the study would be destroyed, unless I give consent for it to be retained.

Name of Participant: ___________________

___________________	___________________	___________________	[ ][ ][ ][ ]
**Participant Name and Second/middle name (Print)**	**Participant Signature / Thumbprint**	**Date** TIME **(dd/mmm/yyyy)**	

___________________	___________________	___________________	[ ][ ][ ][ ]
**Clinic Staff conducting consent discussion Name and Second/middle name (Print)**	**Clinic Staff Signature**	**Date (dd/mmm/yyyy)**	TIME

For participants who are unable to read, also complete the signature block below:

___________________	___________________	___________________	[ ][ ][ ][ ]
***Witness' Name and Second/middle name (Print)**	**Witness' Signature**	**Date (dd/mmm/yyyy)**	TIME

**Witness is impartial and was present for the entire consent process.*

Retain one original Informed Consent Form on file. Offer participant the other original signed consent. Place a copy in medical records if applicable.

UNKAA ODEEFFANNOO FI HEEYYAMAMUMMAA
DEEBII KENNAA

HIRMAATOOTA GAHEESSA: KILINIKA YAALIIN/WAL'ANSII ITTI KENNAMU

SANSAKAA: Garee praagraamaatikii yaali raandamaayizidii qorrannoo ittissa dhukkuba sombaa haawaassa gidduu gala goodhatee afrikaa kibbaatti fi Itoophiyaa

Mata duree gabaabaa: Tajaajiilawwan TPT mana keessatti kennamu

---

KAN FOOYYA'E: Sansakaa fooyya'e 4.0, gaafa gguyyaa Ebla 19 bara 2013 A.L.I

Unkaa heeyyamamummaa hirmaannaa 4.0, Gaafa guyyaa Ebla 19 bara 2013 A.L.I

QORATAA MUUMMEE: Dr. Ahimad Badruu

Bilbiila: +251 911 405 405

Ani maqaan kiyya ________ ogummaan kiyya Narsii/gorsa kennaa yemmuu ta'uu sababa qoroannoo kanaatiif kiliinika ______, jedhamu irraa dhufeen jira. Haaluma kanaan, qorannoo kana ogeessoota qorannoo dhukkuba sombaa /TB kiliinika kanaa wajjin hojjechaa jirra. Kanaafuu qorannoo kana irratti hirmaachuuf heeyyamamummaa keessan akka nuuf ibsitan isiin gaafanna.

Qorannoon kun daa'ima dhukkuba sombaatif/TB saaxilmanif qoricha ittisa TB lachuf iddon mijaa'aan sadarka manarattimoo kilinikarrati gaaridha waan jedhu murtesa. Tajaajili daa'imni argatuu qajelcha biyolessarratti hunda'a yemuu ta'u, qoranno kanarratti hirmachuf wanti dabalataan daamni fi isinnis gootan hinjiru. Qorannoo kanaratti hirmachu baatanilee tajaajili barbaachissaan daa'imni kilinikaraa argatchu qabuu ni laatamaaf.

Qoranoo kanaratti hirmaachu kessaniff balaan ijoon issinira gahu danda'uu iccitii qabuu dhissudha kannaaf imoo safari heduu bakka kenye jirra.

Dhukkuba sombaa /TB tiin qabamuun keessan qorannoodhaan waan

hubatameef, qorannoo kana irratti akka hirmaattan isiin afera. Dhukkubni sombaa/TB qileensa/afuura waliitti baafachudhaan nama tokko irraa gara nama adda birootti ni dadarba. Haaluma kanaan, daa'imman maatiin /miseensi maatii isaanii dhukkuba sombaa/TB tiin qabame carraan dhukkuba kanaan qabamu isaanii ol'aanaadha. Kanuma irra ka'udhaan, ogeessoonni fayyaa maatii tokkoo keessaa namni tokko dhukkuba sombaa/TB tiin qabamu isaa yemmuu qorannoodhaan irra gahan gara daa'imman ykn miseensota maatii adda birootti kan darban.

Kaayyoon qorannoo kanaa haala kamiin daa'imman dhukkuba sombaa /TB tiin qabamaniif qoriicha isaan barbaachisu rabsuun akka danda'amu irratti kan xiyyeeffatedha. Qorannoon kun daa'immaniif mana keessatti ykn kiliinika keessatti godhamu ni danda'a. Qorannoo kana keessatti, daa'imman muraasaaf kiliinika keessatti qorannoon godhame qoriichi kan kennamuuf yemmuu ta'uu daa'imman hafaniif immoo mana keessatti qorannoon godhame qoriichi kan kennamuuf ni ta'a.

Qorannoo kana keessatti hirmaachuun fedhii fi heeyyamamummaa keessan irratti kan hundaa'u ni ta'a. Kanaafuu, qorannoo kana keessatti hirmaachuuf dirqamni hin jiru. Namni qorannoo kana keessatti hirmaachuu fedhii /heeyyamamummaa hinqabneef tajaajiilli idilee kiliinika keessatti kennamu hundinu ni kennama. Itti dabalataanis, qorannoo kana keessatti hirmaachuu ergi jalqabdanii booda yeroo kamiiyyuutti addaan kutuu ykn qorannicha keessa bahuuf mirgii keessan kan eegamedha.

Qorannoo kana keessatti hirmaachuuf heeyyamamaa yoo taatan ykn fedhii kan qabdan yoo ta'e.

UNKAA ODEEFFANNOO FI HEEYYAMAMUMMAA

DEEBII KENNAA

HIRMAATOOTA GAHEESSA: KILINIKA YAALIIN/WAL'ANSII ITTI KENNAMU

1. Ogeessoota kiliinika kanaa wajjin ta'uudhaan gara mana keessani dhufnee ni daawwana. Ogeessoonni kiliinikicha daa'imman mana keessan keessatti argaman hundaaf qorannoo dhukkuba sombaa/TB gochuudhaan daa'imman kana irratti mallattoo dhukkuba sombaa/TB yoo argan daa'imman kanaaf qoriicha dhukkuba sombaa ni kennu. Daa'imman keessan irratti mallattoon dhukkuba sombaa /TB kan mul'atu yoo ta'e, guddistuun /kuninsituun daa'ima kanaa daa'immicha gara kiliinikaatti fidudhaan qorannoon dhukkuba sombaa/TB akka godhamuuf gorsa ni keniina. Daa'imman kanaaf kiliinika keessatti qorannoon osoo hin godhamiin dura heyyamamummaa maatii ykn guddistuu daa'immimcha ni gaafana. Kanaafuu heeyyamamummaa maatii ykn guddistuu malee daa'ima kamiif iyyuu qorannoon dhukkuba sombaa hin godhamu.
2. Milkaa'ina wal'ansa daa'ima keessaniif mana keessatti godhamaa jiru hubachuuf odeeffannoowwan daa'immicha galmee kiliinika keessatti barreeffaman ilaaluu ni dandeessu. Kanaafuu, hanga daa'imni keessan wal'ansa dhukkuba sombaa/TB xumuruutti, galmee wal'ansa daa'ima keessanii kiliinikicha keessatti argamu ilaaluuf mirgii keessan kan eegamedha.

Odeeffannoo daa'imman keessani galmee kiliinika keessatti galmeeffaman hundaa iccitaan ni eegna. Odeeffannoo kana ilaaluuf ykn argu kan danda'u ogeessoota kiliinika qofa ni ta'a

Daa'immni keessan qorannoo kana irratti hirmaachuuf wanti dabalataa isiin irraa barbaadamu hin jiru. Qorannoo kana keessatti hirmaachuun keessan haala eegumsa fayyaa daa'immanii fooyyeessuu fi qorannoo dhukkuba sombaa /TB irratti godhamaa jiru jajabeessuuf gumaacha ol'aanaa ni qaba.

Ogeessi qorannoo kana gaggeessu sirritti isiin hin keesumsisiin yoo hafe qorataa

UNKAA ODEEFFANNOO FI HEEYYAMAMUMMAA
DEEBII KENNAA

HIRMAATOOTA GAHEESSA: KILINIKA YAALIIN/WAL'ANSII ITTI KENNAMU

muummee dhaabbata qorannoo dhukkuba sombaa /TB/ KNCV Tuberculosis Foundation, Itiyoophiyaa – Lakk.: +251 911 405 405.kan ta'an Dr. Ahimad Badruutti bilbiilu ni dandeessu.

Qorannoon kun Biiroo Eegumsa Fayyaa Mootummaa Naannoo Oromiyaa, Qorannoo Dhukkuba Tasaa Hawaasaa tiin kan mirkanaa'ee fi koree kana irraa xalayaa deeggarsaa /mirkana argatee jira. Qorannoon kun dhaabata Joonhopkinssittiin kan mirkanaa'eedha. Akkasumas KNCViin hordoffi fi xintala daataarrati gargaarsa jonhopkinsirra ni argata.
Caassaan qoorannoo kanaa walfakkenya labssii heeliisinkii fi qajeelcha naamusa biyaaleessaa dooctaroota baayoomeedikaala marii waa'ee hirmaatotta ilmaan namarrattii godhanniif yaada kennuu. Haftee issaa laaluu ykn duubiisuu yoo barbaadan issinitti laachuun ni danda'aama.

Akka hirmaataa tokkootti gaaffii mirgaa yoo qabaattan ykn qorannoo kana ilaalchisee komii yoo qabatan teessoo armaan gadiitiin nu argachuu dandeessu.

____

UNKAA ODEEFFANNOO FI HEEYYAMAMUMMAA
DEEBII KENNAA

HIRMAATOOTA GAHEESSA: KILINIKA YAALIIN/WAL'ANSII ITTI KENNAMU

Oromiyaa:

Gamachuu Shumii: Dura taa'aa

Biiroo Eegumsa Fayyaa Mootummaa Naannoo Oromiyaa, Qorannoo Dhukkuba Tasaa Hawaasaa – IRB

Lakk. Bilbiila: +251922279214

Yunaayitid Steetis Of Ameerikaa:

Johns Hopkins Medical – IRB X

The Johns Hopkins University

Institutional Review Board

Lakk. Bilbiila: (001) 410 955 3008

Qorannoo kana irratti hirmaachuuf osoo hin murteessiin dura gaaffii dabalataa qabduu? Gaaffii yoo qabaattan na gaafachuu dandeessu .

UNKAA ODEEFFANNOO FI HEEYYAMAMUMMAA

DEEBII KENNAA

HIRMAATOOTA GAHEESSA: KILINIKA YAALIIN/WAL'ANSII ITTI KENNAMU

FUULA MALLATTOO

JECHA HEEYYAMAMUMMAA

Qorannoo kana irratti hirmaachuuf heeyyamammaa ta'uu keessanmirkaneessuuf unkaa kana irratti osoo hin mallatteessin dura, dhimmoota armaan gadi sirritti hubadhaa

- Unkaa heeyyamamummaa kana sirritti dubbiseen jira ykn namni naaf dubbisee jira.
- Kaayyoon qorannoo kana isiniif ibsame gaafii keessan hundaaf deebii gahaan isiniif kenname jira.
- Ammas irra deebiidhaan gaaffii dabalataa yoo qabaattan, gaaffii keessan dhiheessuuf mirgi keessan kan eegame ta'uu hubatanii jirtu.
- Kaayyoo fi barbaachisummaan qorannoo kana sirritti isiniif ergi ibsamee booda qorannoo kana irratti hirmaachuuf heeyyamamaa taatanii jirtu.
- Yeroo turtii gaaffii fi deebii kanaa keessatti yeroo barbaadan kamiiyyuutti deebii kennu dhiisuu ykn addaan kutu akka dandeessan hubatanii jirtu.

Maqaa hirmata ______________________

Maqaa guutuu hirmaataa/ Deebii kennaa(barreessi)	Mallattoo hirmaataa/ Deebii kennaa	Guyyaa	Sa'aatii
____________	____________	______	

Maqaa guutuu qorataa (Barreessi)	Mallattoo qorataa	Guyyaa (GG/ jjj/bbbb)	Sa'aatii
____________	____________	______	

**Hirmaataan/deebii kennaan dubbisuu hin danda'u yoo ta'e, gabateen armaan gadi haa guutamu :*

UNKAA ODEEFFANNOO FI HEEYYAMAMUMMAA

DEEBII KENNAA

HIRMAATOOTA GAHEESSA: KILINIKA YAALIIN/WAL'ANSII ITTI KENNAMU

**Maqaa guutuu ragaa (barreessi)**	**Mallattoo ragaa**	**Guyyaa (gg/jjj/bbbb)**	**Sa'aatii**

**Ragaan heeyyamamummaa deebii kennaa irratti dhibbaa uumuu hin danda'u.*

Orjinaala/muummee unkaa kanaa galmee/faayilii wajjin walqabsisudhaan Orjinaala/muummee tokkoo deebii kennaa/hirmaataadhaaf kennaa. Yoo barbaachisaa ta'e waraabbii/koppii dabalataa galmee meedikaalaa dhukkubsataa wajjin waliin qabsisi

Sansakaa: Tajaajiilawwan TPT mana keessatti kennamu 4.0, gaafa guyyaa Ebla 19 bara 2013 A.L.I

INGILIIFFAA: Unkaa odeeffannoo fi heeyyamamummaa deebii kennaa -dhukkubsattoota gaheessa: kilinika yaaliin/wal'ansii itti kennamu 4.0, Gaafa guyyaa Ebla 19 bara 2013 A.L.I
qorataa Muummee: Dr. Ahimad Badruu
Kan mirkaneesse: Biiroo Egumsa Fayyaa Mootummaa Naannoo Oromiyaa, Qorannoo Dhukkuba Tasaa Hawaasaa – IRB
Guyyaa itti mirkanaa'e: 13/09/2021

PARTICIPANT INFORMATION LEAFLET AND INFORMED CONSENT FORM

<u>ADULT</u> INDEX PARTICIPANT: <u>INTERVENTION</u> CLINIC

PROTOCOL: A pragmatic cluster-randomized trial of community-based contact investigation and initiation of TB preventive therapy in South Africa and Ethiopia

SHORT TITLE: Home Based TPT

VERSION: Protocol Version 4.0, dated 27 April 2021
Informed Consent Form Version 4.0, dated 27 April 2021

PRINCIPAL INVESTIGATOR: Dr Ahmed Bedru

TELEPHONE: +251 911 405 405

Good day, I am____________, a counselor /nurse from the _______ clinic. I would like to invite you to consider participating in a research study. We are working together with the clinic staff here doing research about TB.

This research study will determine if the clinic or the home is the best place for children exposed to TB to get checked for and given medicine to prevent TB. All of the care your child will receive is by national guideline. You and your child do not need to do anything extra to be a part of this study. If you do not want to be a part of this study, you can still get all the care your child needs at the clinic.

The main risk of being in this study is loss in confidentiality. We have many preventive measures in place to make sure this does not happen

I am inviting you to be part of this study because you have been diagnosed with TB. TB is spread by people coughing and others close by breathing in the air. Children who live in a house with someone who has TB are very likely to be infected with TB. That is why when someone has TB, the clinic sends someone to the house to check the other family members and see if they might have TB.

Our study is looking at how to get children a medicine that they need to keep from getting TB. Children can either be checked at the clinic or in the home. In this study, some children will be checked and given medicine at the clinic and some will be checked and given medicine in the home. People who are living with HIV are also very likely to be infected with TB. Sometimes, children may need HIV testing. Children at this clinic will be checked and given medicine in the home.

Being part of this study is your choice. You do not have to agree. You will still be able to get proper treatment at the clinic even if you do not want to be in this study. Also, if you agree and then change your mind, you can stop being in the study at any time.

If you agree to be part of this study, we will:

1. Visit your home along with clinic staff who will check how you are doing. The clinic staff will also check the children who might be living in your homestead. If children do not have signs of TB, we will offer them medicine to keep them from getting TB. If children have signs of TB, we will ask their caregivers to bring them to the clinic to be checked for TB. Before checking the children, we will ask consent from the child's parent/legal guardian. We will not check the children without this consent.
2. Use the information in your clinic records to see how well the care given to these children in the home, works for household members like yours. We will ask to look at your clinic records until you complete your treatment for TB.

We will keep the information from your clinic chart confidential. Only the study and clinic staff will know this information. We will not disclose your TB status to your household members without your permission.

You do not need to do anything extra to be part of this research study. Through this research, we hope to improve the TB services the community receives.

If you feel the study staff has not treated you properly, you can get hold of Dr Ahmed Bedru at the KNCV Tuberculosis Foundation, Ethiopia – Tel: +251 911 405 405.

This study has been approved by the Oromia Regional Health Bureau IRB and written approval has been granted by that committee. This study has also been approved by the Johns Hopkins IRB. Johns Hopkins will help KNCV monitor the study and analyze the data.

The study has been structured in accordance with the Declaration of Helsinki (last updated: October 2013) and National Ethical Guidelines which deals with the recommendations guiding doctors in biomedical research involving human participants. I can obtain a copy for you if you wish to review it.

If you want any information regarding your rights as a participant, or have complaints regarding this study, you may contact:

<u>Oromia:</u>
Gemechu Shumi: Chairperson
Oromia Regional Health Bureau Public Emergency and Health Research Directorate IRB
Telephone number: +251922279214

<u>United States of America:</u>
Johns Hopkins Medical – IRB X
The Johns Hopkins University
Institutional Review Board
Telephone number: (001) 410 955 3008

Do you have any questions or concerns before you make a decision about being in this study? If so, please ask me.

PARTICIPANT INFORMATION LEAFLET AND INFORMED CONSENT FORM

ADULT INDEX PARTICIPANT: INTERVENTION CLINIC

SIGNATURE PAGE

STATEMENT OF CONSENT

Before you sign this consent form, make sure of the following:

- You have read this informed consent form, or someone has read it to you.
- This study has been explained to you and had your questions answered.
- You understand you can ask more questions at any time.
- You have understood everything that has been explained to you and you consent to participate in this research study.
- You understand that you can without prejudice withdraw your consent at any time. If that happened, any data collected about me for the purposes of the study would be destroyed, unless I give consent for it to be retained.

Name of Participant: ___________________

______________________	______________________	______________	☐☐☐☐
**Participant Name and Second/middle name (Print)**	**Participant Signature / Thumbprint**	**Date (dd/mmm/yyyy)**	**TIME**
			☐☐☐☐
______________________	______________________	______________	**TIME**
**Study Staff conducting consent discussion Name and Second/middle name (Print)**	**Study Staff Signature**	**Date (dd/mmm/yyyy)**	

**For participants who are unable to read, also complete the signature block below:*

______________________	______________________	______________	☐☐☐☐
***Witness' Name and Second/ middle name name (Print)**	**Witness' Signature**	**Date (dd/mmm/yyyy)**	**TIME**

**Witness is impartial and was present for the entire consent process.*

Retain one original Informed Consent Form on file. Offer participant the other original signed consent. Place a copy in medical records if applicable.

UNKAA HEEYYAMAMUMMAA HIRMAANNAA QORANNOO DAA'IMMAN/ IJOOLLEE UMRI: WAGGAA 12 - 14

WALIITTI DHUUFEENYA DAA'IMMANI: KILIINIKA YAALIIN/WAL'ANSII ITTI KENNAMU

SANSAKAA : Garee praagraamaatikii yaali raandamaayizidii qorrannoo ittissa dhukkuba sombaa haawaassa gidduu gala goodhatee afrikaa kibbaatti fi Itoophiyaa

Mata duree gabaabaa: Tajaajiilawwan TPT mana keessatti kennamu

KAN FOOYYA'E: Sansakaa fooyya'e 4.0, gaafa gguyyaa Ebla 19 bara 2013A.L.I

Unkaa Mirkaaneessuu heeyyamamummaa hirmaannaa 4.0, Gaafa guyyaa Ebla 19 bara 2013 A.L.I

QORATAA MUUMMEE: Dr. Ahimad Badruu

Bilbiila: +251 911 405 405

Ani maqaan kiyya ________ ogummaan kiyya Narsii/gorsa kennaa yemmuu ta'uu sababa qoroannoo kanaatiif kiliinika ______, jedhamu irraa dhufeen jira. Haaluma kanaan, qorannoo kana irratti akka hirmaattan kabajaadhaan ni gaafadha. Kanaafuu qorannoo kana irratti hirmaachuuf heeyyamamummaa keessan akka nuuf ibsitan isiin gaafanna.

Qorannoon kun daa'ima dhukkuba sombaatif/TB saaxilmanif qoricha ittisa TB lachuf iddon mijaa'aan sadarka manarattimoo kilinikarrati gaaridha waan jedhu murtesa. Tajaajili daa'imni argatuu qajelcha biyolessarratti hunda'a yemuu ta'u, qoranno kanarratti hirmachuf wanti dabalataan daamni fi isinnis gootan hinjiru. Qorannoo kanaratti hirmachu baatanilee tajaajili barbaachissaan daa'imni kilinikaraa argatchu qabuu ni laatamaaf.

WALIITTI DHUUFEENYA DAA'IMMANI: KILIINIKA YAALIIN/WAL'ANSII ITTI KENNAMU

Qoranoo kanaratti hirmaachu kessaniff balaan ijoon issinira gahu danda'uu iccitii qabuu dhissudha kannaaf imoo safari heduu bakka kenye jirra.

Maatii keessan keessaa namni tokko dhukkuba sombaa/TB tiin qabame jira. Maatii tokko keessa namni tokko dhukkuba sombaa/TB tiin yoo qabame, kiliinikichi ogeessoota fayyaa isaa gara mana keessanitti ergudhaan miseensi maatii adda biroon kessumaa iyyuu daa'imman adda biroon dhukkuba sombaa/TB tiin qabamuu isaanii ni qorata.

Kanaafuu, kaayyoon qorannoo kanaa bu'aa qabeessummaa tajaajiilawwan qorannoo fi ittiisa dhukkuba sombaa /TB keessummaayyuu daa'imman irratti kan xiyyeeffatedha. Daa'imman salphaaatti dhukkuba sombaa/TB tiin qabamuu ni danda'u. Dhukkubbni sombaa /TB afuura ykn qufaa nama dhukkubba kanaan qabameetiin gara namoota adda biroo fi daa'immanitti darbuu ni danda'a.

Namni kiliinika keenya keessatti qorannoon godhameefi dhukkuba sombaa /TB tiin qabamuun isaa mirkanaa'e hundinuu, qorannoo kana irratti hirmaachuuf fedhii yoo qabaatan ni gaafatamu. Haaluma kanaan, qorannoo kana irratti hirmaachuuf heeyyamammaa yoo taatan, gaaffiiwwan armaan gadi isiniif dhiheesina.

1. Haadha ykn abbaa ykn guddistuun keessan akka isiin qorannoo kana keessatti hirmaataniif heeyyamamummaa isaanii ni gaafanna.
2. Dhukkuba sombaa/TB tiin qabamuu keessan addaan baasuuf qorannoon isiniif godhama. Dhukkuba sombaa/TB tiin qabamuun keessan

qorannoodhaan yoo mirkanaa'e, miseensi maatii isiin wal"anu gara kiliniika keenyatti akka isiin fide dhufu gorsa ni kennina. Yoo dhukkuba sombaa/TB tiin hin qabamne ta'e immoo, qoricha ittiisa dhukkuba sombaa/TB mana keessatti akka fudhatuuf ni kennamaaf.

3. Hanga yaalii /wal'ansa keessan xumurtaniitti kaardii /galmee keessan kiliinika keenya keessatti argamu ilaaluu ni dandeessu.

Gaafa yaali Tiibii gootan narsiin sa'aattii meeqa isiiniwajiin akka dabarsan baruuf oggeeessi qoorannoo isin faana jiraachuu danda'uu kunnis daawanaa manaatif baasiin meeqa akka barbaachisuu hubachuuf nugargaara haata'uu malee jiraachuu ykn akka bahaan yoo barbaadan narssiittii himuu nidandeessu

Qoranno kana keessatti hirmaachuudhaan ogeessoonni dhukkuba sombaa/TB daa'immani irratti qorannoo gochaa jiran odeeffannoo dabalataa akka argatan ni deeggartu.

Gama birootiin qorannoo kana yeroo barbaaddan kamiiyyuutti addaan kutu ykn hirmaachuu dhiisuuf mirgii keessan kan eegamedha.

Namootni adda biroon qorannoo kana keessatti hirmaatan odeeffannoo dhuunfaa keessan arguu ni danda'u. kanaafuu, unka kana irrattiis ta'e sanadoota adda biroo irratti maqaa keessan ykn teessoo Email keessan akka hin mul'anne gochuuf hanga danda'ame hundaatti tataafii barbaachisaa ni gona.

Ogeessi qorannoo kana gaggeessu sirritti isiin hin keesumsisiin yoo hafe qorataa muummee dhaabbata qorannoo dhukkuba sombaa /TB/ KNCV Tuberculosis

Foundation, Itiyoophiyaa – Lakk.: +251 911 405 405.kan ta'an Dr. Ahimad Badruutti bilbiilu ni dandeessu.

Qorannoon kun Biiroo Eegumsa Fayyaa Mootummaa Naannoo Oromiyaa, Qorannoo Dhukkuba Tasaa Hawaasaa tiin kan mirkanaa'ee fi koree kana irraa xalayaa deeggarsaa /mirkana argatee jira. Qorannoon kun dhaabata Joonhopkinssittiin kan mirkanaa'eedha. Akkasumas KNCViin hordoffi fi xintala daataarrati gargaarsa jonhopkinsirra ni argata.

Caassaan qoorannoo kanaa walfakkenya labssii heeliisinkii fi qajeelcha naamusa biyaaleessaa dooctaroota baayoomeedikaala marii waa'ee hirmaatotta ilmaan namarrattii godhanniif yaada kennuu. Haftee issaa laaluu ykn duubiisuu yoo barbaadan issinitti laachuun ni danda'aama.

Akka hirmaataa tokkootti gaaffii mirgaa yoo qabaattan ykn qorannoo kana ilaalchisee komii yoo qabatan teessoo armaan gaditiin nu argachuu dandeessu.

Oromiyaa:

Gamachuu Shumii: Dura Taa'aa

Biiroo Eegumsa Fayyaa Mootummaa Naannoo Oromiyaa, Qorannoo Dhukkuba Tasaa Hawaasaa – IRB

Lakk. Bilbiila: +251922279214

Yunaayitid Steetis Of Ameerikaa:

Johns Hopkins Medical – IRB X

The Johns Hopkins University

Institutional Review Board

Lakk. Bilbiila: (001) 410 955 3008

UNKAA HEEYYAMAMUMMAA HIRMAANNAA QORANNOO DAA'IMMAN/ IJOOLLEE UMRI: WAGGAA 12 - 14

WALIITTI DHUUFEENYA DAA'IMMANI: KILIINIKA YAALIIN/WAL'ANSII ITTI KENNAMU

Qorannoo kana irratti hirmaachuuf osoo hin murteessiin dura gaaffii dabalataa qabduu? Gaaffii yoo qabaattan na gaafachuu dandeessu.

UNKAA HEEYYAMAMUMMAA HIRMAANNAA QORANNOO DAA'IMMAN/ IJOOLLEE UMRI: WAGGAA 12 - 14

WALIITTI DHUUFEENYA DAA'IMMANI: KILIINIKA YAALIIN/WAL'ANSII ITTI KENNAMU

FUULA MALLATTOO

JECHA HEEYYAMAMUMMAA

Qorannoo kana irratti hirmaachuuf heeyyamammaa ta'uu keessanmirkaneessuuf unkaa kana irratti osoo hin mallatteessin dura, dhimmoota armaan gadi sirritti hubadhaa

- Unkaa heeyyamamummaa kana sirritti dubbiseen jira ykn namni naaf dubbisee jira.
- Kaayyoon qorannoo kana isiniif ibsame gaafii keessan hundaaf deebii gahaan isiniif kenname jira.
- Ammas irra deebiidhaan gaaffii dabalataa yoo qabaattan, gaaffii keessan dhiheessuuf mirgi keessan kan eegame ta'uu hubatanii jirtu.
- Kaayyoo fi barbaachisummaan qorannoo kana sirritti isiniif ergi ibsamee booda qorannoo kana irratti hirmaachuuf heeyyamamaa taatanii jirtu.
- Yeroo turtii gaaffii fi deebii kanaa keessatti yeroo barbaadan kamiiyyuutti deebii kennu dhiisuu ykn addaan kutu akka dandeessan hubatanii jirtu.

Maqaa haadhaa/abbaa ykn guddistuu /guddisaa seera qabeessaa____________

Daa'imni keessan qorannoo kana irratti akka hirmaatuuf waliigaltanii jirtuu?

EYYEE _______ MITI _______ (Mallattoo irratti godha)

Maqaa daa'ima (umriin isaa /ishee waggaa ≥12 ol ta'e): ______________________

Qorannoo kana irratti hirmaachuuf waliigaltanii jirtuu? EYYEE______ MITI_______

Maqaa guutuu hirmaataa/ Deebii kennaa(barreessi)	Mallattoo hirmaataa/ Deebii kennaa	Guyyaa (gg/jjj/bbbb)	Sa'aatii
______________________	______________________	___________						

UNKAA HEEYYAMAMUMMAA HIRMAANNAA QORANNOO DAA'IMMAN/ IJOOLLEE UMRI: WAGGAA 12 - 14

WALIITTI DHUUFEENYA DAA'IMMANI: KILIINIKA YAALIIN/WAL'ANSII ITTI KENNAMU

**Maqaa guutuu qorataa/hojjetaa Kiliinikaa (Barreessi)**	**Mallattoo qorataa/hojjetaa kiliinikaa**	**Guyyaa (GG/ jjj/bbbb)**	**Sa'aatii**

**Hirmaataan/ deebii kennaan dubbisuu hin danda'u yoo ta'e, gabateen armaan gadi haa guutamu :*

**Maqaa guutuu ragaa (barrees**	**Mallattoo ragaa**	**Guyyaa (gg/jjj/bbbb)**	**Sa'aatii**

**Ragaan heeyyamamummaa deebii kennaa irratti dhibbaa uumuu hin danda'u.*

Orjinaala/muummee unkaa kanaa galmee/faayilii wajjin walqabsisudhaan Orjinaala/muummee tokko deebii kennaa/hirmaataadhaaf kennaa. Yoo barbaachisaa ta'e waraabbii/koppii dabalataa galmee meedikaalaa dhukkubsataa wajjin waliin qabsisi

UNKAA HEEYYAMAMUMMAA HIRMAANNAA QORANNOO DAA'IMMAN/ IJOOLLEE UMRI: WAGGAA 12 - 14

Waliitti dhuufeenya daa'immani: To'annaa kiliinikaa

SANSAKAA : Garee praagraamaatikii yaali raandamaayizidii qorrannoo ittissa dhukkuba sombaa haawaassa gidduu gala goodhatee afrikaa kibbaatti fi Itoophiyaa

Mata duree gabaabaa: Tajaajiilawwan TPT mana keessatti kennamu

KAN FOOYYA'E: Sansakaa fooyya'e 4.0, gaafa gguyyaa Ebla 19 bara 2013 A.L.I
Unkaa heeyyamamummaa hirmaannaa 4.0, Gaafa guyyaa Ebla19 bara 2013 A.L.I

QORATAA MUUMMEE: Dr. Ahimad Badruu

Bilbiila: +251 911 405 405

Ani maqaan kiyya ________ ogummaan kiyya Narsii/gorsa kennaa yemmuu ta'uu sababa qoroannoo kanaatiif kiliinika ______, jedhamu irraa dhufeen jira. Haaluma kanaan, qorannoo kana irratti akka hirmaattan kabajaadhaan ni gaafadha. Kanaafuu qorannoo kana irratti hirmaachuuf heeyyamamummaa keessan akka nuuf ibsitan isiin gaafanna.

Qorannoon kun daa'ima dhukkuba sombaatif/TB saaxilmanif qoricha ittisa TB lachuf iddon mijaa'aan sadarka manarattimoo kilinikarrati gaaridha waan jedhu murtesa. Tajaajili daa'imni argatuu qajelcha biyolessarratti hunda'a yemuu ta'u, qoranno kanarratti hirmachuf wanti dabalataan daamni fi isinnis gootan hinjiru. Qorannoo kanaratti hirmachu baatanilee tajaajili barbaachissaan daa'imni kilinikaraa argatchu qabuu ni laatamaaf.

Qoranoo kanaratti hirmaachu kessaniff balaan ijoon issinira gahu danda'uu iccitii qabuu dhissudha kannaaf imoo safari heduu bakka kenye jirra.

UNKAA HEEYYAMAMUMMAA HIRMAANNAA QORANNOO DAA'IMMAN/ IJOOLLEE UMRI: WAGGAA 12 - 14

Waliitti dhuufeenya daa'immani: To'annaa kiliinikaa

Maatii keessan keessaa namni tokko dhukkuba sombaa/TB tiin qabame jira. Maatii tokko keessa namni tokko dhukkuba sombaa/TB tiin yoo qabame, kiliinikichi ogeessoota fayyaa isaa gara mana keessanitti ergudhaan miseensi maatii adda biroon kessumaa iyyuu daa'imman adda biroon dhukkuba sombaa/TB tiin qabamuu isaanii ni qorata.

Kanaafuu, kaayyoon qorannoo kanaa bu'aa qabeessummaa tajaajiilawwan qorannoo fi ittiisa dhukkuba sombaa /TB keessummaayyuu daa'imman irratti kan xiyyeeffatedha. Daa'imman salphaaatti dhukkuba sombaa/TB tiin qabamuu ni danda'u. Dhukkubbni sombaa /TB afuura ykn qufaa nama dhukkubba kanaan qabameetiin gara namoota adda biroo fi daa'immanitti darbuu ni danda'a.

Namni kiliinika keenya keessatti qorannoon godhameefi dhukkuba sombaa /TB tiin qabamuun isaa mirkanaa'e hundinuu, qorannoo kana irratti hirmaachuuf fedhii yoo qabaatan ni gaafatamu. Haaluma kanaan, qorannoo kana irratti hirmaachuuf heeyyamammaa yoo taatan, gaaffiiwwan armaan gadi isiniif dhiheesina.

1. Haadha ykn abbaa ykn guddistuun keessan akka isiin qorannoo kana keessatti hirmaataniif heeyyamamummaa isaanii ni gaafanna.
2. Hanga yaalii /wal'ansa keessan xumurtaniitti kaardii /galmee keessan kiliinika keenya keessatti argamu ilaaluu ni dandeessu.

Gaafa yaali Tiibii gootan narsiin sa'aattii meeqa isiinniwajiin akka dabarsan laaluuf oggeeessi qoorannoo issin faana jiraachuu danda'uu kunnis daawanaa mannatif baassin meeqa akka barbaachisuu hubachuuf nugargaara haata'uu

malee hirmaachuu yookin akka bahaanis yoo barbaadan narssiittii himuu nidandeessu

Qoranno kana keessatti hirmaachuudhaan ogeessoonni dhukkuba sombaa/TB daa'immani irratti qorannoo gochaa jiran odeeffannoo dabalataa akka argatan ni deeggartu.

Gama birootiin qorannoo kana yeroo barbaaddan kamiiyyuutti addaan kutu ykn hirmaachuu dhiisuuf mirgii keessan kan eegamedha.

Namootni adda biroon qorannoo kana keessatti hirmaatan odeeffannoo dhuunfaa keessan arguu ni danda'u. kanaafuu, unka kana irrattiis ta'e sanadoota adda biroo irratti maqaa keessan ykn teessoo Email keessan akka hin mul'anne gochuuf hanga danda'ame hundaatti tataafii barbaachisaa ni gona.

Ogeessi qorannoo kana gaggeessu sirritti isiin hin keesumsisiin yoo hafe qorataa muummee dhaabbata qorannoo dhukkuba sombaa /TB/ KNCV Tuberculosis Foundation, Itiyoophiyaa – Lakk.: +251 911 405 405.kan ta'an Dr. Ahimad Badruutti bilbiilu ni dandeessu.

Qorannoon kun Biiroo Eegumsa Fayyaa Mootummaa Naannoo Oromiyaa, Qorannoo Dhukkuba Tasaa Hawaasaa tiin kan mirkanaa'ee fi koree kana irraa xalayaa deeggarsaa /mirkana argatee jira. Qorannoon kun dhaabata Joonhopkinssittiin kan mirkanaa'eedha. Akkasumas KNCViin hordoffi fi xintala daataarrati gargaarsa jonhopkinsirra ni argata.

Waliitti dhuufeenya daa'immani: To'annaa kiliinikaa

Caassaan qoorannoo kanaa walfakkenya labssii heeliisinkii fi qajeelcha naamusa biyaaleessaa dooctaroota baayoomeedikaala marii waa'ee hirmaatotta ilmaan namarrattii godhanniif yaada kennuu. Haftee issaa laaluu ykn duubiisuu yoo barbaadan issinitti laachuun ni danda'aama.

Akka hirmaataa tokkootti gaaffii mirgaa yoo qabaattan ykn qorannoo kana ilaalchisee komii yoo qabatan teessoo armaan gaditiin nu argachuu dandeessu.

Oromiyaa:

Gamachuu Shumii: Dura Taa'aa

Biiroo Eegumsa Fayyaa Mootummaa Naannoo Oromiyaa, Qorannoo Dhukkuba Tasaa Hawaasaa – IRB

Lakk. Bilbiila: +251922279214

Yunaayitid Steetis Of Ameerikaa:

Johns Hopkins Medical – IRB X

The Johns Hopkins University

Institutional Review Board

Lakk. Bilbiila: (001) 410 955 3008

Qorannoo kana irratti hirmaachuuf osoo hin murteessiin dura gaaffii dabalataa qabduu? Gaaffii yoo qabaattan na gaafachuu dandeessu.

UNKAA HEEYYAMAMUMMAA HIRMAANNAA QORANNOO DAA'IMMAN/ IJOOLLEE UMRI: WAGGAA 12 - 14

Waliitti dhuufeenya daa'immani: To'annaa kiliinikaa

FUULA MALLATTOO

JECHA HEEYYAMAMUMMAA

Qorannoo kana irratti hirmaachuuf heeyyamammaa ta'uu keessanmirkaneessuuf unkaa kana irratti osoo hin mallatteessin dura, dhimmoota armaan gadi sirritti hubadhaa

- Unkaa heeyyamamummaa kana sirritti dubbiseen jira ykn namni naaf dubbisee jira.
- Kaayyoon qorannoo kana isiniif ibsame gaafii keessan hundaaf deebii gahaan isiniif kenname jira.
- Ammas irra deebiidhaan gaaffii dabalataa yoo qabaattan, gaaffii keessan dhiheessuuf mirgi keessan kan eegame ta'uu hubatanii jirtu.
- Kaayyoo fi barbaachisummaan qorannoo kana sirritti isiniif ergi ibsamee booda qorannoo kana irratti hirmaachuuf heeyyamamaa taatanii jirtu.
- Yeroo turtii gaaffii fi deebii kanaa keessatti yeroo barbaadan kamiiyyuutti deebii kennu dhiisuu ykn addaan kutu akka dandeessan hubatanii jirtu.

Maqaa haadhaa/abbaa ykn guddistuu /guddisaa seera qabeessaa____________

Daa'imni keessan qorannoo kana irratti akka hirmaatuuf waliigaltanii jirtuu?

EYYEE _______ MITI _______ (Mallattoo irratti godha)

Maqaa daa'ima (umriin isaa /ishee waggaa ≥12 ol ta'e): ______________________

Qorannoo kana irratti hirmaachuuf waliigaltanii jirtuu? EYYEE______ MITI_______

______________________ ______________________ __________

**Maqaa guutuu hirmaataa/ Deebii kennaa(barreessi)**	**Mallattoo hirmaataa/ Deebii kennaa**	**Guyyaa (gg/jjj/bbbb)**	**Sa'aatii**

Sansakaa: Tajaajiilawwan TPT mana keessatti kennamu 4.0, gaafa guyyaa Ebla 19 bara 2013 A.L.I
INGILIIFFAA: Odeeffannoo unkaa heeyyamamummaa hirmaannaa qorannoo daa'imman/ ijoollee umri: waggaa 12 - 14
Ijoollee dhukkubsatan: To'annaa kiliinikaa
UNKAA heeyyamamummaa hirmaannaa 4.0, Gaafa guyyaa Ebla 19 bara 2013 A.L.I
qorataa Muummee: Dr. Ahimad Badruu
Kan mirkaneesse: Biiroo Eegumsa Fayyaa Mootummaa Naannoo Oromiyaa, Qorannoo Dhukkuba Tasaa Hawaasaa – IRB
Guyyaa itti mirkanaa'e: 13 09 2021

UNKAA HEEYYAMAMUMMAA HIRMAANNAA QORANNOO DAA'IMMAN/ IJOOLLEE UMRI: WAGGAA 12 - 14

Waliitti dhuufeenya daa'immani: To'annaa kiliinikaa

Maqaa guutuu qorataa/hojjetaa Kiliinikaa (Barreessi) **Mallattoo qorataa/hojjetaa kiliinikaa** **Guyyaa (GG/ jjj/bbbb)** **Sa'aatii**

**Hirmaataan/ deebii kennaan dubbisuu hin danda'u yoo ta'e, gabateen armaan gadi haa guutamu :*

Maqaa guutuu ragaa (barrees **Mallattoo ragaa** **Guyyaa (gg/jjj/bbbb)** **Sa'aatii**

**Ragaan heeyyamamummaa deebii kennaa irratti dhibbaa uumuu hin danda'u.*

Orjinaala/muummee unkaa kanaa tokkoo galmee/faayilii wajjin walqabsisudhaan Orjinaala/muummee tokkoo deebii kennaa/hirmaataadhaaf kennaa. Yoo barbaachisaa ta'e waraabbii/koppii dabalataa galmee meedikaalaa dhukkubsataa wajjin waliin qabsisi

PARTICIPANT INFORMATION LEAFLET AND
INFORMED CONSENT FORM
Healthcare Workers

PROTOCOL: A pragmatic cluster-randomized trial of community-based contact investigation and initiation of TB preventive therapy in South Africa and Ethiopia

SHORT TITLE: Home-Based TPT Time-and-Motion Assessment

VERSION: Protocol Version 4.0, dated 27 April 2021
Informed Consent Form Version 4.0 dated 27 April 2021

PRINCIPAL INVESTIGATOR: Dr. Ahmed Bedru

TELEPHONE: +251 911 405 405

My name is ___________________________________. I am a researcher with the KNCV Tuberculosis Foundation, Ethiopia in Addis Ababa. I would like to invite you to consider participating in a research study.

In this study we want to learn about how much a program delivering home-based care for TB prevention costs the clinic. To be able to do that, we need to know how much time the program takes for health workers like yourself. If you choose to participate, you will be observed during one or more of your scheduled work shifts by a member of the study team. The study team member will record the types of activities you perform during your workday and how long you spend performing these tasks. This is called a **Time-and-Motion Study**. The main risk of being in this study is loss of confidentiality. We have many preventive measures in place to make sure this does not happen.

PROCEDURES

We are inviting healthcare workers taking part in the CHIP-TB Study to also to take part in a **Time-and-Motion Study.** If you want to take part, you will be observed when you work at the clinic by the researcher. The researcher will write down the different activities you perform during your workday. The person will also write down how long you take to do it.

The research team member may observe you for a part of your workday or all of your workday. You may be observed for only one day or on a few days. The observation days will be randomly selected. “Random selection” is like drawing numbers from a hat. The research team will try to let you know before the time what day or day(s) you can expect to be observed and have your work activities written down. These observations should not take much of your time.

You may also be asked to write down your own work activities and the time that you take to do the activities. We will give you a form to write down the activities that you do. We will also provide

instructions on how to record your activities and how long it takes you to do them. These forms will not take much time to fill out.

The information we learn by observing you will allow us to calculate the costs of a home visit and a clinic visit. This information can help the government decide if home visits should be promoted. This may help groups like the World Health Organization create new guidelines.

RISKS AND BENEFITS
We do not think that being part of this study will create significant risks for you. The main risk of being in this study is loss of confidentiality. It is not possible to guarantee complete privacy. We will try to make the records as private as possible. Your name will not be attached to any report we create based on these observations. The information we collect as part of this research project will only be used for this research study and will not be shared with your supervisor. Your information will not be used in future research studies.

You will not receive any direct benefit from participating in this study. The data collected from your participation may help to improve TB and HIV prevention services in your community and in other communities.

You will not be paid to take part in this study.

VOLUNTARY PARTICIPATION
You do not have to agree to be in this study. If you do not want to join the study, it will not affect your job. And, if you do participate, your job will not be affected by your time or your answers. You can decide to stop taking part in the study at any time without giving a reason.

If you have any questions about your rights as a research participant, or if you think you have not been treated fairly, you may contact Dr. Ahmed Bedru at the KNCV Tuberculosis Foundation, Ethiopia – Tel: +251 911 405 405

ETHICAL APPROVAL
This study protocol has been submitted to the Oromia Regional Health Bureau Public Emergency and Health Research directorate IRB and written approval has been granted by that committee. This study has also been approved by the Johns Hopkins IRB. Johns Hopkins will help Aurum Institute monitor the study and analyze the data. The study has been structured in accordance with the Declaration of Helsinki (last updated: October 2013) and the National Ethical Guidelines which deals with the recommendations guiding doctors in biomedical research involving human participants. I can obtain a copy for you if you wish to review it.

If you want any information regarding your rights as a research participant, or have complaints regarding this research study, you may contact:

<u>Oromia:</u>
Gemechu Shumi: Chairperson
Oromia Regional Health Bureau Public Emergency and Health Research Directorate IRB
Telephone number: +251922279214

<u>United States of America:</u>
Johns Hopkins Medical – IRB X

PARTICIPANT INFORMATION LEAFLET AND INFORMED CONSENT FORM Healthcare Workers

The Johns Hopkins University
Institutional Review Board
Telephone number: (001) 410 955 3008

This independent committee is established to help protect the rights of research participants and gave written approval for the study protocol.

PARTICIPANT INFORMATION LEAFLET AND
INFORMED CONSENT FORM
Healthcare Workers

SIGNATURE PAGE

STATEMENT OF CONSENT

Before you sign this consent form, make sure of the following:

- You have read this informed consent form, or someone has read it to you.
- This study has been explained to you and had your questions answered.
- You understand you can ask more questions at any time.
- You have understood everything that has been explained to you and you consent to participate in this research study.
- You understand that you can without prejudice withdraw your consent at any time. If that happened, any data collected about me for the purposes of the study would be destroyed, unless I give consent for it to be retained.

**Participant's Name and Second/middle name (Print)**	**Participant's Signature / Thumbprint**	**Date (dd/mmm/yyyy)**	**Time**

**Study Staff conducting consent discussion Name and Second/middle name (Print)**	**Study Staff Signature**	**Date (dd/mmm/yyyy)**	**Time**

**For participants who are unable to read, also complete the signature block below:*

**Witness' Name and Second/middle name (Print)**	**Witness' Signature**	**Date (dd/mmm/yyyy)**	**Time**

**Witness is impartial and was present for the entire consent process.*

Retain one original Informed Consent Form on file. Offer participant the other original signed consent.

PARENT/LEGAL GUARDIAN INFORMATION LEAFLET AND INFORMED CONSENT FORM FOR HOUSEHOLD CONTACTS OF TUBERCULOSIS INDEX PARTICIPANTS

CHILD CONTACT: CONTROL CLINIC

PROTOCOL: A pragmatic cluster-randomized trial of community-based contact investigation and initiation of TB preventive therapy in South Africa and Ethiopia

SHORT TITLE: Home Based TPT

VERSION: Protocol Version 4.0, dated 27 April 2021
Informed Consent Form Version 4.0, dated 27 April 2021

PRINCIPAL INVESTIGATOR: Dr Ahmed Bedru

TELEPHONE: +251 911 405 405

Good day, I am____________, a nurse/community health worker from the _____ clinic. I would like to invite you to consider your child's participation in a research study. We are working together with the clinic staff here doing research about TB.

This research study will determine if the clinic or the home is the best place for children exposed to TB to get checked for and given medicine to prevent TB. All of the care your child will receive is by national guideline. You and your child do not need to do anything extra to be a part of this study. If you do not want to be a part of this study, you can still get all the care your child needs at the clinic.

The main risk of being in this study is loss in confidentiality. We have many preventive measures in place to make sure this does not happen.

I am inviting your child to be part of this study because someone in your household has TB. TB is spread by people coughing and others close by breathing in the air. Children who live in a house with someone who has TB are very likely to be infected with TB. That is why when someone has TB, the clinic sends someone to the house to check the other family members and see if they might have TB.

Our study is looking at how well a home visit program works to get children a medicine that they need to keep from getting TB. This medicine is recommended by the Ethiopian Government for children who live with someone with TB. You can decide to accept or not accept this medicine. Before getting this medicine, children need to be checked for TB and, when appropriate, HIV. This is because people who are living with HIV are also very likely to be infected with TB. The check can happen at the clinic or in the home. In this study, we are looking at whether it will be

useful for clinic staff to make home visits. Some children will be checked and given medicine at the clinic and some will be checked and given medicine in the home. Children at this clinic will be checked and given medicine at the clinic.

You may be randomly selected to take part in two brief costing interviews. "Random selection' is like choosing people by flipping a coin. If you are selected, the interviews will take place on the telephone or in person. Each interview will each be about 30 minutes long. We will ask questions about how much it costs you and your family to bring your child to the clinic. The first interview will happen soon after your child is checked for TB. The second interview will be in about 3 months, after your child finishes treatment.

We also want to know how much the program is costing the clinic. Some clinic visits will be "randomly selected" for observation by a research staff member. All the information the researcher sees or hears will be kept confidential/secret. You will be asked at the start of your visit if it is ok with you that the researcher stays in the room. It is ok to say no. It will not affect the care you and your child receive in the clinic. It will also not affect your participation in the study.

Being part of this study is your choice. You don't have to agree. Your child will still be able to get proper treatment at the clinic even if you don't want to be in this study. Also, if you agree and then change your mind, you can stop being in the study at any time.

If you agree to be part of this study, we will:

1. Explain the study to your child (if they are 12 years of age or older) and ask if they choose to agree.
2. Use the information in your child's clinic records to see how well the care given in the clinic works for patients. We will ask to look over your child's clinic record for the next year.
3. We *may* contact you to participate in an interview about how much visiting the clinic for your child's care costs your household.

We will keep the information from your child's clinic chart confidential. Only the study and clinic staff will know this information.

Your child does not need to do anything extra to be part of this research study. Through this research, we hope to improve the TB services the community receives.

You will not be paid to take part in this study. If you are selected for the interviews, we will pay you 50 ETB in cell phone airtime, for each interview you complete, to cover your time expenses.

If you feel the study staff has not treated you properly, you can get hold of Dr Ahmed Bedru at the KNCV Tuberculosis Foundation, Ethiopia – Tel: +251 911 405 405.

This study has been approved by the Oromia Regional Health Bureau IRB and written approval has been granted by that committee. This study has also been approved by the Johns Hopkins IRB. Johns Hopkins will help KNCV monitor the study and analyze the data.

PARENT/LEGAL GUARDIAN INFORMATION LEAFLET AND INFORMED CONSENT FORM FOR HOUSEHOLD CONTACTS OF TUBERCULOSIS INDEX PARTICIPANTS

CHILD CONTACT: CONTROL CLINIC

The study has been structured in accordance with the Declaration of Helsinki (last updated: October 2013) and National Ethical Guidelines which deal with the recommendations guiding doctors in biomedical research involving human participants. I can obtain a copy for you if you wish to review it.

If you want any information regarding your rights as a participant, or have complaints regarding this study, you may contact:

Oromia:
Gemechu Shumi: Chair Person
Oromia Regional Health Bureau Public Emergency and Health Research Directorate IRB
Telephone number: +251922279214

United States of America:
Johns Hopkins Medical – IRB X
The Johns Hopkins University
Institutional Review Board
Telephone number: (001) 410 955 3008

Do you have any questions or concerns before you make a decision about being in this study? If so, please ask me.

PARENT/LEGAL GUARDIAN INFORMATION LEAFLET AND INFORMED CONSENT FORM FOR HOUSEHOLD CONTACTS OF TUBERCULOSIS INDEX PARTICIPANTS

CHILD CONTACT: CONTROL CLINIC

SIGNATURE PAGE FOR THE COLLECTION OF YOUR CHILD'S RECORDS

STATEMENT OF CONSENT

Before you sign this consent form for the collection of your child's records, make sure of the following:

- You have read this informed consent form, or someone has read it to you.
- This study has been explained to you and had your questions answered.
- You understand you can ask more questions at any time.
- You have understood everything that has been explained to you and you consent that your child can participate in this research study.
- You understand that you can without prejudice withdraw your consent at any time. If that happened, any data collected about you and your child for the purposes of the study would be destroyed, unless you give consent for it to be retained.

Name of Parent / Legal Guardian ____________________________

Name of Child: ________________ Age of child____________

Do you agree for your child to be in this study? YES_______ NO_______ (Please initial)

______________________	______________________	__________	
**Parent/Legal Guardian Name and Second/middle name (Print)**	**Parent/Legal Guardian Signature/ Thumbprint**	**Date (dd/mmm/yyyy)**	**Time**
______________________	______________________	__________	
**Clinic Staff conducting consent Name and Second/middle name (Print)**	**Clinic Staff conducting consent Signature/Thumbprint**	**Date (dd/mmm/yyyy)**	**Time**

**For participants who are unable to read, also complete the signature block below:*

______________________	______________________	__________	
**Witness' Name and Second/middle name (Print)**	**Witness' Signature**	**Date (dd/mmm/yyyy)**	**Time**

**Witness is impartial and was present for the entire consent process.*

Retain one original Informed Consent Form on file. Offer participant the other original signed consent. Place a copy in medical records if applicable.

PARENT/LEGAL GUARDIAN INFORMATION LEAFLET AND INFORMED CONSENT FORM FOR HOUSEHOLD CONTACTS OF TUBERCULOSIS INDEX PARTICIPANTS

CHILD CONTACT: CONTROL CLINIC

SIGNATURE PAGE FOR THE COSTING INTERVIEW

STATEMENT OF CONSENT

Before, you sign this consent form to take part in the costing interview, make sure of the following

- You have read this informed consent form, or someone has read it to you.
- This study, including the costing interviews, has been explained to you and you had your questions answered.
- You understand you can ask more questions at any time.
- You have understood everything that has been explained to you and you consent to participate in the costing interviews.
- You understand that you can without prejudice withdraw your consent at any time. If that happened, any data collected about you and your child for the purposes of the study would be destroyed, unless you give consent for it to be retained.

If you **AGREE** to take part in an interview about personal costs related to your child's treatment, please sign your name or make your mark below.

**Participant's Name and Second/middle name (Print)**	**Participant's Signature / Thumbprint**	**Date (dd/mmm/yyyy)**	**Time**
**Clinic Staff conducting consent discussion Name and Second/middle name (Print)**	**Clinic Staff Signature**	**Date (dd/mmm/yyyy)**	**Time**

**For participants who are unable to read, also complete the signature block below:*

**Witness' Name and Second/middle name (Print)**	**Witness' Signature**	**Date (dd/mmm/yyyy)**	**Time**

**Witness is impartial and was present for the entire consent process.*

Retain one original Informed Consent Form on file. Offer participant the other original signed consent. Place a copy in medical records if applicable.

PARENT/LEGAL GUARDIAN INFORMATION LEAFLET AND INFORMED CONSENT FORM FOR HOUSEHOLD CONTACTS OF TUBERCULOSIS INDEX PARTICIPANTS

CHILD CONTACT: INTERVENTION CLINIC

PROTOCOL: A pragmatic cluster-randomized trial of community-based contact investigation and initiation of TB preventive therapy in South Africa and Ethiopia

SHORT TITLE: Home Based TPT

VERSION: Protocol Version 4.0, dated 27 April 2021
Informed Consent Form Version 4.0, dated 27 April 2021

PRINCIPAL INVESTIGATOR: Dr Ahmed Bedru

TELEPHONE: +251 911 405 405

Good day, I am____________, a nurse/community health worker from the _______ clinic. I would like to invite you to consider your child’s participation in a research study. We are working together with the clinic staff here doing research about TB.

This research study will determine if the clinic or the home is the best place for children exposed to TB to get checked for and given medicine to prevent TB. All of the care your child will receive is by national guideline. You and your child do not need to do anything extra to be a part of this study. If you do not want to be a part of this study, you can still get all the care your child needs at the clinic.

The main risk of being in this study is loss in confidentiality. We have many preventive measures in place to make sure this does not happen.

I am inviting your child to be part of this study because someone in your household has TB. TB is spread by people coughing and others close by breathing in the air. Children who live in a house with someone who has TB are very likely to be infected with TB. That is why when someone has TB, the clinic sends someone to the house to check the other family members and see if they might have TB.

Our study is looking at how well a home visit program works to get children a medicine that they need to keep from getting TB. This medicine is recommended by the Ethiopian Government for children who live with someone with TB. You can decide to accept or not accept this medicine. Before getting this medicine, children need to be checked for TB and, when appropriate, for HIV. This is because people who are living with HIV are also very likely to be infected with TB. The check can happen at the clinic or in the home. In this study, we are looking at whether it will be useful for clinic staff to make home visits. Some children will be checked and given medicine at the clinic and some will be checked and given medicine in the home. Children who get care at this clinic will be checked and given medicine in the home.

You may be randomly selected to take part in two brief costing interviews. "Random selection' is like choosing people by flipping a coin. If you are selected, the interviews will take place on the telephone or in person. Each interview will each be about 30 minutes long. We will ask questions about how much it costs you and your family to participate in the home visit program. The first interview will happen soon after your child is checked for TB. The second interview will be in about 3 months, after your child finishes treatment.

We also want to know how much the program is costing the clinic. Some home visits will be "randomly selected" for observation by a research staff member. All the information the researcher sees or hears will be kept confidential/secret. You will be asked at the start of your visit if it is ok with you that the researcher stays in the room. It is ok to say no. It will not affect the care you and your child receive in the clinic. It will also not affect your participation in the study.

Being part of this study is your choice. You do not have to agree. Your child will still be able to get proper treatment at the clinic even if you do not want to be in this study. Also, if you agree and then change your mind, you can stop being in the study at any time.

If you agree to be part of this study, we will:

1. Explain the study to your child (if they are 12 years of age or older) and ask if they choose to agree.
2. Visit your home and check your child for symptoms of TB. We will only visit your home after getting permission from your household member who has TB.
3. If your child does not have signs of TB, we will offer them medicine to keep them from getting TB. If your child has signs of TB, we will ask you to bring them to the clinic to be checked for TB.
4. Use the information in your child's clinic records to see how well the care given in the home, works for participants. We will ask to look over your child's clinic record for the next year.
5. We *may* contact you to participate in an interview about how much the home visit for your child's care costs your household.

We will keep the information from your child's clinic chart confidential. Only the study and clinic staff will know this information.

Your child does not need to do anything extra to be part of this research study. Through this research, we hope to improve the TB services the community receives.

You will not be paid to take part in this study. If you are selected for the interviews, we will pay you 50 ETB in cell phone airtime, for each interview you complete, to cover your time expenses.

If you feel the study staff has not treated you properly, you can get hold of Dr Ahmed Bedru at the KNCV Tuberculosis Foundation, Ethiopia – Tel: +251 911 405 405.

This study has been approved by the Oromia Regional Health Bureau IRB and written approval has been granted by that committee. T This study has also been approved by the Johns Hopkins IRB. Johns Hopkins will help KNCV monitor the study and analyze the data.

PARENT/LEGAL GUARDIAN INFORMATION LEAFLET AND INFORMED CONSENT FORM FOR HOUSEHOLD CONTACTS OF TUBERCULOSIS INDEX PARTICIPANTS

CHILD CONTACT: INTERVENTION CLINIC

The study has been structured in accordance with the Declaration of Helsinki (last updated: October 2013) and National Ethical Guidelines which deal with the recommendations guiding doctors in biomedical research involving human participants. I can obtain a copy for you if you wish to review it.

If you want any information regarding your rights as a participant, or have complaints regarding this study, you may contact:

Oromia:
Gemechu Shumi: Chairperson
Oromia Regional Health Bureau Public Emergency and Health Research Directorate IRB
Telephone number: +251922279214

United States of America:
Johns Hopkins Medical – IRB X
The Johns Hopkins University
Institutional Review Board
Telephone number: (001) 410 955 3008

Do you have any questions or concerns before you make a decision about being in this study? If so, please ask me.

PARENT/LEGAL GUARDIAN INFORMATION LEAFLET AND INFORMED CONSENT FORM FOR HOUSEHOLD CONTACTS OF TUBERCULOSIS INDEX PARTICIPANTS

CHILD CONTACT: INTERVENTION CLINIC

SIGNATURE PAGE FOR THE HOME-BASED TB PREVENTION INTERVENTION

STATEMENT OF CONSENT

Before you sign this consent form for the home-based TB prevention intervention, make sure of the following:

- You have read this informed consent form, or someone has read it to you.
- This study has been explained to you and had your questions answered.
- You understand you can ask more questions at any time.
- You have understood everything that has been explained to you and you consent to that your child can participate in this research study.
- You understand that you can without prejudice withdraw your consent at any time. If that happened, any data collected about you and your child for the purposes of the study would be destroyed, unless you give consent for it to be retained.

Name of Parent / Legal Guardian ____________________________

Name of Child: _________________ Age of child_____________

Do you agree for your child to be in this study? YES_______ NO_______ (Please initial)

_________________________	_________________________	___________	☐☐☐☐
**Parent/Legal Guardian Name and Second/middle name (Print)**	**Parent/Legal Guardian Signature/ Thumbprint**	**Date (dd/mmm/yyyy)**	**TIME**
_________________________	_________________________	___________	☐☐☐☐
**Clinic Staff conducting consent Name and Second/middle name (Print)**	**Clinic Staff conducting consent Signature/Thumbprint**	**Date (dd/mmm/yyyy)**	**TIME**

**For participants who are unable to read, also complete the signature block below:*

_________________________	_________________________	___________	☐☐☐☐
**Witness' Name and Second /middle name (Print)**	**Witness' Signature**	**Date (dd/mmm/yyyy)**	**TIME**

**Witness is impartial and was present for the entire consent process.*

Retain one original Informed Consent Form on file. Offer participant the other original signed consent. Place a copy in medical records if applicable.

PARENT/LEGAL GUARDIAN INFORMATION LEAFLET AND INFORMED CONSENT FORM FOR HOUSEHOLD CONTACTS OF TUBERCULOSIS INDEX PARTICIPANTS

CHILD CONTACT: INTERVENTION CLINIC

SIGNATURE PAGE FOR THE COSTING INTERVIEW

STATEMENT OF CONSENT

Before, you sign this consent form to take part in the costing interview, make sure of the following.

- You have read this informed consent form, or someone has read it to you.
- This study, including the costing interviews, has been explained to you and you had your questions answered.
- You understand you can ask more questions at any time.
- You have understood everything that has been explained to you and you consent to participate in the costing interviews.
- You understand that you can without prejudice withdraw your consent at any time. If that happened, any data collected about you and your child for the purposes of the study would be destroyed, unless you give consent for it to be retained.

If you **AGREE** to take part in an interview about personal costs related to your child's treatment, please sign your name or make your mark below.

______________________	______________________	______________	☐☐☐☐
**Participant's Name and Second/middle name (Print)**	**Participant's Signature / Thumbprint**	**Date (dd/mmm/yyyy)**	**Time**

______________________	______________________	______________	☐☐☐☐
**Clinic Staff conducting consent discussion Name and Second/middle name (Print)**	**Clinic Staff Signature**	**Date (dd/mmm/yyyy)**	**Time**

**For participants who are unable to read, also complete the signature block below:*

______________________	______________________	______________	☐☐☐☐
**Witness' Name and Second/middle name (Print)**	**Witness' Signature**	**Date (dd/mmm/yyyy)**	**Time**

**Witness is impartial and was present for the entire consent process.*

Retain one original Informed Consent Form on file. Offer participant the other original signed consent. Place a copy in medical records if applicable.

UNKAA HEEYYAMAMUMMAA HIRMAANNAA MAATIWWANII /GUDDISTUU SEERA QABEESSA QORANNOO DAA'IMMAN DHUKKUBA SOMBAA TIIN QABAMANI

Waliitti dhuufeenya daa'immani: To'annaa kiliinikaa

SANSAKAA: Garee praagraamaatikii yaali raandamaayizidii qorrannoo ittissa dhukkuba sombaa haawaassa gidduu gala goodhatee afrikaa kibbaatti fi Itoophiyaa

Mata duree gabaabaa: Tajaajiilawwan TPT mana keessatti kennamu

KAN FOOYYA'E: Sansakaa fooyya'e 4.0, gaafa gguyyaa Ebla 19 bara 2013 A.L.I
Unkaa heeyyamamummaa hirmaannaa 4.0, Gaafa guyyaa Ebla 19 bara 2013 A.L.I

QORATAA MUUMMEE: Dr. Ahimad Badruu
Bilbiila: +251 911 405 405

Ani maqaan kiyya ________ ogummaan kiyya Narsii/hojjetaa fayyaa hawaasa kiliinika yemmuu ta'uu sababa qoroannoo kanaatiif kiliinika ______, jedhamu irraa dhufeen jira. Haaluma kanaan, qorannoo kana ogeessoota qorannoo dhukkuba sombaa /TB kiliinika kanaa wajjin hojjechaa jirra. Kanaafuu qorannoo kana irratti daa'imni akka hirmaatuuf heeyyamamummaa keessan akka nuuf ibsitan isiin gaafanna.

Qorannoon kun daa'ima dhukkuba sombaatif/TB saaxilmanif qoricha ittisa TB lachuf iddon mijaa'aan sadarka manarattimoo kilinikarrati gaaridha waan jedhu murtesa. Tajaajili daa'imni argatuu qajelcha biyolessarratti hunda'a yemuu ta'u, qoranno kanarratti hirmachuf wanti dabalataan daamni fi isinnis gootan hinjiru. Qorannoo kanaratti hirmachu baatanilee tajaajili barbaachissaan daa'imni kilinikaraa argatchu qabuu ni laatamaaf.

Qoranoo kanaratti hirmaachu kessaniff balaan ijoon issinira gahu danda'uu iccitii qabuu dhissudha kannaaf imoo safari heduu bakka kenye jirra.

UNKAA HEEYYAMAMUMMAA HIRMAANNAA MAATIWWANII /GUDDISTUU SEERA QABEESSA QORANNOO DAA'IMMAN DHUKKUBA SOMBAA TIIN QABAMANI

Waliitti dhuufeenya daa'immani: To'annaa kiliinikaa

Maatii keessan keessa namni tokko dhukkuba sombaa /TB tiin qabamuun isaa qorannoodhaan waan hubatameef daa'ima keessan irratti qorannoon akka godhamuuf isiin afeera. Dhukkubni sombaa/TB nama dhukkuba kanaan qabame irraa gara nama adda birootti afuura baafachuudhaan dadarbuu ni danda'a. Maatii tokko keessaa namni tokko dhukkuba sombaatiin yoo qabame carraa daa'imman maatii kanaa dhukkubichaan qabamu ol'aanaadha. Maatii tokko keessa namni tokko dhukkuba sombaa /TB tiin qabamuun isaa bataluma mirkanaa'etti kiliinikni keenya ogeessota isaa ergudhaan miseensonni maatii kanaa adda biroon dhukkuba sombaa /TB tiin qabamuu isaanii ni qorata.

Kaayyoon qorannoo kanaa sagantaan yaalii/wal'ansa /qoriicha mana keessatti ogeessoota fayyaatiin daa'immaniif kennamu daa'imman dhukkuba sombaa TB irraa ittiisuuf hangam bu'aa qabeessa akka ta'e qorachuudha. Qoriichi kun Mootummaa Itoophiyaa daa'imman dhukkuba sombaa/TB tiin qabamaniitiif kan ajajamedha. Kanaafuu, qoriicha kana fayyadamuu ykn fayyadamuu dhiisuuf mirgii keessan kan eegamedha. Daa'imman keessan qoriicha kana fudhachuu osoo hin jalqabiin dura ogeessaan qoratamuu qabu. Qorannoon kunis kiliinika keessatti ykn mana keessatti godhamu ni danda'a. kanaafuu, kaayyoo qorannoo kanaa inni adda biroon immoo daa'imman dhukkuba kanaan qabamaniif ogeessoonni fayyaa gara mana daa'imman maatiiwwan daa'imman kanaa deemani wal'ansa kennun kan irraa barbaadamu yoo ta'e addaan baasudha. Daa'imman muraasaaf kiliinika keessatti qorannoon godhame qoriichi kan kennamu yemmuu ta'u daa'imman hafaniif/muraasaaf immoo mana isaanii keessatti qorannoon godhameefi qoriichi kan kennamuuf ni ta'a. Haaluma kanaan, daa'imman gara kiliinika keenya dhufani qoratamani qoriichi

kennameef irra deebidhaan mana isaanii keessatti qoratamanii qoriichi kiliinika keessatti akka kennamuuf ni godhama.

Gaafiif Deebii baasiirratti ergaa gabaabduu lamarratti hirmaachuuf akka tassaatti filatamu dandeessa. 'Akka tassaa filatamuu' Jeechuun saantima garagalchuun namoota hirmaatan filachuudha. Carraan filatamuu yoo sigahee gaafiif deebiin bilbilaan ykn qaamaan argamtee godhama.gaafiif deebiin kun tokko tokkoon daqiqaa 30 fudhata. Gaafileen waa'ee baasii siifi maatiin kee daa'ima keessan gara kilinikaa fiduuf baaftan illaalatta. Gaafiif deebiin isa duraa daa'imni kessan erga tajaajilii qooricha Tibii godhameef booda. Gaafiif deebiin lamafaa, ji'a 3 booda daa'imni qooricha issaa erga xumuree godhama.

Dabalataan baasiin sagantaan kun kilinika irrattii fidu baruu barbaana.kilinika muraassa akka tassaattii filatamanirratti hojeetaan miseensa qoranoo hubannoo ni goodha. Oddeefanoon qorataan argee ykn dhageefatee hundii icciittiidhaan qabama. Gaafiif deebii isa tokkoffaa gotuuratti heyyama kee yoo arganee qoorataan kuttaa tokko kesatti siiwaliin ta'uu danda'aa. Yoo hinheyyamnee garuu lakki jechuu ni dandessa kunnis tajaajila daa'imni fi atti kilinika kana irraa argatan akkasuma hirmaanaa qooranoo irratti gootaniif dhibaa fiduu hin qabuu.

Qorannoo kana keessatti hirmaachuun heeyyamamummaa /fedhii keessan irratti kan hundaa'e ni ta'a. kanaafuu, qorannoo kana keessatti hirmaachuuf dirqamni hin jiru. Yoo qorannoo kana keessatti hirmaachuuf heeyyamammaa hin ta'iin haftan illee kiliinikichi daa'ima keessaniif qorannoo/wal'ansaa fi qoriicha barbaachisaa kennu hin dhiisu. Itti dabalataaniis, ergii qorannoo kana keessatti hirmaachuu jalqabdanii booda yeroo kamiiyyuutti addaan kutuu ykn hirmaachuu dhiisuuf mirgii keessan kan egamedha.

UNKAA HEEYYAMAMUMMAA HIRMAANNAA MAATIWWANII /GUDDISTUU SEERA QABEESSA QORANNOO DAA'IMMAN DHUKKUBA SOMBAA TIIN QABAMANI

Waliitti dhuufeenya daa'immani: <u>To'annaa</u> kiliinikaa

Qorannoo kana keessatti hirmaachuuf heeyyamammaa yoo taatan,

:

1. Kaayyoo qorannoo kanaa daa'ima keessaniif (umri isaa /ishee waggaa 12 ykn isaa ol yoo ta'e. Ibsudhaan daa'immni keessan qorannoo kana keessatti hirmaachuuf fedhii kan qabu /qabduu ta'uu isaa/ishee addaan baafadha.
2. Wal'ansii/qoriichi daa'ima keessaniif kiliinika keessan keessatti kenname hangam bu'aa qabeessa akka ta'e beekuu yoo barbaadan galmee daa'ima keessani kiliinika keessatti argamu ilaaluu ni dandeessu. Bara itti anuus galmee daa'ima keessani kiliinika keenya keessatti argamuu ilaaluuf mirgii keessan kan eegamedha.
3. Baasii daawanaa kilinikaa kunuunsa daa'imaattiif baaftan ilaalchisee gaafiif deebii irrati akka hirmaatan issiin yaamuu dandeeyna.

Odeeffannoo daa'imman keessani galmee kiliinika keessatti galmeeffaman hundaa iccitaan ni eegna. Odeeffannoo kana ilaaluuf ykn argu kan danda'u ogeessoota kiliinika qofa ni ta'a

Daa'immni keessan qorannoo kana irratti hirmaachuuf wanti dabalataa isiin irraa barbaadamu hin jiru. Qorannoo kana keessatti hirmaachuun keessan haala eegumsa fayyaa daa'immanii fooyyeessuu fi qorannoo dhukkuba sombaa /TB irratti godhamaa jiru jajabeessuuf gumaacha ol'aanaa ni qaba.

Qooranoo kanna irrattii hirmaanaa gootuuf kafalttiin kafalamuu hin jiru. gaafiif deebiif yoo filatamtee, baasii bakka busuuf gaafiif deebii tokko yoomuu xumuurtuu moobaa'illi keettirratti caardii abaa 50 siifgutama.

Ogeessi qorannoo kana gaggeessu sirritti isiin hin keesumsisiin yoo hafe qorataa muummee dhaabbata qorannoo dhukkuba sombaa /TB/ KNCV Tuberculosis

Foundation, Itiyoophiyaa – Lakk.: +251 911 405 405.kan ta'an Dr. Ahimad Badruutti bilbiilu ni dandeessu.

Qorannoon kun Biiroo Eegumsa Fayyaa Mootummaa Naannoo Oromiyaa, Qorannoo Dhukkuba Tasaa Hawaasaa tiin kan mirkanaa'ee fi koree kana irraa xalayaa deeggarsaa /mirkana argatee jira. Qorannoon kun dhaabata Joonhopkinssittiin kan mirkanaa'eedha. Akkasumas KNCViin hordoffi fi xintala daataarrati gargaarsa jonhopkinsirra niargata.
Caassaan qoorannoo kanaa walfakkenya labssii heeliisinkii fi qajeelcha naamusa biyaaleessaa dooctaroota baayoomeedikaala marii waa'ee hirmaatotta ilmaan namarrattii godhanniif yaada kennuu. Haftee issaa laaluu ykn duubiisuu yoo barbaadan issinitti laachuun ni danda'aama.

Akka hirmaataa tokkootti gaaffii mirgaa yoo qabaattan ykn qorannoo kana ilaalchisee komii yoo qabatan teessoo armaan gaditiin nu argachuu dandeessu.

<u>Oromiyaa:</u>
Gamachuu Shumii: Dura taa'aa
Biiroo Eegumsa Fayyaa Mootummaa Naannoo Oromiyaa, Qorannoo Dhukkuba Tasaa Hawaasaa – IRB
Lakk. Bilbiila: +251922279214

<u>Yunaayitid Steetis Of Ameerikaa:</u>
Johns Hopkins Medical – IRB X
The Johns Hopkins University
Institutional Review Board
Lakk. Bilbiila: (001) 410 955 3008

UNKAA HEEYYAMAMUMMAA HIRMAANNAA MAATIWWANII /GUDDISTUU SEERA QABEESSA QORANNOO DAA'IMMAN DHUKKUBA SOMBAA TIIN QABAMANI

Waliitti dhuufeenya daa'immani: To'annaa kiliinikaa

Qorannoo kana irratti hirmaachuuf osoo hin murteessiin dura gaaffii dabalataa qabduu? Gaaffii yoo qabaattan na gaafachuu dandeessu .

UNKAA HEEYYAMAMUMMAA HIRMAANNAA MAATIWWANII /GUDDISTUU SEERA QABEESSA QORANNOO DAA'IMMAN DHUKKUBA SOMBAA TIIN QABAMANI

Waliitti dhuufeenya daa'immani: To'annaa kiliinikaa

FUULA MALLATTOO RAGAA DAA'IMA KEESSANII SASAABUU

JECHA HEEYYAMAMUMMAA

Heeyyamma qorannoo kana irratti ragaa daa'ima keessanii sasaabuu keeyna mirkaneessuuf unkaa kana irratti mallatteessun dura, dhimmoota armaan gadi sirritti hubadhaa

- Unkaa heeyyamamummaa kana sirritti dubbiseen jira ykn namni naaf dubbisee jira.
- Kaayyoon qorannoo kana isiniif ibsame gaafii keessan hundaaf deebii gahaan isiniif kenname jira.
- Ammas irra deebiidhaan gaaffii dabalataa yoo qabaattan, gaaffii keessan dhiheessuuf mirgi keessan kan eegame ta'uu hubatanii jirtu.
- Kaayyoo fi barbaachisummaan qorannoo kana sirritti isiniif ergi ibsamee booda qorannoo kana irratti daa'imnii keessan hirmaachuu akka danda'uu heeyyamamaa taatanii jirtu.
- Yeroo turtii gaaffii fi deebii kana keessatti yeroo barbaadan kamiiyyuutti deebii kennu dhiisuu ykn addaan kutu akka dandeessan akkasumaas hirmaachuu yoo hin barbaanee ragaaleen kaniitan akka balessinuu hubatanii jirtu.

Maqaa haadhaa/abbaa ykn guddistuu /guddisaa seera qabeessaa____________

Maqaa daa'ima: _________________ Umrii daa'ima: _________________

Qorannoo kana irratti hirmaachuuf waliigaltanii jirtuu? EYYEE______ MITI_______

______________________ ______________________ __________

**Maqaa guutuu hirmaataa/ Deebii kennaa(barreessi)**	**Mallattoo hirmaataa/ Deebii kennaa**	**Guyyaa (gg/jjj(bbbb)**	**Sa'aatii**

Sansakaa: Tajaajiilawwan TPT mana keessatti kennamu 4.0, gaafa guyyaa Ebla 19 bara 2013 A.L.I

INGILIIFFAA: Unkaa heeyyamamummaa hirmaannaa maatiwwanii /guddistuu seera qabeessa qorannoo daa'imman dhukkuba sombaa tiin qabamani - waliitti dhuufeenya daa'imman /ijoollee: To'annaa kiliinikaa - 4.0, Gaafa guyyaa Ebla 19 bara 2013 A.L.I
qorataa Muummee: Dr. Ahimad Badruu
Kan mirkaneesse: Biiroo Eegumsa Fayyaa Mootummaa Naannoo Oromiyaa, Qorannoo Dhukkuba Tasaa Hawaasaa – IRB
Guyyaa itti mirkanaa'e: 13/09/2021

UNKAA HEEYYAMAMUMMAA HIRMAANNAA MAATIWWANII /GUDDISTUU SEERA QABEESSA QORANNOO DAA'IMMAN DHUKKUBA SOMBAA TIIN QABAMANI

Waliitti dhuufeenya daa'immani: To'annaa kiliinikaa

Maqaa guutuu qorataa/hojjetaa Kiliinikaa (Barreessi)	Mallattoo qorataa /hojjetaa kiliinikaa	Guyyaa (gg/ jjj/bbbb)	Sa'aatii

**Hirmaataan/ deebii kennaan dubbisuu hin danda'u yoo ta'e, gabateen armaan gadi haa guutamu :*

Maqaa guutuu ragaa (barreessi)	Mallattoo ragaa	Guyyaa (gg/jjj/bbbb)	Sa'aatii

**Ragaan heeyyamamummaa deebii kennaa irratti dhibbaa uumuu hin danda'u.*

Orjinaala/muummee unkaa kanaa galmee/faayilii wajjin walqabsisudhaan Orjinaala/muummee tokko deebii kennaa/hirmaataadhaaf kennaa. Yoo barbaachisaa ta'e waraabbii/koppii dabalataa galmee meedikaalaa dhukkubsataa wajjin waliin qabsisi

FUULA MALLATTOO GAAFFIiF DEEBII BAASII

JECHA HEEYYAMAMUMMAA

Heeyyamma qorannoo kana irratti ragaa daa'ima keessanii sasaabuu keeyna mirkaneessuuf unkaa kana irratti mallatteessun dura, dhimmoota armaan gadi sirritti hubadhaa

- Unkaa heeyyamamummaa kana sirritti dubbiseen jira ykn namni naaf dubbisee jira.
- Kaayyoon qorannoo kanaa akkasumas gaaffiif deebii baasii isiniif ibsamee, gaafii keessan hundaaf deebii gahaan isiniif kenname jira.
- Ammas irra deebiidhaan gaaffii dabalataa yoo qabaattan, gaaffii keessan dhiheessuuf mirgi keessan kan eegame ta'uu hubatanii jirtu.

UNKAA HEEYYAMAMUMMAA HIRMAANNAA MAATIWWANII /GUDDISTUU SEERA QABEESSA QORANNOO DAA'IMMAN DHUKKUBA SOMBAA TIIN QABAMANI

Waliitti dhuufeenya daa'immani: To'annaa kiliinikaa

- Kaayyoo fi barbaachisummaan qorannoo kana sirritti isiniif ergi ibsamee booda gaaffif debbii baasii irratti hirmaachuuf heeyyamamaa taatanii jirtu.
- Yeroo turtii gaaffii fi deebii kanaa keessatti yeroo barbaadan kamiiyyuutti deebii kennu dhiisuu ykn addaan kutu akka dandeessan akkasumaas ragaaleen kaniitan akka balessinuu hubatanii jirtu.

Gaaffif deebii baasii dhunfaa wal'ansa daa'imaa ilaalchissee sigaafanuuf heyyamamaa yoo taatee Maqaa fi malatoo kee armaan gadittii guutii.

**Maqaa guutuu hirmaataa/ Deebii kennaa(barreessi)**	**Mallattoo hirmaataa/ Deebii kennaa**	**Guyyaa (gg/jjj(bbbb)**	**Sa'aatii**

**Maqaa guutuu qorataa/hojjetaa kilinikaa bareessi**	**Mallattoo qorataa/hojjetaa kilinikaa**	**Guyyaa (gg/jii/bbbb)**	**Sa'aatii**

**Hirmaataan/ deebii kennaan dubbisuu hin danda'u yoo ta'e, gabateen armaan gadi haa guutamu :*

**Maqaa guutuu ragaa /barressi**	**Mallattoo ragaa**	**Guuyyaa (gg/jii/bbbb)**	**Sa'aatii**

**Ragaan heeyyamamummaa deebii kennaa irratti dhibbaa uumuu hin danda'u.*

Orjinaala/muummee unkaa kanaa galmee/faayilii wajjin walqabsisudhaan Orjinaala/muummee tokko deebii kennaa/hirmaataadhaaf kennaa. Yoo barbaachisaa ta'e waraabbii/koppii dabalataa galmee meedikaalaa dhukkubsataa wajjin waliin qabsisi

Sansakaa: Tajaajiilawwan TPT mana keessatti kennamu 4.0, gaafa guyyaa Ebla 19 bara 2013 A.L.I

INGILIIFFAA: Unkaa heeyyamamummaa hirmaannaa maatiwwanii /guddistuu seera qabeessa qorannoo daa'imman dhukkuba sombaa tiin qabamani - waliitti dhuufeenya daa'imman /ijoollee: To'annaa kiliinikaa - 4.0, Gaafa guyyaa Ebla 19 bara 2013 A.L.I
qorataa Muummee: Dr. Ahimad Badruu
Kan mirkaneesse: Biiroo Egumsa Fayyaa Mootummaa Naannoo Oromiyaa, Qorannoo Dhukkuba Tasaa Hawaasaa – IRB
Guyyaa itti mirkanaa'e: 13/09/2021

UNKAA HEEYYAMAMUMMAA HIRMAANNAA MAATIWWANII /GUDDISTUU SEERA QABEESSA QORANNOO DAA'IMMAN DHUKKUBA SOMBAA TIIN QABAMANI

Waliitti dhuufeenya daa'immani: To'annaa kiliinikaa

UNKAA HEEYYAMAMUMMAA HIRMAANNAA MAATIWWANII /GUDDISTUU SEERA QABEESSA QORANNOO DAA'IMMAN DHUKKUBA SOMBAA TIIN QABAMANI

<u>WALIITTI DHUUFEENYA DAA'IMMAN /IJOOLLEE</u>: KILIINIKA <u>YAALIIN/WAL'ANSII ITTI KENNAMU</u>

SANSAKAA: Garee praagraamaatikii yaali raandamaayizidii qorrannoo ittissa dhukkuba sombaa haawaassa gidduu gala goodhatee afrikaa kibbaatti fi Itoophiyaa

Mata duree gabaabaa: Tajaajiilawwan TPT mana keessatti kennamu

KAN FOOYYA'E: Sansakaa fooyya'e 4.0, gaafa gguyyaa Ebla 19 bara 2013 A.L.I
Unkaa heeyyamamummaa hirmaannaa 4.0, Gaafa guyyaa Ebla 19bara 2013 A.L.I

QORATAA MUUMMEE: Dr. Ahimad Badruu

Bilbiila: +251 911 405 405

Ani maqaan kiyya ________ ogummaan kiyya Narsii/hojjetaa fayyaa hawaasa kiliinika yemmuu ta'uu sababa qoroannoo kanaatiif kiliinika ______, jedhamu irraa dhufeen jira. Haaluma kanaan, qorannoo kana ogeessoota qorannoo dhukkuba sombaa /TB kiliinika kanaa wajjin hojjechaa jirra. Kanaafuu qorannoo kana irratti daa'imni akka hirmaatuuf heeyyamamummaa keessan akka nuuf ibsitan isiin gaafanna.

Qorannoon kun daa'ima dhukkuba sombaatif/TB saaxilmanif qoricha ittisa TB lachuf iddon mijaa'aan sadarka manarattimoo kilinikarrati gaaridha waan jedhu murtesa. Tajaajili daa'imni argatuu qajelcha biyolessarratti hunda'a yemuu ta'u, qoranno kanarratti hirmachuf wanti dabalataan daamni fi isinnis gootan hinjiru. Qorannoo kanaratti hirmachu baatanilee tajaajili barbaachissaan daa'imni kilinikaraa argatchu qabuu ni laatamaaf.

UNKAA HEEYYAMAMUMMAA HIRMAANNAA MAATIWWANII /GUDDISTUU SEERA QABEESSA QORANNOO DAA'IMMAN DHUKKUBA SOMBAA TIIN QABAMANI

WALIITTI DHUUFEENYA DAA'IMMAN /IJOOLLEE: KILIINIKA YAALIIN/WAL'ANSII ITTI KENNAMU

Qoranoo kanaratti hirmaachu kessaniff balaan ijoon issinira gahu danda'uu iccitii qabuu dhissudha kannaaf imoo safari heduu bakka kenye jirra.

Maatii keessan keessa namni tokko dhukkuba sombaa /TB tiin qabamuun isaa qorannoodhaan waan hubatameef daa'ima keessan irratti qorannoon akka godhamuuf isiin afeera. Dhukkubni sombaa/TB nama dhukkuba kanaan qabame irraa gara nama adda birootti afuura baafachuudhaan dadarbuu ni danda'a. Maatii tokko keessaa namni tokko dhukkuba sombaatiin yoo qabame carraa daa'imman maatii kanaa dhukkubichaan qabamu ol'aanaadha. Maatii tokko keessa namni tokko dhukkuba sombaa /TB tiin qabamuun isaa bataluma mirkanaa'etti kiliinikni keenya ogeessota isaa ergudhaan miseensonni maatii kanaa adda biroon dhukkuba sombaa /TB tiin qabamuu isaanii ni qorata.

Kaayyoon qorannoo kanaa sagantaan yaalii/wal'ansa /qoriicha mana keessatti ogeessoota fayyaatiin daa'immaniif kennamu daa'imman dhukkuba sombaa TB irraa ittiisuuf hangam bu'aa qabeessa akka ta'e qorachuudha. Qoriichi kun Mootummaa Itoophiyaattin daa'imman dhukkuba sombaa/TB tiin qabamaniitiif kan ajajamedha. Kanaafuu, qoriicha kana fayyadamuu ykn fayyadamuu dhiisuuf mirgii keessan kan eegamedha. Daa'imman keessan qoriicha kana fudhachuu osoo hin jalqabiin dura ogeessaan qoratamuu qabu. Qorannoon kunis kiliinika keessatti ykn mana keessatti godhamu ni danda'a. kanaafuu, kaayyoo qorannoo kanaa inni adda biroon immoo daa'imman dhukkuba kanaan qabamaniif ogeessoonni fayyaa gara mana daa'imman maatiiwwan daa'imman kanaa deemani wal'ansa kennun kan irraa barbaadamu yoo ta'e addaan baasudha. Daa'imman muraasaaf kiliinika keessatti qorannoon godhame qoriichi kan kennamu yemmuu ta'u daa'imman hafaniif/muraasaaf immoo

mana isaanii keessatti qorannoon godhameefi qoriichi kan kennamuuf ni ta'a. Haaluma kanaan, daa'imman gara kiliinika keenya dhufani qoratamani qoriichi kennameef irra deebidhaan mana isaanii keessatti qoratamanii qoriichi achumatti/manuma isaanii keessatti akka kennamuuf ni godhama.

Gaafiif Deebii baasiirratti ergaa gabaabduu lamarratti hirmaachuuf akka tassaatti filatamu dandeessa. 'Akka tassaa filatamuu' Jeechuun saantima garagalchuun namoota hirmaatan filachuudha. Carraan filatamuu yoo argatee gaafiif deebiin bilbilaan ykn qaamaan dhuftee godhama. gaafiif deebiin kun tokko tokkoon dheerina daqiqaa 30 qaba. Gaafi siifi maatiin kee baasii daa'imanii hirmaanaa sagantaa tajaajiila mana keessatti goodhamuuf baastan illaalatta. Gaafiif deebiin isa duraa daa'imni kessan jalqaba tajaajila qooricha Tibii irratti godhama. Gaafiif deebiin lamafaa, ji'a 3 booda, daa'imni qooricha issaa erga xumuree godhama.

Dabalataan baasiin sagantaan kun kilinika irrattii fidu baruu barbaana. dawanaa manaa akka tassaattii filatamanirratti hojeetaan miseensa qoranoo hubannoo ni goodha. Oddeefanoon qorataan argee ykn dhageefatee hundii icciittiidhaan qabama. Gaafiif deebii isa tokkoffaa gotuuratti heyyama kee yoo arganee qoorataan kuttaa tokko kesatti siiwaliin ta'uu danda'aa. Yoo hinheyyamnee garuu lakki jechuu ni dandessa kunnis tajaajila daa'imni fi atti kilinika kana irraa argatan akkasuma hirmaanaa qooranoo irratti gootaniif dhibaa fiduu hin qabuu.

Qorannoo kana keessatti hirmaachuun heeyyamamummaa /fedhii keessan irratti kan hundaa'e ni ta'a. kanaafuu, qorannoo kana keessatti hirmaachuuf

dirqamni hin jiru. Yoo qorannoo kana keessatti hirmaachuuf heeyyamammaa hin ta'iin haftan illee kiliinikichi daa'ima keessaniif qorannoo/wal'ansaa fi qoriicha barbaachisaa kennu hin dhiisu. Itti dabalataaniis, ergii qorannoo kana keessatti hirmaachuu jalqabdanii booda yeroo kamiiyyuutti addaan kutuu ykn hirmaachuu dhiisuuf mirgii keessan kan egamedha.

Qorannoo kana keessatti hirmaachuuf heeyyamammaa yoo taatan,

1. Kaayyoo qorannoo kanaa daa'ima keessaniif (umri isaa /ishee waggaa 12 ykn isaa ol yoo ta'e. Ibsudhaan daa'immni keessan qorannoo kana keessatti hirmaachuuf fedhii kan qabu /qabduu ta'uu isaa/ishee addaan baafadha.
2. Ogeessoonni kiliinika keenya gara mana keessanii dhufudhaan daa'ima keessan irratti mallattoon dhukkuba sombaa/TB kan mul'atu ta'uu isaa ni qoratu. Gara mana keessanii dhufnee qorannoo kana kan gaggeessinu yemmuu miseensa maatii keessani dhukkuba sombaa/TB tiin qabame irraa heeyyama arganu qofaadha.
3. Daa'ima keessan irratti mallattoon dhukkuba sombaa /TB Mul'atu hin jiru yoo ta'e, gara fulduratti daa'immni keessan dhukkuba sombaa /TB tiin akka hin qabamneef qoriicha ittiisa dhukkuba sombaa ni kennina. Haata'u malee, daa'ima keessan irratti mallattoon dhukkuba sombaa /TB kan mul'atu yoo ta'e, gara fulduratti daa'ima keessan gara kiliinika keenya fidanii qorannoon dhukkuba sombaa /TB dabalataa akka godhamuuf haala ni mijeesina.
4. Wal'ansii/qoriichi daa'ima keessaniif mana keessan keessatti kenname hangam bu'aa qabeessa akka ta'e beekuu yoo barbaadan galmee daa'ima keessani kiliinika keessatti argamu ilaaluu ni dandeessu. Bara itti anuus galmee daa'ima keessani kiliinika keenya keessatti argamuu ilaaluuf mirgii keessan kan eegamedha.

WALIITTI DHUUFEENYA DAA'IMMAN /IJOOLLEE: KILIINIKA YAALIIN/WAL'ANSII ITTI KENNAMU

5. Baasii daawanaan manaa kunuunsa daa'imaattiif baaftan ilaalchisee gaafiif deebii irrati akka hirmaatan issiin yaamuu dandeeyna.

Odeeffannoo daa'imman keessani galmee kiliinika keessatti galmeeffaman hundaa iccitaan ni eegna. Odeeffannoo kana ilaaluuf ykn argu kan danda'u ogeessoota kiliinika qofa ni ta'a

Daa'immni keessan qorannoo kana irratti hirmaachuuf wanti dabalataa isiin irraa barbaadamu hin jiru. Qorannoo kana keessatti hirmaachuun keessan haala eegumsa fayyaa daa'immanii fooyyeessuu fi qorannoo dhukkuba sombaa /TB irratti godhamaa jiru jajabeessuuf gumaacha ol'aanaa ni qaba.

Qooranoo kana irrattii hirmaanaa gootuuf kafalttiin kafalamuu hin jiruu. gaafiif deebiif yoo filatamtee, baasii bakka busuuf gaafiif deebii tokko yoomuu xumuurtuu moobaa'illi keettirratti caardii abaa 50 siifgutama.

Ogeessi qorannoo kana gaggeessu sirritti isiin hin keesumsisiin yoo hafe qorataa muummee dhaabbata qorannoo dhukkuba sombaa /TB/ KNCV Tuberculosis Foundation, Itiyoophiyaa – Lakk.: +251 911 405 405.kan ta'an Dr. Ahimad Badruutti bilbiilu ni dandeessu.

Qorannoon kun Biiroo Eegumsa Fayyaa Mootummaa Naannoo Oromiyaa, Qorannoo Dhukkuba Tasaa Hawaasaa tiin kan mirkanaa'ee fi koree kana irraa xalayaa deeggarsaa /mirkana argatee jira. Qorannoon kun dhaabata Joonhopkinssittiin kan mirkanaa'eedha. Akkasumas KNCViin hordoffi fi xintala daataarrati gargaarsa jonhopkinsirra ni argata.

UNKAA HEEYYAMAMUMMAA HIRMAANNAA MAATIWWANII /GUDDISTUU SEERA QABEESSA QORANNOO DAA'IMMAN DHUKKUBA SOMBAA TIIN QABAMANI

WALIITTI DHUUFEENYA DAA'IMMAN /IJOOLLEE: KILIINIKA YAALIIN/WAL'ANSII ITTI KENNAMU

Caassaan qoorannoo kanaa walfakkenya labssii heeliisinkii fi qajeelcha naamusa biyaaleessaa dooctaroota baayoomeedikaala marii waa'ee hirmaatotta ilmaan namarrattii godhanniif yaada kennuu. Haftee issaa laaluu ykn duubiisuu yoo barbaadan issinitti laachuun ni danda'aama.

Akka hirmaataa tokkootti gaaffii mirgaa yoo qabaattan ykn qorannoo kana ilaalchisee komii yoo qabatan teessoo armaan gaditiin nu argachuu dandeessu.

Oromiyaa:

Gamachuu Shumii: Dura taa'aa

Biiroo Eegumsa Fayyaa Mootummaa Naannoo Oromiyaa, Qorannoo Dhukkuba Tasaa Hawaasaa – IRB

Lakk. Bilbiila: +251922279214

UNKAA HEEYYAMAMUMMAA HIRMAANNAA MAATIWWANII /GUDDISTUU SEERA QABEESSA QORANNOO DAA'IMMAN DHUKKUBA SOMBAA TIIN QABAMANI

<u>WALIITTI DHUUFEENYA DAA'IMMAN /IJOOLLEE</u>: KILIINIKA <u>YAALIIN/WAL'ANSII ITTI KENNAMU</u>

<u>Yunaayitid Steetis Of Ameerikaa:</u>

Johns Hopkins Medical – IRB X

The Johns Hopkins University

Institutional Review Board

Lakk. Bilbiila: (001) 410 955 3008

Qorannoo kana irratti hirmaachuuf osoo hin murteessiin dura gaaffii dabalataa qabduu? Gaaffii yoo qabaattan na gaafachuu dandeessu .

UNKAA HEEYYAMAMUMMAA HIRMAANNAA MAATIWWANII /GUDDISTUU SEERA QABEESSA QORANNOO DAA'IMMAN DHUKKUBA SOMBAA TIIN QABAMANI

<u>WALIITTI DHUUFEENYA DAA'IMMAN /IJOOLLEE</u>: <u>KILIINIKA YAALIIN/WAL'ANSII ITTI KENNAMU</u>

FUULA MALLATTOO QOORICHA ITTISSA TiiBii MANAA MANARATTI HUNDAA'EE GIDDUU GALA GODHATEE

JECHA HEEYYAMAMUMMAA

Qorannoo qooricha ittissa tiibii manaa manaratti hundaa'ee gidduu gala godhatee kana irratti hirmaachuuf heeyyamammmaa ta'uu keessanmirkaneessuuf unkaa kana irratti osoo hin mallatteessin dura, dhimmoota armaan gadi sirritti hubadhaa

- Unkaa heeyyamamummaa kana sirritti dubbiseen jira ykn namni naaf dubbisee jira.
- Kaayyoon qorannoo kana isiniif ibsame gaafii keessan hundaaf deebii gahaan isiniif kenname jira.
- Ammas irra deebiidhaan gaaffii dabalataa yoo qabaattan, gaaffii keessan dhiheessuuf mirgi keessan kan eegame ta'uu hubatanii jirtu.
- Kaayyoo fi barbaachisummaan qorannoo kana sirritti isiniif ergi ibsamee booda qorannoo kana irratti daa'imnii keessan hirmaachuu akka danda'uu heeyyamamaa taatanii jirtu.
- Yeroo turtii gaaffii fi deebii kana keessatti yeroo barbaadan kamiiyyuutti deebii kennu dhiisuu ykn addaan kutu akka dandeessan akkasumaas hirmaachuu yoo hin barbaanee ragaaleen kaniitan akka balessinuu hubatanii jirtu.

Maqaa haadhaa/abbaa ykn guddistuu /guddisaa seera qabeessaa____________

Maqaa daa'ima: _______________ Umrii daa'ima: _________________

Qorannoo kana irratti hirmaachuuf waliigaltanii jirtuu? EYYEE______ MITI_______

______________________ ______________________ __________

Sansakaa: Tajaajiilawwan TPT mana keessatti kennamu 4.0, gaafa guyyaa Ebla 19 bara 2013 A.L.I

INGILIIFFAA: Unkaa heeyyamamummaa hirmaannaa maatiwwanii /guddistuu seera qabeessa qorannoo daa'imman dhukkuba sombaa tiin qabamani - waliitti dhuufeenya daa'imman /ijoollee: kiliinika yaaliin/wal'ansii itti kennamu - 4.0, Gaafa guyyaa Ebla 19 bara 2013 A.L.I

qorataa Muummee: Dr. Ahimad Badruu

Kan mirkaneesse: Biiroo Eegumsa Fayyaa Mootummaa Naannoo Oromiyaa, Qorannoo Dhukkuba Tasaa Hawaasaa – IRB

Guyyaa itti mirkanaa'e: 13/09/2021

UNKAA HEEYYAMAMUMMAA HIRMAANNAA MAATIWWANII /GUDDISTUU SEERA QABEESSA QORANNOO DAA'IMMAN DHUKKUBA SOMBAA TIIN QABAMANI

WALIITTI DHUUFEENYA DAA'IMMAN /IJOOLLEE: KILIINIKA YAALIIN/WAL'ANSII ITTI KENNAMU

Maqaa guutuu hirmaataa/ Deebii kennaa(barreessi)	Mallattoo hirmaataa/ Deebii kennaa	Guyyaa (gg/jjj(bbbb)	Sa'aatii

Maqaa guutuu qorataa/hojjetaa Kiliinikaa (Barreessi)	Mallattoo qorataa /hojjetaa kiliinikaa	Guyyaa (gg/ jjj/bbbb)	Sa'aatii

**Hirmaataan/ deebii kennaan dubbisuu hin danda'u yoo ta'e, gabateen armaan gadi haa guutamu :*

Maqaa guutuu ragaa (barreessi)	Mallattoo ragaa	Guyyaa (gg/jjj/bbbb)	Sa'aatii

**Ragaan heeyyamamummaa deebii kennaa irratti dhibbaa uumuu hin danda'u.*

Orjinaala/muummee unkaa kanaa galmee/faayilii wajjin walqabsisudhaan Orjinaala/muummee isaa deebiikennaa/hirmaataadhaaf kennaa. Yoo barbaachisaa ta'e waraabbii/koppii dabalataa galmee meedikaalaa dhukkubsataa wajjin waliin qabsisi.

FUULA MALLATTOO GAAFFIiF DEEBII BAASII

JECHA HEEYYAMAMUMMAA

Heeyyamma qorannoo kana irratti ragaa daa'ima keessanii sasaabuu keeyna mirkaneessuuf unkaa kana irratti mallatteessun dura, dhimmoota armaan gadi sirritti hubadhaa

- Unkaa heeyyamamummaa kana sirritti dubbiseen jira ykn namni naaf dubbisee jira.

UNKAA HEEYYAMAMUMMAA HIRMAANNAA MAATIWWANII /GUDDISTUU SEERA QABEESSA QORANNOO DAA'IMMAN DHUKKUBA SOMBAA TIIN QABAMANI

WALIITTI DHUUFEENYA DAA'IMMAN /IJOOLLEE: KILIINIKA YAALIIN/WAL'ANSII ITTI KENNAMU

- Kaayyoon qorannoo kanaa akkasumas gaaffiif deebii baasii isiniif ibsamee, gaafii keessan hundaaf deebii gahaan isiniif kenname jira.
- Ammas irra deebiidhaan gaaffii dabalataa yoo qabaattan, gaaffii keessan dhiheessuuf mirgi keessan kan eegame ta'uu hubatanii jirtu.
- Kaayyoo fi barbaachisummaan qorannoo kana sirritti isiniif ergi ibsamee booda gaaffif debbii baasii irratti hirmaachuuf heeyyamamaa taatanii jirtu.
- Yeroo turtii gaaffii fi deebii kanaa keessatti yeroo barbaadan kamiiyyuutti deebii kennu dhiisuu ykn addaan kutu akka dandeessan akkasumaas ragaaleen kaniitan akka balessinuu hubatanii jirtu.

Gaaffif deebii baasii dhunfaa wal'ansa daa'imaa ilaalchissee sigaafanuuf heyyamamaa yoo taatee Maqaa fi malatoo kee armaan gadittii guutii.

______________	______________	______________	☐☐☐☐
**Maqaa guutuu hirmaataa/ Deebii kennaa(barreessi)**	**Mallattoo hirmaataa/ Deebii kennaa**	**Guyyaa (gg/jjj(bbbb)**	**Sa'aatii**
______________	______________	______________	☐☐☐☐
**Maqaa guutuu qorataa/hojjetaa kilinikaa bareessi**	**Mallattoo qorataa/hojjetaa kilinikaa**	**Guyyaa (gg/jii/bbbb)**	**Sa'aatii**

____

**Hirmaataan/ deebii kennaan dubbisuu hin danda'u yoo ta'e, gabateen armaan gadi haa guutamu :*

______________	______________	______________	☐☐☐☐
**Maqaa guutuu ragaa /barressi**	**Mallattoo ragaa**	**Guuyyaa (gg/jii/bbbb)**	**Sa'aatii**

**Ragaan heeyyamamummaa deebii kennaa irratti dhibbaa uumuu hin danda'u.*

Sansakaa: Tajaajiilawwan TPT mana keessatti kennamu 4.0, gaafa guyyaa Ebla 19 bara 2013 A.L.I
INGILIIFFAA: Unkaa heeyyamamummaa hirmaannaa maatiwwanii /guddistuu seera qabeessa qorannoo daa'imman dhukkuba sombaa tiin qabamani - waliitti dhuufeenya daa'imman /ijoollee: kiliinika yaaliin/wal'ansii itti kennamu - 4.0, Gaafa guyyaa Ebla 19 bara 2013 A.L.I
qorataa Muummee: Dr. Ahimad Badruu
Kan mirkaneesse: Biiroo Eegumsa Fayyaa Mootummaa Naannoo Oromiyaa, Qorannoo Dhukkuba Tasaa Hawaasaa – IRB
Guyyaa itti mirkanaa'e: 13/09/2021

UNKAA HEEYYAMAMUMMAA HIRMAANNAA MAATIWWANII /GUDDISTUU SEERA QABEESSA QORANNOO DAA'IMMAN DHUKKUBA SOMBAA TIIN QABAMANI

WALIITTI DHUUFEENYA DAA'IMMAN /IJOOLLEE: KILIINIKA YAALIIN/WAL'ANSII ITTI KENNAMU

Orjinaala/muummee unkaa kanaa galmee/faayilii wajjin walqabsisudhaan Orjinaala/muummee tokko deebii kennaa/hirmaataadhaaf kennaa. Yoo barbaachisaa ta'e waraabbii/koppii dabalataa galmee meedikaalaa dhukkubsataa wajjin walin qabsisi.

Unka barreeffama odeeffannoo hirmaatotaa fi heeyyama odeeffannoo irratti hundaa'e
Hojjetoota kunuunsa fayyaa

Pirotokolii: Kilaastaraa hojii irra ooluu hawaasa irratti hundaa'ee yaalamtoota waliin waa'ee namoota qunnamtii qaban ilaalchisee qorannoo nasibaa taasifamuu fi Afriikaa Kibbaa fi Itiyoophiyaa keessatti inishiyeeshinii wal'ansa ittiisa TB

MATA DUREE GABAABDUU: Gamaagama yeroo fi hojii wal'ansa ittiisa TB mana keessatti kennamu
Waraabbii: Maxxansa pirotokolii: 4.1, Ebla 19/2013
Unka heeyyama odeeffannoo irrati hundaa'e 4.0, Ebla 19/2013
Raawwataa qo'annoo ol'aanaa: D/R Ahimad Badruu
Lakk. Bilbilaa+251 900 405 405

Maqaan koo _______________ jedhama. KNCV Tiyubarkilosisi Finfinneetti argamu Itiyoophiyaa keessatti qo'annoo kanan gaggeessuudha. Qo'annoo kana irratti hirmaannaa akka gootan isiin afeeree jira.

Sababa qo'annoo kanaan sagantaa ittiisa TB mana keessatti gaggeeffamu kiliinika hangam akka bu'aa qabeessa taasisu baruun ni barbaadna. Kana gochuu akka dandeenyuu sagantaan kun hojjettoota fayyaa isiin fakkaatan sa'aatii hangamii akka fuudhatu hubachuu ni barbaadna. Hirmaannaa gochuuf kan heeyyamtan yoo ta'e yeroo dabree hojii keessanii yeroo tokko ykn sanaa ol miseensa garee qo'annootiin doo'iin isin irratti gaggeeffamu ni danda'a. Miseensi garee qo'annoo guyyaa isin hojii irraa taatan akaakuu gochaalee isiin raawwatanii fi yemmuu gochaa kana raawwatan dheerinaa yeroo isin jalaa fuudhate ni galmeessa kun qo'annoo yeroo fi sochii jedhamee beekama.

Unka barreeffama odeeffannoo hirmaatotaa fi heeyyama odeeffannoo irratti hundaa'e

Hojjetoota kunuunsa fayyaa

Qo'annoo kana keessatti haalli sodaa ijoon jiraatu odeeffannoon dhuunfaa namoota birootiin baramuu isaati. Haalli kun akka hin uumamne gochuuf tarkaanfii ittiisaa hedduu qabna.

ADEEMSA HOJIMAATAA

Hojjettootni kununsa fayyaa qo'annoo CHIP –TB keessatti akka hirmaatan akkasumas qo'annoo yeroo fi sochii keessatti akka hirmaatan ni affeerra. Fedhii hirmaachuu yoo qabaatan hojii keessan kiliinika keessatti yemmuu raawwatan qo'annoo gaggeessaadhaaf xinxala isaa jala ni galtu. Gaggeessaan qo'annoo guyyaa hojii kanatti gochaalee adda addaa isin raawwatan barreeffamaan ni kataba. Dabalataanis gaggeessaan qo'annoo hojii kana raawwachuuf yeroo hangamii akka isiin jala fudhatu barreeffamaan ni qabata.

Miseensi garee qo'annoo do'ii hojii guyyaa walakkaa ykn guutuu isin irratti gaggeessuu ni danda'a.

Do'ii kan gaggeessu guyyaa tokko qofa ykn guyyoota muraasa ta'u ni danda'a. Guyyoota qo'annaa itti gaggeessu nasibaan kan filatamanii dha "filannoo nasibaa" akka carraa baasuu kan fakkaatudha. Gareen qo'annoo guyyoota do'ii itti gaggeessu dursee isiin beeksisuuf yaalii kan godhu yoo ta'u sochii hojii keessanii barreeffamaan ni qubachisa. Do'iileen kunneen yeroo keessan baay'inaan fudhachuu hin qabaatan.

Sochii hojii keessanii fi hojiiwwan kana raawwachuudhaaf yeroo isin jalaa fudhate barreeffamaan akka qubachiftan gaafatamu ni dandeessu. Gochaalee raawwatan unkaa ittiin katabdan isiniif ni kennina. Dabalataanis gochaalee

raawwatanii fi yeroo gochaalee kana raawwachuuf isin jalaa fudhate akkamitti galmeessuu akka qabaatan qajeelfamoota agarsisan isiniif ni kennina. Unka kana guutuudhaaf yeroon dheeraa hin fudhatu.

Isin irratti do'ii gaggeessuudhaan odeeffannoon arganu baasilee do'ii kiliinikatti gaggeeffamuu fi manatti taasifamu shallaguuf nu dandeesisa. Odeeffannoon kun mootummaan do'ii manaa manatti taasifamu jajabeessuu kan qabaatu ykn kan hin qabaanee ta'u isaa murteessuuf isa dandeesisa. Kunis gareewwan kanneen akka dhaabbata fayyaa addunyaa fakkaatan qajeelfamoota haaraa akka baasan isaan gargaaruu ni danda'a.

HAALOTA SODAA FI FAAYIDAAWWAN

Qo'annoo kana irratti hirmaachuu keessaniin haala sodaa ulfaataa ta'e ni jira jenne hin amannu. Halli sodaan ijoon qo'annoo kana irratti jiraatu odeeffannoon dhuunfaa namoota biroo biratti beekamu isaati. Guutumaan guutuutti odeeffannoowwan namoota birootiin akka hin beekamne wabii kennuun hin danda'amu. Maqaan keessan do'ii kana irratti hundaa'udhaan gabaasa qopheessinu kamiiyyuu irratti hin qubatu. Kaayyoo pirojaktii qo'annoo kanaatiif odeeffannoon sasaabne qo'annoo kana qofaaf faayidaa irra kan ooluu yoo ta'u itti gaafatamaan keessan akka beeku hin taasifamu. Odeeffannoon isin ilaallatu qo'annoowwan gara fulduraa jiraatan keessatti faayidaa irra hin oolu.

Qo'annoo kana keessatti hirmaannaa gochuu keessaniin faayidaan kallattidhaan argatan hin jiraatu. Sababa hirmaannaa keessaniin odeeffannoon sasaabne hawaasa keessan keessatti akkasumas haawaasa biroo keessatti tajaajila ittiisa TB fi HIV fooyyeessuuf gargaaruu ni danda'a.

Unka barreeffama odeeffannoo hirmaatotaa fi heeyyama odeeffannoo irratti hundaa'e
Hojjetoota kunuunsa fayyaa

Qo'annoo kana keessatti hirmaachuu keessaniin kanfaltiin isiniif raawwatamu hin jiru.

Hirmaannaa fedhii mataa ofii isaatiin taasifamu
Qo'annoo kana keessatti hirmaachuu dhiisuu ni dandeessu. Qo'annoo kana keessatti hirmaachuu kan hin barbaadnee yoo ta'e dhiibbaan isin irratti taasifamu kamiiyyuu hin jiru.

Hirmaannaa kan godhan yoo ta'e yeroo fi deebilee isin nuuf kennitaniin hojii keessan irratti dhibbaan uumamu hin jiru. Sababa kamiiyyuu kennuun osoo isin hin barbaachisiin yeroo kamiiyyuu qo'annoo kana irratti hirmaannaa gootan dhaabuuf murteessuu ni dandeessu.

Qo'annoo kana irratti hirmaachuudhaan mirgoota jiran ilaalchisee gaafii yoo qabaatan ykn karaa sirrii hin taaneen kan keessumeeftan yoo ta'e D/r Ahimad Badruu faawundeeshinii KNCV Tiyubarkilosisi Itiyoophiyaa Lakk. Bilbilaa +251 911 405 405 bilbiluu ni dandeessu.

MIRKANEEFFANNAA NAAMUSAA

Pirotokoliin qo'annoo kun biiroo fayyaa Naannoo Oromiyaatti Daayireektoreetti qorannoo tasaa fi fayyaa uummataa IRV tiif kan dhihaate yoo ta'u qo'annoon koree kanaan mirkaneeffanaan barreeffamaan irratti kenname jira. Dablataaniis qo'annoon kun Jons Hopkins IRB tiin mirkaneeffannaan irratti kenname jira. Oram institiyutiin qo'annicha akka hordofuu fi odeeffannoowwan akka xinxalu Jons Hopkins deeggarsa isaaf ni taasisa. Qo'annoon kan qindeeffame bu'uura

lasbii heelsink (yeroo dhumaatiif kan fooyya'e Onkololeessa 2006) akkasumas hirmaannaa namootaa kan qabaatu qo'annoo baayoo meedikaalaatiin yaadolee doktaroonni ittiin qajeelfaman kan qabate bu'uura qajeelfama naamusa biyyooleessaatiin dha. Kana ilaaluu yoo barbaadan waraabii isiniif kennu ni dandeenya.

Qo'annoo kana irratti hirmaachuu keessaniin mirgoota qabaattan ilaalchisee odeeffannoo kamiiyyuu yoo barbaaddan ykn qo'annoo ilaalchisee komii yoo qabaatan lakkoofsota bilbilaa kanaa gaditiin bilbiluu ni dandeessu:

Oromiyaa:

Gammachuu Shumii, Dura taa'aa

Biiroo Faayyaa Naannoo Oromiyaatti Daayireektoreetti Qorannoo Tasaa fi Fayyaa Uummataa IRB

Lakk. Bilbilaa +251 19 22279214

Yunaayitid Steetis of Ameeriikaa

Jons Hopkins Meedikaal- IRB X

Z Jons Hopkins Yunivarsitii

Inistitiushinaal Riviwu Ward

Lakk. Bilbilaa (001) 410 955 3008

Koreen walabaan kun mirga hirmaatoota qo'annoo egsisuuf kan hundeeffamee yoo ta'u pirotokolii qo'annootiif mirkaneessa barreeffamaa kenne jira.

Unka barreeffama odeeffannoo hirmaatotaa fi heeyyama odeeffannoo irratti hundaa'e

Hojjetoota kunuunsa fayyaa

FUULA MALLATTOO

IBSA HEEYYAMAMUMMAA

Unka heeyyamamummaa kana mallatteessuu keessaniin dura kanneen kanaa gadi mirkaneessa:

- Unka heeyyamaa odeeffannoo irratti hundaa'e kana dubbisuu keessan namni biroo isiniif yaa dubbisu,
- Waa'ee qo'annoo kanaa ibsii akka isiniif kenname fi gaafiin keessan akka deebi'e
- Yeroo kamiiyyuu gaafii dabalataa gaafachuu akka dandeessan hubachuu keessan
- Wanta isiniif ibsame hunda akka hubatanii fi qo'annoo kana irratti hirmaachuuf heeyyamamaa ta'u keessan
- Dhiibbaan altaawaa kamiiyyuu osoo isin irratti hin uumamiin yeroo kamiiyyuu heeyyama keessan kaasuu akka dandeessan hubachuu keessan. Yoo kana ta'e qabamee akka turu heeyyama yoon kenne malee kaayyoo qo'annoo kanaatiif odeeffannoo waa'ee koo sasaabame akka badu ni taasifama.

____________	____________	____________	☐☐☐☐
Maqaa hirmaataa fi maqaa gidduu	Mallattoo/ashaaraa hirmaataa	Guyyaa	Sa'aatii

____

____________	____________	____________	☐☐☐☐
Maqaa hojjataa marii heeyyamaa taasisee fi maqaa gidduu	Mallattoo hojjataa qo'annoo	Guyyaa	Sa'aatii

*Hirmaatoota dubbisuu hin dandeenyeef iddoo mallattoo kanaa gadi irratti guutaa

____________	____________	____________	☐☐☐☐
Maqaa raga fi maqaa gidduu	Malattoo raga	Guyyaa	Sa'aatii

*Ragaan walaba yoo ta'e yeroo waliigalaa adeemsa qo'annootti ni argama.

Unki heeyyamaa odeeffannoo irratti hundaa'e orjinaalli tokko kuusaadhaaf ni qabama. Unka mallatteeffame orjinaala kan biroo hirmaataadhaaf kennaa.

Pirotokoolii:Waraabbii ittisa TB mana keessaa:0:Ebla 19 bara 2013
Afanan Ingiliffaa:Barreeffama xiqqoo odeeffannoo hirmaataa fi unka heeyyama odeeffannoo irratti hundaa'e: waraabbii qo'annoo yeroo fi hojjii hojjattoota kunuunsa fayyaa 4, Guyyaa:Ebla 19 bara 2013
Raawwataa qo'annoo:-D/r Ahimad Badruu
Kan Raggaase:Biiroo Fayyaa Naannoo Oromiyaa IRB
Guyyaa ragga'e:13/09/2021
