# Supplementary material for: Pragmatic cluster-randomized trial of home-based preventive treatment for TB in Ethiopia and South Africa (CHIP-TB)
Source: Trials. 2023 Jul 25;24:475. doi: 10.1186/s13063-023-07514-7 (PMC10367260; doi:10.1186/s13063-023-07514-7)
Supplement: Supplementary file 3 — Additional file 3: Supplementary File 2b. Informed consent forms for study participants in South Africa. [file 13063_2023_7514_MOESM3_ESM.pdf]

PARTICIPANT INFORMATION LEAFLET AND INFORMED CONSENT FORM

ADULT INDEX PARTICIPANT: CONTROL CLINIC

PROTOCOL: A pragmatic cluster-randomized trial of community-based contact investigation and initiation of TB preventive therapy in South Africa and Ethiopia

SHORT TITLE: Home Based TPT

VERSION: Protocol Version 4.0, dated 27 April 2021
Informed Consent Form Version 4.0, dated 27 April 2021

PRINCIPAL INVESTIGATOR: Prof Salome Charalambous

TELEPHONE: 010 590 1300

Good day, I am_____________, a counselor /nurse from The Aurum Institute. I would like to invite you to consider participating in a research study. We are working together with the clinic staff here doing research about TB.

This research study will determine if the clinic or the home is the best place for children exposed to TB to get checked for and given medicine to prevent TB. All of the care your child will receive is by national guideline. You and your child do not need to do anything extra to be a part of this study. If you do not want to be a part of this study, you can still get all the care your child needs at the clinic.

The main risk of being in this study is loss in confidentiality. We have many preventive measures in place to make sure this does not happen.

I am inviting you to be part of this study because you have been diagnosed with TB. TB is spread by people coughing and others close by breathing in the air. Children who live in a house with someone who has TB are very likely to be infected with TB. That is why when someone has TB, the clinic sends someone to the house to check the other family members and see if they might have TB.

Our study is looking at how to get children a medicine that they need to keep from getting TB. Children can either be checked at the clinic or in the home. In this study, some children will be checked and given medicine at the clinic and some will be checked and given medicine in the home. People who are living with HIV are also very likely to be infected with TB. Sometimes, children may need HIV testing. Children at this clinic will be checked and given medicine at the clinic.

Being part of this study is your choice. You don't have to agree. You will still be able to get proper treatment at the clinic even if you don't want to be in this study. Also, if you agree and then change your mind, you can stop being in the study at any time.

If you agree to be part of this study, we will use the information in your clinic records to see how well the care given in the clinic is works for patients like you. We will ask to look at your clinic records until you complete your treatment for TB.

ADULT INDEX PARTICIPANT: CONTROL CLINIC

We will keep the information from your clinic chart confidential. Only the study and clinic staff will know this information. We will not disclose your TB status to your household members without your permission.

You do not need to do anything extra to be part of this research study. Through this research, we hope to improve the TB services the community receives.

If you feel the study staff has not treated you properly, you can get hold of Prof Salome Charalambous at The Aurum Institute – Tel: 010 590 1300

This study has been approved by the University of the Witwatersrand Human Research Ethics Committee (Wits HREC – Medical) and written approval has been granted by that committee. This study has also been approved by the Johns Hopkins IRB. Johns Hopkins will help The Aurum Institute monitor the study and analyze the data.

The study has been structured in accordance with the Declaration of Helsinki (last updated: October 2013) which deals with the recommendations guiding doctors in biomedical research involving human participants. I can obtain a copy for you if you wish to review it.

If you want any information regarding your rights as a participant, or have complaints regarding this study, you may contact:

Johannesburg:
Prof Clement Penny, Chairperson
The University of the Witwatersrand
Human Research Ethics Committee
Telephone number: (011) 717 2301

United States of America:
Johns Hopkins Medical – IRB X
The Johns Hopkins University
Institutional Review Board
Telephone number: (001) 410 955 3008

Do you have any questions or concerns before you make a decision about being in this study? If so, please ask me.

PARTICIPANT INFORMATION LEAFLET AND INFORMED CONSENT FORM

ADULT INDEX PARTICIPANT: CONTROL CLINIC

SIGNATURE PAGE

STATEMENT OF CONSENT

Before you sign this consent form, make sure of the following:

- You have read this informed consent form, or someone has read it to you.
- This study has been explained to you and had your questions answered.
- You understand you can ask more questions at any time.
- You have understood everything that has been explained to you and you consent to participate in this research study.
- You understand that you can without prejudice withdraw your consent at any time. If that happened, any data collected about me for the purposes of the study would be destroyed, unless I give consent for it to be retained.

Name of Participant: ____________________

______________________	______________________	______________	[ ][ ][ ][ ]
**Participant Name and Surname (Print)**	**Participant Signature / Thumbprint**	**Date (dd/mmm/yyyy)**	**Time**
______________________	______________________	______________	[ ][ ][ ][ ]
**Study Staff conducting consent discussion Name and Surname (Print)**	**Study Staff Signature**	**Date (dd/mmm/yyyy)**	Time

**For participants who are unable to read, also complete the signature block below:*

______________________	______________________	______________	[ ][ ][ ][ ]
***Witness' Name and Surname (Print)**	**Witness' Signature**	**Date (dd/mmm/yyyy)**	**Time**

**Witness is impartial and was present for the entire consent process.*

Retain one original Informed Consent Form on file. Offer participant the other original signed consent. Place a copy in medical records if applicable.

PARTICIPANT INFORMATION LEAFLET AND INFORMED CONSENT FORM

ADULT INDEX PARTICIPANT: INTERVENTION CLINIC

PROTOCOL: A pragmatic cluster-randomized trial of community-based contact investigation and initiation of TB preventive therapy in South Africa and Ethiopia

SHORT TITLE: Home Based TPT

VERSION: Protocol Version 4.0, dated 27 April 2021
Informed Consent Form Version 4.0, dated 27 April 2021

PRINCIPAL INVESTIGATOR: Prof Salome Charalambous

TELEPHONE: 010 590 1300

Good day, I am_____________, a counselor /nurse from The Aurum Institute. I would like to invite you to consider participating in a research study. We are working together with the clinic staff here doing research about TB.

This research study will determine if the clinic or the home is the best place for children exposed to TB to get checked for and given medicine to prevent TB. All of the care your child will receive is by national guideline. You and your child do not need to do anything extra to be a part of this study. If you do not want to be a part of this study, you can still get all the care your child needs at the clinic.

The main risk of being in this study is loss in confidentiality. We have many preventive measures in place to make sure this does not happen.

I am inviting you to be part of this study because you have been diagnosed with TB. TB is spread by people coughing and others close by breathing in the air. Children who live in a house with someone who has TB are very likely to be infected with TB. That is why when someone has TB, the clinic sends someone to the house to check the other family members and see if they might have TB.

Our study is looking at how to get children a medicine that they need to keep from getting TB. Children can either be checked at the clinic or in the home. In this study, some children will be checked and given medicine at the clinic and some will be checked and given medicine in the home. People who are living with HIV are also very likely to be infected with TB. Sometimes, children may need HIV testing. Children at this clinic will be checked and given medicine in the home.

Being part of this study is your choice. You do not have to agree. You will still be able to get proper treatment at the clinic even if you do not want to be in this study. Also, if you agree and then change your mind, you can stop being in the study at any time.

If you agree to be part of this study, we will:

1. Visit your home along with clinic staff who will check how you are doing. The clinic staff will also check the children who might be living in your homestead. If children do not have signs of TB, we will offer them medicine to keep them from getting TB. If children have signs of TB, we will ask their caregivers to bring

them to the clinic to be checked for TB. Before checking the children, we will ask consent from the child's parent/legal guardian. We will not check the children without this consent.

2. Use the information in your clinic records to see how well the care given to these children in the home, works for household members like yours. We will ask to look at your clinic records until you complete your treatment for TB.

We will keep the information from your clinic chart confidential. Only the study and clinic staff will know this information. We will not disclose your TB status to your household members without your permission.

You do not need to do anything extra to be part of this research study. Through this research, we hope to improve the TB services the community receives.

If you feel the study staff has not treated you properly, you can get hold of Prof Salome Charalambous at The Aurum Institute – Tel: 010 590 1300.

This study has been approved by the University of the Witwatersrand Human Research Ethics Committee (Wits HREC – Medical) and written approval has been granted by that committee. This study has also been approved by the Johns Hopkins IRB. Johns Hopkins will help The Aurum Institute monitor the study and analyze the data.

The study has been structured in accordance with the Declaration of Helsinki (last updated: October 2013) which deals with the recommendations guiding doctors in biomedical research involving human participants. I can obtain a copy for you if you wish to review it.

If you want any information regarding your rights as a participant, or have complaints regarding this study, you may contact:

Johannesburg:
Prof Clement Penny, Chairperson
The University of the Witwatersrand
Human Research Ethics Committee
Telephone number: (011) 717 2301

United States of America:
Johns Hopkins Medical – IRB X
The Johns Hopkins University
Institutional Review Board
Telephone number: (001) 410 955 3008

Do you have any questions or concerns before you make a decision about being in this study? If so, please ask me.

PARTICIPANT INFORMATION LEAFLET AND INFORMED CONSENT FORM

ADULT INDEX PARTICIPANT: INTERVENTION CLINIC

SIGNATURE PAGE

STATEMENT OF CONSENT

Before you sign this consent form, make sure of the following:

- You have read this informed consent form, or someone has read it to you.
- This study has been explained to you and had your questions answered.
- You understand you can ask more questions at any time.
- You have understood everything that has been explained to you and you consent to participate in this research study.
- You understand that you can without prejudice withdraw your consent at any time. If that happened, any data collected about me for the purposes of the study would be destroyed, unless I give consent for it to be retained.

Name of Participant: ___________________

____________________	____________________	____________	[ ][ ][ ][ ]
**Participant Name and Surname (Print)**	**Participant Signature / Thumbprint**	**Date (dd/mmm/yyyy)**	**Time**
____________________	____________________	____________	[ ][ ][ ][ ]
**Study Staff conducting consent discussion Name and Surname (Print)**	**Study Staff Signature**	**Date (dd/mmm/yyyy)**	**Time**

**For participants who are unable to read, also complete the signature block below:*

____________________	____________________	____________	[ ][ ][ ][ ]
***Witness' Name and Surname (Print)**	**Witness' Signature**	**Date (dd/mmm/yyyy)**	**Time**

**Witness is impartial and was present for the entire consent process.*

Retain one original Informed Consent Form on file. Offer participant the other original signed consent. Place a copy in medical records if applicable.

PARTICIPANT INFORMATION LEAFLET AND INFORMED CONSENT FORM
Healthcare Workers

PROTOCOL: A pragmatic cluster-randomized trial of community-based contact investigation and initiation of TB preventive therapy in South Africa and Ethiopia

SHORT TITLE: Home-Based TPT Time-and-Motion Assessment

VERSION: Protocol Version 4.0, dated 27 April 2021
Informed Consent Form Version 4.0 dated 27 April 2021

PRINCIPAL INVESTIGATOR: Prof Salome Charalambous

TELEPHONE: 010 590 1300

My name is ___________________________________. I am a researcher with the Aurum Institute in South Africa. I would like to invite you to consider participating in a research study.

In this study we want to learn about how much a program delivering home-based care for TB prevention costs the clinic. To be able to do that, we need to know how much time the program takes for health workers like yourself. If you choose to participate, you will be observed during one or more of your scheduled work shifts by a member of the study team. The study team member will record the types of activities you perform during your workday and how long you spend performing these tasks. This is called a **Time-and-Motion Study**. The main risk of being in this study is loss of confidentiality. We have many preventive measures in place to make sure this does not happen.

PROCEDURES

We are inviting healthcare workers taking part in the CHIP-TB Study to also to take part in a **Time-and-Motion Study.** If you want to take part, you will be observed when you work at the clinic by the researcher. The researcher will write down the different activities you perform during your workday. The person will also write down how long you take to do it.

The research team member may observe you for a part of your workday or all of your workday. You may be observed for only one day, or on a few days. The observation days will be randomly selected. “Random selection” is like drawing numbers from a hat. The research team will try to let you know before the time what day or day(s) you can expect to be observed and have your work activities written down. These observations should not take much of your time.

You may also be asked to write down your own work activities and the time that you take to do the activities. We will give you a form to write down the activities that you do. We will also provide

instructions on how to record your activities and how long it takes you to do them. These forms will not take much time to fill out.

The information we learn by observing you will allow us to calculate the costs of a home visit and a clinic visit. This information can help the government decide if home visits are worthwhile. This may help groups like the World Health Organization create new guidelines.

RISKS AND BENEFITS
We do not think that being part of this study will create significant risks for you. The main risk of being in this study is loss of confidentiality. It is not possible to guarantee complete privacy. We will try to make the records as private as possible. Your name will not be attached to any report we create based on these observations. The information we collect as part of this research project will only be used for this research study and will not be shared with your supervisor. Your information will not be used in future research studies.

You will not receive any direct benefit from participating in this study. The data collected from your participation may help to improve TB and HIV prevention services in your community and in other communities.

You will not be paid to take part in this study.

VOLUNTARY PARTICIPATION
You do not have to agree to be in this study. If you do not want to join the study, it will not affect your job. And, if you do participate, your job will not be affected by your time or your answers. You can decide to stop taking part in the study at any time without giving a reason.

If you have any questions about your rights as a research participant, or if you think you have not been treated fairly, you may contact Prof Salome Charalambous at the Aurum Institute – Tel: 010 5901300.

ETHICAL APPROVAL
This study protocol has been approved by the University of the Witwatersrand Human Research Ethics Committee (Wits HREC – Medical) and written approval has been granted by that committee. This study has also been approved by the Johns Hopkins IRB. Johns Hopkins will help Aurum Institute monitor the study and analyze the data. The study has been structured in accordance with the Declaration of Helsinki (last updated: October 2013) which deals with the recommendations guiding doctors in biomedical research involving human participants. I can obtain a copy for you if you wish to review it.

If you want any information regarding your rights as a research participant, or have complaints regarding this research study, you may contact:

Johannesburg:
Prof Clement Penny, Chairperson
The University of the Witwatersrand
Human Research Ethics Committee
Telephone number: (011) 717 2301

PARTICIPANT INFORMATION LEAFLET AND INFORMED CONSENT FORM Healthcare Workers

United States of America:
Johns Hopkins Medical – IRB X
The Johns Hopkins University
Institutional Review Board
Telephone number: (001) 410 955 3008

This independent committee is established to help protect the rights of research participants and gave written approval for the study protocol.

PARTICIPANT INFORMATION LEAFLET AND INFORMED CONSENT FORM Healthcare Workers

SIGNATURE PAGE

STATEMENT OF CONSENT

Before you sign this consent form, make sure of the following:

- You have read this informed consent form, or someone has read it to you.
- This study has been explained to you and had your questions answered.
- You understand you can ask more questions at any time.
- You have understood everything that has been explained to you and you consent to participate in this research study.
- You understand that you can without prejudice withdraw your consent at any time. If that happened, any data collected about me for the purposes of the study would be destroyed, unless I give consent for it to be retained.

**Participant's Name and Surname (Print)**	**Participant's Signature / Thumbprint**	**Date (dd/mmm/yyyy)**	**Time**

**Study Staff conducting consent discussion Name and Surname (Print)**	**Study Staff Signature**	**Date (dd/mmm/yyyy)**	**Time**

**For participants who are unable to read, also complete the signature block below:*

**Witness' Name and Surname (Print)**	**Witness' Signature**	**Date (dd/mmm/yyyy)**	**Time**

**Witness is impartial and was present for the entire consent process.*

Retain one original Informed Consent Form on file. Offer participant the other original signed consent.

PARENT/LEGAL GUARDIAN INFORMATION LEAFLET AND INFORMED CONSENT FORM FOR HOUSEHOLD CONTACTS OF TUBERCULOSIS INDEX PARTICIPANTS

CHILD CONTACT: CONTROL CLINIC

PROTOCOL: A pragmatic cluster-randomized trial of community-based contact investigation and initiation of TB preventive therapy in South Africa and Ethiopia

SHORT TITLE: Home Based TPT

VERSION: Protocol Version 4.0, dated 27 April 2021
Informed Consent Form Version 4.0, dated 27 April 2021

PRINCIPAL INVESTIGATOR: Prof Salome Charalambous

TELEPHONE: 010 590 1300

Good day, I am_____________, a nurse/community health worker from The Aurum Institute. I would like to invite you to consider your child's participation in a research study. We are working together with the clinic staff here doing research about TB.

This research study will determine if the clinic or the home is the best place for children exposed to TB to get checked for and given medicine to prevent TB. All of the care your child will receive is by national guideline. You and your child do not need to do anything extra to be a part of this study. If you do not want to be a part of this study, you can still get all the care your child needs at the clinic.

The main risk of being in this study is loss in confidentiality. We have many preventive measures in place to make sure this does not happen.

I am inviting your child to be part of this study because someone in your household has TB. TB is spread by people coughing and others close by breathing in the air. Children who live in a house with someone who has TB are very likely to be infected with TB. That is why when someone has TB, the clinic sends someone to the house to check the other family members and see if they might have TB.

Our study is looking at how well a home visit program works to get children a medicine that they need to keep from getting TB. This medicine is recommended by the South African Government for children who live with someone with TB. You can decide to accept or not accept this medicine. Before getting this medicine, children need to be checked for TB and, when appropriate, HIV. This is because people who are living with HIV are also very likely to be infected with TB. The check can happen at the clinic or in the home. In this study, we are looking at whether it will be useful for clinic staff to make home visits. Some children will be

checked and given medicine at the clinic and some will be checked and given medicine in the home. Children at this clinic will be checked and given medicine at the clinic.

You may be randomly selected to take part in two brief costing interviews. "Random selection' is like choosing people by flipping a coin. If you are selected, the interviews will take place on the telephone or in person. Each interview will each be about 30 minutes long. We will ask questions about how much it costs you and your family to bring your child to the clinic. The first interview with happen soon after your child is checked for TB. The second interview will be in about 3 months, after your child finishes treatment.

We also want to know how much the program is costing the clinic. Some clinic visits will be "randomly selected" for observation by a research staff member. All the information the researcher sees or hears will be kept confidential/secret. You will be asked at the start of your visit if it is ok with you that the researcher stays in the room. It is ok to say no. It will not affect the care you and your child receive in the clinic. It will also not affect your participation in the study.

Being part of this study is your choice. You do not have to agree. Your child will still be able to get proper treatment at the clinic even if you do not want to be in this study. Also, if you agree and then change your mind, you can stop being in the study at any time.

If you agree to be part of this study, we will:

1. Explain the study to your child (if they are 8 years of age or older) and ask if they choose to agree.
2. Use the information in your child's clinic records to see how well the care given in the clinic works for patients. We will ask to look over your child's clinic record for the next year.
3. We *may* contact you to participate in an interview about how much visiting the clinic for your child's care costs your household.

We will keep the information from your child's clinic chart confidential. Only the study and clinic staff will know this information.

Your child does not need to do anything extra to be part of this research study. Through this research, we hope to improve the TB services the community receives.

You will not be paid to take part in this study. If you are selected for the interviews, we will pay you 50 ZAR in cell phone airtime, for each interview you complete, to cover your time expenses.

If you feel the study staff has not treated you properly, you can get hold of Prof Salome Charalambous at The Aurum Institute – Tel: 010 590 1300.

This study has been approved by the University of the Witwatersrand Human Research Ethics Committee (Wits HREC – Medical) and written approval has been granted by that committee. This study has also been approved by the Johns Hopkins IRB. Johns Hopkins will help The Aurum Institute monitor the study and analyze the data.

The study has been structured in accordance with the Declaration of Helsinki (last updated: October 2013) which deals with the recommendations guiding doctors in biomedical research involving human participants. I can obtain a copy for you if you wish to review it.

If you want any information regarding your rights as a participant, or have complaints regarding this study, you may contact:

Johannesburg:
Prof Clement Penny, Chairperson
The University of the Witwatersrand
Human Research Ethics Committee
Telephone number: (011) 717 2301

United States of America:
Johns Hopkins Medical – IRB X
The Johns Hopkins University
Institutional Review Board
Telephone number: (001) 410 955 3008

Do you have any questions or concerns before you make a decision about being in this study? If so, please ask me.

PARENT/LEGAL GUARDIAN INFORMATION LEAFLET AND INFORMED CONSENT FORM FOR HOUSEHOLD CONTACTS OF TUBERCULOSIS INDEX PARTICIPANTS

CHILD CONTACT: CONTROL CLINIC

SIGNATURE PAGE FOR THE COLLECTION OF YOUR CHILD'S RECORDS

STATEMENT OF CONSENT

Before you sign this consent form for the collection of your child's records, make sure of the following:

- You have read this informed consent form, or someone has read it to you.
- This study has been explained to you and had your questions answered.
- You understand you can ask more questions at any time.
- You have understood everything that has been explained to you and you consent that your child can participate in this research study.
- You understand that you can without prejudice withdraw your consent at any time. If that happened, any data collected about you and your childfor the purposes of the study would be destroyed, unless you give consent for it to be retained.

Name of Parent / Legal Guardian ____________________________

Name of Child: _________________ Age of child_____________

Do you agree for your child to be in this study? YES_______ NO_______ (Please initial)

________________	________________	________	
**Parent/Legal Guardian Name and Surname (Print)**	**Parent/Legal Guardian Signature/ Thumbprint**	**Date (dd/mmm/yyyy)**	**Time**
________________	________________	________	
**Study Staff conducting consent Name and Surname (Print)**	**Study Staff conducting consent Signature/Thumbprint**	**Date (dd/mmm/yyyy)**	**Time**

**For participants who are unable to read, also complete the signature block below:*

________________	________________	________	
**Witness' Name and Surname (Print)**	**Witness' Signature**	**Date (dd/mmm/yyyy)**	**Time**

**Witness is impartial and was present for the entire consent process.*

Retain one original Informed Consent Form on file. Offer participant the other original signed consent. Place a copy in medical records if applicable.

PARENT/LEGAL GUARDIAN INFORMATION LEAFLET AND INFORMED CONSENT FORM FOR HOUSEHOLD CONTACTS OF TUBERCULOSIS INDEX PARTICIPANTS

CHILD CONTACT: CONTROL CLINIC

SIGNATURE PAGE FOR THE COSTING INTERVIEW

STATEMENT OF CONSENT

Before, you sign this consent form to take part in the costing interview, make sure of the following.

- You have read this informed consent form, or someone has read it to you.
- This study, including the costing interviews, has been explained to you and you had your questions answered.
- You understand you can ask more questions at any time.
- You have understood everything that has been explained to you and you consent to participate in the costing interviews.
- You understand that you can without prejudice withdraw your consent at any time. If that happened, any data collected about you and your child for the purposes of the study would be destroyed, unless you give consent for it to be retained.

If you **AGREE** to take part in an interview about personal costs related to your child's treatment, please sign your name or make your mark below.

**Participant's Name and Surname (Print)**	**Participant's Signature / Thumbprint**	**Date (dd/mmm/yyyy)**	**Time**
**Study Staff conducting consent discussion Name and Surname (Print)**	**Study Staff Signature**	**Date (dd/mmm/yyyy)**	**Time**

**For participants who are unable to read, also complete the signature block below:*

**Witness' Name and Surname (Print)**	**Witness' Signature**	**Date (dd/mmm/yyyy)**	**Time**

**Witness is impartial and was present for the entire consent process.*

Retain one original Informed Consent Form on file. Offer participant the other original signed consent. Place a copy in medical records if applicable.

PARENT/LEGAL GUARDIAN INFORMATION LEAFLET AND <u>INFORMED CONSENT</u> FORM FOR HOUSEHOLD CONTACTS OF TUBERCULOSIS INDEX PARTICIPANTS

<u>CHILD</u> CONTACT: <u>INTERVENTION</u> CLINIC

PROTOCOL: A pragmatic cluster-randomized trial of community-based contact investigation and initiation of TB preventive therapy in South Africa and Ethiopia

SHORT TITLE: Home Based TPT

VERSION: Protocol Version 4.0, dated 27 April 2021
Informed Consent Form Version 4.0, dated 27 April 2021

PRINCIPAL INVESTIGATOR: Prof Salome Charalambous

TELEPHONE: 010 590 1300

Good day, I am_____________, a nurse/community health worker from The Aurum Institute. I would like to invite you to consider your child's participation in a research study. We are working together with the clinic staff here doing research about TB.

This research study will determine if the clinic or the home is the best place for children exposed to TB to get checked for and given medicine to prevent TB. All of the care your child will receive is by national guideline. You and your child do not need to do anything extra to be a part of this study. If you do not want to be a part of this study, you can still get all the care your child needs at the clinic.

The main risk of being in this study is loss in confidentiality. We have many preventive measures in place to make sure this does not happen.

I am inviting your child to be part of this study because someone in your household has TB. TB is spread by people coughing and others close by breathing in the air. Children who live in a house with someone who has TB are very likely to be infected with TB. That is why when someone has TB, the clinic sends someone to the house to check the other family members and see if they might have TB.

Our study is looking at how well a home visit program works to get children a medicine that they need to keep from getting TB. This medicine is recommended by the South African Government for children who live with someone with TB. You can decide to accept or not accept this medicine. Before getting this medicine, children need to be checked for TB and, when appropriate, HIV. This is because people who are living with HIV are also very likely to be infected with TB. The check can happen at the clinic or in the home. In this study, we are looking at whether it will be useful for clinic staff to make home visits. Some children will be checked and given medicine at the clinic and some will be checked and given medicine in the home. Children who get care at this clinic will be checked and given medicine in the home.

You may be randomly selected to take part in two brief costing interviews. "Random selection' is like choosing people by flipping a coin. If you are selected, the interviews will take place on the telephone or in person. Each interview will each be about 30 minutes long. We will ask questions about how much it costs you and your family to participate in the home visit program. The first interview with happen soon after your child is checked for TB. The second interview will be in about 3 months, after your child finishes treatment.

We also want to know how much the program is costing the clinic. Some home visits will be "randomly selected" for observation by a research staff member. All the information the researcher sees or hears will be kept confidential/secret. You will be asked at the start of your visit if it is ok with you that the researcher stays in the room. It is ok to say no. It will not affect the care you and your child receive in the clinic. It will also not affect your participation in the study.

Being part of this study is your choice. You do not have to agree. Your child will still be able to get proper treatment at the clinic even if you do not want to be in this study. Also, if you agree and then change your mind, you can stop being in the study at any time.

If you agree to be part of this study, we will:

1. Explain the study to your child (if they are 8 years of age or older) and ask if they choose to agree.
2. Visit your home and check your child for symptoms of TB. We will only visit your home after getting permission from your household member who has TB.
3. If your child does not have signs of TB, we will offer them medicine to keep them from getting TB. If your child has signs of TB, we will ask you to bring them to the clinic to be checked for TB.
4. Use the information in your child's clinic records to see how well the care given in the home, works for patients. We will ask to look over your child's clinic record for the next year.
5. We *may* contact you to participate in an interview about how much the home visit for your child's care costs your household.

We will keep the information from your child's clinic chart confidential. Only the study and clinic staff will know this information.

Your child does not need to do anything extra to be part of this research study. Through this research, we hope to improve the TB services the community receives.

You will not be paid to take part in this study. If you are selected for the interviews, we will pay you 50 ZAR in cell phone airtime, for each interview you complete, to cover your time expenses.

If you feel the study staff has not treated you properly, you can get hold of Prof Salome Charalambous at The Aurum Institute – Tel: 010 590 1300.

This study has been approved by the University of the Witwatersrand Human Research Ethics Committee (Wits HREC – Medical) and written approval has been granted by that committee.

This study has also been approved by the Johns Hopkins IRB. Johns Hopkins will help The Aurum Institute monitor the study and analyze the data.

The study has been structured in accordance with the Declaration of Helsinki (last updated: October 2013) which deals with the recommendations guiding doctors in biomedical research involving human participants. I can obtain a copy for you if you wish to review it.

If you want any information regarding your rights as a participant, or have complaints regarding this study, you may contact:

Johannesburg:
Prof Clement Penny, Chairperson
The University of the Witwatersrand
Human Research Ethics Committee
Telephone number: (011) 717 2301

United States of America:
Johns Hopkins Medical – IRB X
The Johns Hopkins University
Institutional Review Board
Telephone number: (001) 410 955 3008

Do you have any questions or concerns before you make a decision about being in this study? If so, please ask me.

PARENT/LEGAL GUARDIAN INFORMATION LEAFLET AND INFORMED CONSENT FORM FOR HOUSEHOLD CONTACTS OF TUBERCULOSIS INDEX PARTICIPANTS

CHILD CONTACT: INTERVENTION CLINIC

SIGNATURE PAGE FOR THE HOME-BASED TB PREVENTION INTERVENTION

STATEMENT OF CONSENT

Before you sign this consent form for the home-based TB prevention intervention, make sure of the following:

- You have read this informed consent form, or someone has read it to you.
- This study has been explained to you and had your questions answered.
- You understand you can ask more questions at any time.
- You have understood everything that has been explained to you and you consent that your child can participate in this research study.
- You understand that you can without prejudice withdraw your consent at any time. If that happened, any data collected about you and your childfor the purposes of the study would be destroyed, unless you give consent for it to be retained.

Name of Parent / Legal Guardian ______________________________

Name of Child: _________________ Age of child_____________

Do you agree for your child to be in this study? YES_______ NO_______ (Please initial)

________________________	________________________	___________	☐☐☐☐
**Parent/Legal Guardian Name and Surname (Print)**	**Parent/Legal Guardian Signature/ Thumbprint**	**Date (dd/mmm/yyyy)**	**Time**
________________________	________________________	___________	☐☐☐☐
**Study Staff conducting consent Name and Surname (Print)**	**Study Staff conducting consent Signature/Thumbprint**	**Date (dd/mmm/yyyy)**	**Time**

**For participants who are unable to read, also complete the signature block below:*

________________________	________________________	___________	☐☐☐☐
**Witness' Name and Surname (Print)**	**Witness' Signature**	**Date (dd/mmm/yyyy)**	**Time**

**Witness is impartial and was present for the entire consent process.*

Retain one original Informed Consent Form on file. Offer participant the other original signed consent. Place a copy in medical records if applicable.

PARENT/LEGAL GUARDIAN INFORMATION LEAFLET AND INFORMED CONSENT FORM FOR HOUSEHOLD CONTACTS OF TUBERCULOSIS INDEX PARTICIPANTS

CHILD CONTACT: INTERVENTION CLINIC

SIGNATURE PAGE FOR THE COSTING INTERVIEW

STATEMENT OF CONSENT

Before, you sign this consent form to take part in the costing interview, make sure of the following

- You have read this informed consent form, or someone has read it to you.
- This study, including the costing interviews, has been explained to you and you had your questions answered.
- You understand you can ask more questions at any time.
- You have understood everything that has been explained to you and you consent to participate in the costing interviews.
- You understand that you can without prejudice withdraw your consent at any time. If that happened, any data collected about you and your child for the purposes of the study would be destroyed, unless you give consent for it to be retained.

If you **AGREE** to take part in an interview about personal costs related to your child's treatment, please sign your name or make your mark below.

**Participant's Name and Surname (Print)**	**Participant's Signature / Thumbprint**	**Date (dd/mmm/yyyy)**	**Time**

**Study Staff conducting consent discussion Name and Surname (Print)**	**Study Staff Signature**	**Date (dd/mmm/yyyy)**	**Time**

**For participants who are unable to read, also complete the signature block below:*

**Witness' Name and Surname (Print)**	**Witness' Signature**	**Date (dd/mmm/yyyy)**	**Time**

**Witness is impartial and was present for the entire consent process.*

Retain one original Informed Consent Form on file. Offer participant the other original signed consent. Place a copy in medical records if applicable.
